# Supplementary figures and images for: Multi-objective numerical optimization of 3D-printed polylactic acid bio-metamaterial based on topology, filling pattern, and infill density via fatigue lifetime and mass
Source: PLoS One. 2023 Sep 27;18(9):e0291021. doi: 10.1371/journal.pone.0291021 (PMC10529563; doi:10.1371/journal.pone.0291021)

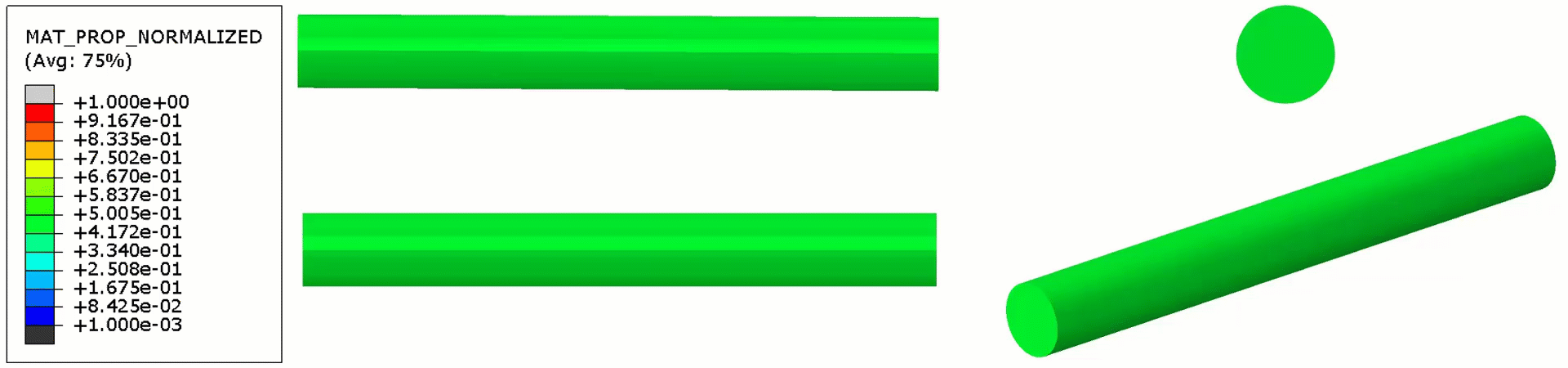

Supplement: S1 Fig — (GIF) [file pone.0291021.s001.gif]
