# Supplementary material for: Multi-objective numerical optimization of 3D-printed polylactic acid bio-metamaterial based on topology, filling pattern, and infill density via fatigue lifetime and mass
Source: PLoS One. 2023 Sep 27;18(9):e0291021. doi: 10.1371/journal.pone.0291021 (PMC10529563; doi:10.1371/journal.pone.0291021)
Supplement: Attachment — Submitted filename: PONE-D-23-02761_R1-1.pdf [file pone.0291021.s005.pdf]

PLOS ONE

Multi-objective numerical optimization of 3D-printed polylactic acid bio-metamaterial based on topology, filling pattern, and infill density via fatigue lifetime and mass

--Manuscript Draft--

**Manuscript Number:**	PONE-D-23-02761R1
**Article Type:**	Research Article
**Full Title:**	Multi-objective numerical optimization of 3D-printed polylactic acid bio-metamaterial based on topology, filling pattern, and infill density via fatigue lifetime and mass
**Short Title:**	Multi-objective numerical optimization of 3D-printed polylactic acid bio-metamaterial
**Corresponding Author:**	Mohammad Azadi<br>Semnan University<br>Semnan, Semnan IRAN, ISLAMIC REPUBLIC OF
**Keywords:**	Metamaterial; multi-objective optimization; Fatigue lifetime; 3D printing quality; Finite element modeling
**Abstract:**	Infill parameters are significant with regard to the overall cost and saving material while printing a 3D model. When it comes to printing time, we can decrease the printing time by altering the infill, which also reduces the total process extent. Choosing the right filling parameters affects the strength of the printed model. In this research, the effect of filling density and infill pattern on the fatigue lifetime of cylindrical polylactic acid (PLA) samples was investigated with finite element modeling and analysis. This causes the lattice structure to be considered macro-scale porosity in the additive manufacturing process. Due to the need for multi-objective optimization of several functions at the same time and the inevitable sacrifice of other objectives, the decision was to obtain a set of compromise solutions according to the Pareto-optimal solution technique or the Pareto non-inferior solution approach. As a result, a horizontally printed rectangular pattern with 60% filling was preferred over the four patterns including honeycomb, triangular, regular octagon, and irregular octagon by considering the sum of mass changes and fatigue lifetime changes, and distance from the optimal point, which is the lightest structure with the maximum fatigue lifetime as an objective function with an emphasis on mass as an important parameter in designing scaffolds and biomedical structures. A new structure was also proposed by performing a structural optimization process using computer-aided design tools and also, computer-aided engineering software by Dassault systems. Finally, the selected samples were printed and their 3D printing quality was investigated using field emission scanning electron microscopy inspection.
**Order of Authors:**	Ali Dadashi
	Mohammad Azadi
**Response to Reviewers:**	Please check the file uploaded for answers to the reviewers' comments.
**Additional Information:**	
**Question**	**Response**
**Financial Disclosure**<br><br>Enter a financial disclosure statement that describes the sources of funding for the work included in this submission. Review the submission guidelines for detailed requirements. View published research articles from *PLOS ONE* for specific examples.<br><br>This statement is required for submission	The authors would like to acknowledge the financial support of the Iran Small Industries and Industrial Parks Organization (ISIPO) for this project under grant number of 23607.

Powered by Editorial Manager® and ProduXion Manager® from Aries Systems Corporation

and **will appear in the published article** if the submission is accepted. Please make sure it is accurate.<br><br>**Unfunded studies**<br>Enter: *The author(s) received no specific funding for this work.*<br><br>**Funded studies**<br>Enter a statement with the following details:<br>• Initials of the authors who received each award<br>• Grant numbers awarded to each author<br>• The full name of each funder<br>• URL of each funder website<br>• Did the sponsors or funders play any role in the study design, data collection and analysis, decision to publish, or preparation of the manuscript?<br>• **NO** - Include this sentence at the end of your statement: *The funders had no role in study design, data collection and analysis, decision to publish, or preparation of the manuscript.*<br>• **YES** - Specify the role(s) played.<br><br>* typeset	
**Competing Interests**<br><br>Use the instructions below to enter a competing interest statement for this submission. On behalf of all authors, disclose any competing interests that could be perceived to bias this work—acknowledging all financial support and any other relevant financial or non-financial competing interests.<br><br>This statement is required for submission and **will appear in the published article** if the submission is accepted. Please make sure it is accurate and that any funding sources listed in your Funding Information later in the submission form are also declared in your Financial Disclosure statement.<br><br>View published research articles from *PLOS ONE* for specific examples.	The authors illustrate that they have no competing financial interests or personal relationships that could have appeared to influence this work.

**NO authors have competing interests**<br><br>Enter: *The authors have declared that no competing interests exist.*<br><br>**Authors with competing interests**<br><br>Enter competing interest details beginning with this statement:<br><br>*I have read the journal's policy and the authors of this manuscript have the following competing interests: [insert competing interests here]*<br><br>* typeset	
**Ethics Statement**<br><br>Enter an ethics statement for this submission. This statement is required if the study involved:<br><br>• Human participants<br>• Human specimens or tissue<br>• Vertebrate animals or cephalopods<br>• Vertebrate embryos or tissues<br>• Field research<br><br>Write "N/A" if the submission does not require an ethics statement.<br><br>General guidance is provided below. Consult the submission guidelines for detailed instructions. **Make sure that all information entered here is included in the Methods section of the manuscript.**	N/A

**Format for specific study types**<br><br>**Human Subject Research (involving human participants and/or tissue)**<br>• Give the name of the institutional review board or ethics committee that approved the study<br>• Include the approval number and/or a statement indicating approval of this research<br>• Indicate the form of consent obtained (written/oral) or the reason that consent was not obtained (e.g. the data were analyzed anonymously)<br><br>**Animal Research (involving vertebrate animals, embryos or tissues)**<br>• Provide the name of the Institutional Animal Care and Use Committee (IACUC) or other relevant ethics board that reviewed the study protocol, and indicate whether they approved this research or granted a formal waiver of ethical approval<br>• Include an approval number if one was obtained<br>• If the study involved *non-human primates*, add *additional details* about animal welfare and steps taken to ameliorate suffering<br>• If anesthesia, euthanasia, or any kind of animal sacrifice is part of the study, include briefly which substances and/or methods were applied<br><br>**Field Research**<br><br>Include the following details if this study involves the collection of plant, animal, or other materials from a natural setting:<br>• Field permit number<br>• Name of the institution or relevant body that granted permission	
**Data Availability**<br><br>Authors are required to make all data underlying the findings described fully available, without restriction, and from the time of publication. PLOS allows rare exceptions to address legal and ethical concerns. See the PLOS Data Policy and FAQ for detailed information.	Yes - all data are fully available without restriction

Powered by Editorial Manager® and ProduXion Manager® from Aries Systems Corporation

A Data Availability Statement describing where the data can be found is required at submission. Your answers to this question constitute the Data Availability Statement and **will be published in the article**, if accepted.<br><br>**Important:** Stating 'data available on request from the author' is not sufficient. If your data are only available upon request, select 'No' for the first question and explain your exceptional situation in the text box.<br><br>Do the authors confirm that all data underlying the findings described in their manuscript are fully available without restriction?	
**Describe where the data may be found in full sentences. If you are copying our sample text, replace any instances of XXX with the appropriate details.**<br><br>• If the data are **held or will be held in a public repository**, include URLs, accession numbers or DOIs. If this information will only be available after acceptance, indicate this by ticking the box below. For example: *All XXX files are available from the XXX database (accession number(s) XXX, XXX.).*<br>• If the data are all contained **within the manuscript and/or Supporting Information files**, enter the following: *All relevant data are within the manuscript and its Supporting Information files.*<br>• If neither of these applies but you are able to provide **details of access elsewhere**, with or without limitations, please do so. For example:<br><br>*Data cannot be shared publicly because of [XXX]. Data are available from the XXX Institutional Data Access / Ethics Committee (contact via XXX) for researchers who meet the criteria for access to confidential data.*<br><br>*The data underlying the results presented in the study are available from (include the name of the third party*	All relevant data are within the manuscript and its Supporting Information files.

and contact information or URL).<br>• This text is appropriate if the data are owned by a third party and authors do not have permission to share the data.<br><br>* typeset	
Additional data availability information:	

Powered by Editorial Manager® and ProduXion Manager® from Aries Systems Corporation

Manuscript Click here to access/download;Manuscript;Optimization-v20 - Clean.docx

Multi-objective numerical optimization of 3D-printed polylactic acid bio-metamaterial based on topology, filling pattern, and infill density via fatigue lifetime and mass

Ali Dadashi[1] and Mohammad Azadi[1*]

[1] Research Laboratory of Advanced Materials Behavior (AMB), Faculty of Mechanical Engineering, Semnan University, Semnan, Iran
[*]Corresponding Author, Email: m_azadi@semnan.ac.ir

Abstract

Infill parameters are significant with regard to the overall cost and saving material while printing a 3D model. When it comes to printing time, we can decrease the printing time by altering the infill, which also reduces the total process extent. Choosing the right filling parameters affects the strength of the printed model. In this research, the effect of filling density and infill pattern on the fatigue lifetime of cylindrical polylactic acid (PLA) samples was investigated with finite element modeling and analysis. This causes the lattice structure to be considered macro-scale porosity in the additive manufacturing process. Due to the need for multi-objective optimization of several functions at the same time and the inevitable sacrifice of other objectives, the decision was to obtain a set of compromise solutions according to the Pareto-optimal solution technique or the Pareto non-inferior solution approach. As a result, a horizontally printed rectangular pattern with 60% filling was preferred over the four patterns including honeycomb, triangular, regular octagon, and irregular octagon by considering the sum of mass changes and fatigue lifetime changes, and distance from the optimal point, which is the lightest structure with the maximum fatigue lifetime as an objective function with an emphasis on mass as an important parameter in designing scaffolds and biomedical structures. A new structure was also proposed by performing a structural optimization process using computer-aided design tools and also, computer-aided engineering software by Dassault systems. Finally, the selected samples were printed and their 3D printing quality was investigated using field emission scanning electron microscopy inspection.

Keywords: Metamaterial; Multi-objective optimization; Fatigue lifetime; 3D printing quality; Finite element modeling

1) Introduction

The functionality of a fabricated component relies on the combination of properties and geometry, achieved through a series of fabrication techniques [1]. Additive manufacturing (AM) processes offer advantages such as complex 3D geometries and flexible construction of layered structures [2]. Tailoring the build formulation allows for gradual changes in composition or lattice structures [3-4]. For instance, Kumar et al. [5-6] compared multi-blended and hybrid blended PLA matrix in 3D-printed tensile specimens, finding superior mechanical and morphological properties in the multi-blended matrix, reflecting the growing interest in exploring the potential of additive manufacturing.

AM techniques enable the fabrication of metamaterials with unique properties, such as negative Poisson ratio and high strength [7-9]. These metamaterials exhibit extraordinary functions in acoustics, electromagnetics, and mechanics [10]. Bio-metamaterials, designed with geometrical structures at smaller scales, mimic the properties of living tissues and are applied in orthopedic biomaterials for bone tissue regeneration [11]. Although the feasibility and reliability of these processes are still recognized as a challenge [12]. Wu et al. [13] developed a novel mechanical metamaterial with lateral expansion, validated through simulations and experiments. Agrawal et al. [14] proposed a robust topology optimization method for negative Poisson ratio metamaterials, ensuring stable designs with low variation. Researchers have utilized topology optimization (TO) and additive manufacturing (AM) to enhance the design and fabrication of structurally optimized cellular structures [15-16]. The integration of artificial intelligence (AI) and AM has introduced a new design principle, enabling efficient structure and component design through machine learning techniques [17-21]. Qian et al. [22] employed an artificial neural network-based inverse design method, demonstrating improved efficiency and reduced training data requirements for designing architected composite parts. Their approach showcased superior performance compared to other machine-learning approaches.

In order to further increase the mechanical properties of parts fabricated by AM, designing and optimizing approaches have been introduced and developed at various scales. Specifically, on the macroscale, structural optimizations including shape, size, and topology optimization techniques could be considered for guiding the component design [23-24]. Between these methods, polymeric composites can achieve their maximum potential by AM-driven TO [25]. Manufacturing constraints such as minimum size and connectivity limit the optimization according to the manufacturing ability [26]. The anisotropic material property, besides manufacturing constraints, is another parameter that should be applied during the process of designing and optimizing [27]. A transversely isotropic material model and solid anisotropic material with penalization coupled with an AM-driven topology optimization technique were proposed by Li et al. [28-29]. Due to the layered fabrication process, they achieved mechanical improvement in stiffness and strength because of taking advantage of anisotropy. Wu et al. [30] presented a novel method for generating simultaneously optimized solid shell and porous infill in the context of maximum stiffness topology optimization. A material interpolation model, upon which the compliance is minimized, unified the resulting intermediate density distributions while all the fibers follow the direction of the principal stress.

3D printing process parameters have a significant impact on the mechanical properties of printed parts, including printing temperature, speed, filling density, pattern, layer thickness, material properties, surface roughness, and layer thickness [31-33]. Researchers have conducted several studies to further clarify these effects [34-35]. Manufacturing 3D-printed

products requires a clear understanding of the mechanical characteristics of 3D-printed components [36]. Sood et al. [37] found that increasing the layer thickness in ABS 3D-printed components improved impact and bending properties, but had an inverse relationship with tensile performance. Fernandez Vicente et al. [38] examined the influence of different infill patterns on the tensile performance of ABS 3D-printed parts. They found that ABS components with line and honeycomb infill patterns exhibited higher tensile strength compared to those with a rectilinear infill pattern. Baptista and Guedes [39] investigated the impact of infill density and patterns on the static and fatigue characteristics of scaffolds. Lower infill density resulted in higher yield stress and showed no significant fatigue damage after 3600 load cycles. Moreover, the influence of infill line distance on the compressive elastic behavior of 3D printed circular samples was investigated by Pepelnjak et al. [40] They observed a significant difference in the first cycle response and an alteration of stiffness due to elastic and plastic deformation under cyclic.

Fountas et al. [41] examined the performance of different swarm-based evolutionary algorithms in optimizing single and multi-objective problems related to additive manufacturing, specifically fused deposition modeling (FDM). The results showed that algorithms do not perform equally when applied to different optimization problems, and the quality of Pareto non-dominated solutions is an important indicator for multi-objective problems. Yodo and Dey [42] introduced multi-objective optimization methods based on evolutionary algorithms to optimize FDM process parameters, leading to Pareto-optimal solutions for decision-makers.

So far, the literature has been reviewed and by summarizing the presented materials, it can be said that researchers have focused on the following topics:

- The extraordinary properties of meta-materials [8-9], [13]
- Providing optimization algorithms to improve mechanical behavior [17-23]
- Designing optimized structures in different dimensions [26-30]
- Applying additive manufacturing conditions in the optimization process [31-39], [43-46]
- Introducing Pareto-optimal solutions for decision-makers [41-42]

The article discusses the multi-objective numerical optimization of 3D-printed polylactic acid (PLA) bio-metamaterial. The optimization takes into account the factors of topology, filling pattern, and infill density in order to improve the fatigue lifetime and mass of the material. Therefore, the novelties of the study are as follows,

- The use of multi-objective optimization to find the optimum value of topology, filling pattern, and infill density for 3D-printed polylactic acid bio-metamaterial.
- The consideration of both fatigue lifetime and mass in the optimization process.
- The use of field emission scanning electron microscopy (FESEM) images to investigate the dimensional accuracy of 3D-printed components.

2) Material and Methodology

2-1) Material Properties

Despite the effect of different manufacturing parameters on the response of the polymers, the overall mechanical properties of PLA used in this research are listed in Table 1, based on literature [47-48]. Density, elastic module, yield strength, ultimate tensile strength, and Poisson ratio were obtained from technical datasheets provided by the material manufacturer. These properties are typically reported by the manufacturer based on

standard testing methods [47]. Though, endurance limit, which refers to the maximum stress amplitude that a material can withstand for a given number of cycles without failure, cannot be obtained from technical datasheets and requires experimental testing. The endurance limit used in this study [48] was experimental obtained. It should be noted that YouSu brand polylactic acid (PLA) filament, was used in component fabrication for 3D printing. The properties of 3D printed parts can differ from those of the raw filament material. For example, while the density of PLA filament was typically reported as 1.24 g/cm^3 in manufacturer datasheets based on the ASTM D792 standard test method, 3D printed parts can have a different density due to their meta-structure. Additionally, the tensile module and yield strength of PLA filament are reported as 3.5 GPa and 60 MPa, respectively, based on ASTM D1238 standard test method. However, 3D printed parts may exhibit different values for these properties due to the complex interplay of factors such as infill percentage, layer height, printing speed, and other printing parameters. The endurance limit for 95% probability of survival and determined in terms of maximum stress and extrapolated at 2 million cycles to failure cycles, is considered equal to 0.1 final stress [48].

Table 1: Mechanical properties of 3D-printed PLA [47-48]

Characteristics	Unit	Amount
Density	gr/cm^3	1.252
elastic module	MPa	1280
Yield strength	MPa	70
Ultimate tensile strength	MPa	73
Poisson ratio	-	0.36
Endurance limit	MPa	7.3

The compressive and tensile behaviors of PLA are usually considered to be the same, but some studies have focused specifically on the compressive behavior of PLA. These studies aim to better understand how PLA responds to compressive forces and how it can be used in applications where compression is a dominant mode of loading, such as in bone tissue engineering and orthopedics [49-50].

2-2) Numerical Simulation

Structural optimization is an iterative technique that could help the refinement of designs. Then, a result of the proper-designed structural optimization could be a lightweight part, which has durable properties. An integrated computer-aided design (CAD) and computer-aided engineering (CAE) workflow by Dassault systems was proposed (Fig 1).

The use of additive manufacturing enables the fabrication of different patterns with varying mechanical properties. The filling parameters have a direct effect on the response of the metamaterials to external loadings. For instance, a recent study [51] on metal functionally graded gyroids employed three design methods (thickness graded, size graded, and uniform) to provide a map of the mechanical properties of stainless-steel scaffolds. In the proposed study, we selected five infill patterns, including honeycomb, rectangular, triangular, irregular octagon [51], and regular octagon [51] to investigate their effect on fatigue properties and mass, and the results suggest that further investigations on other patterns may be necessary, to compare the fatigue behavior. The infill patterns used in this study are shown in Fig 2.

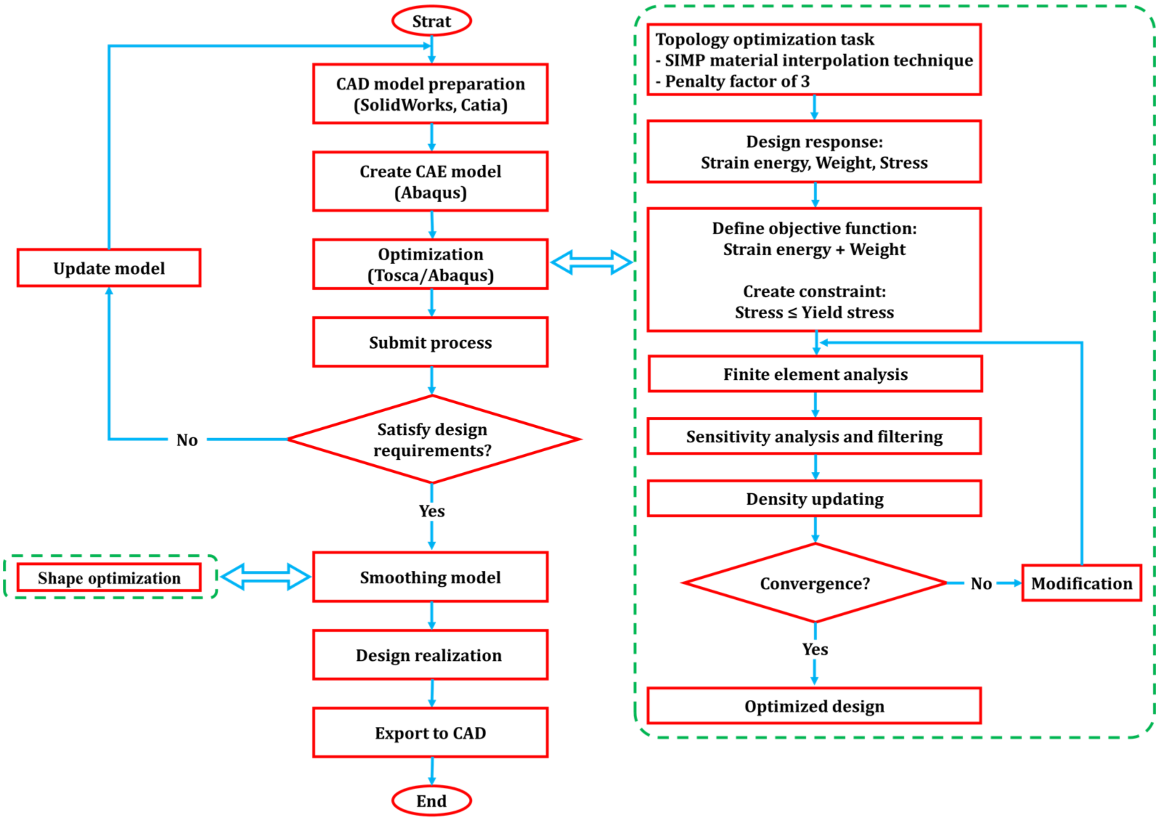

Fig 1: The workflow of topological designing using Dassault systems software

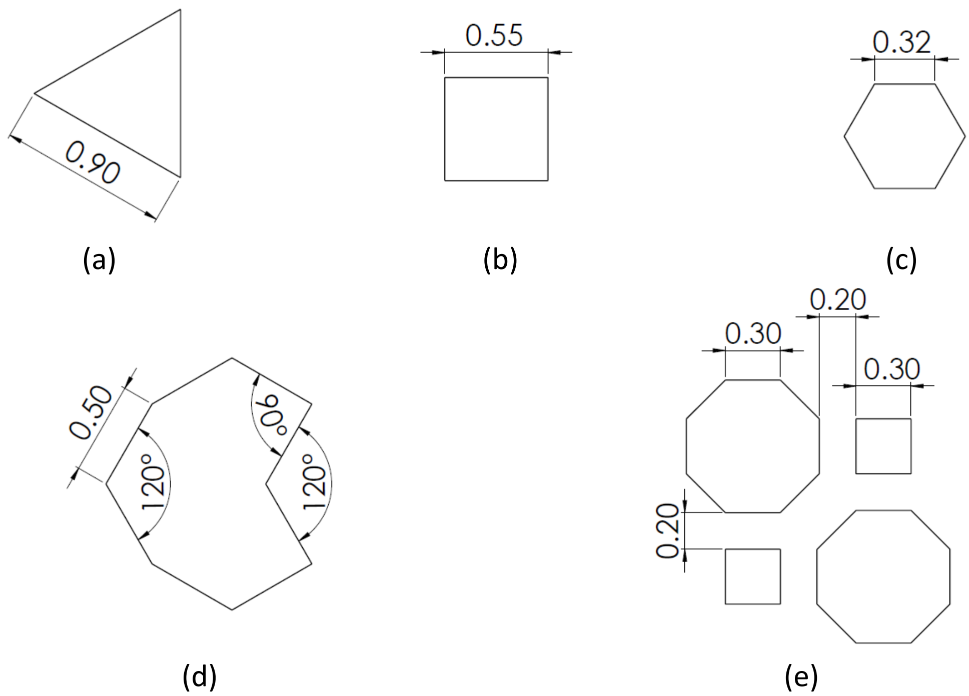

Fig 2: Different filling patterns: (a) triangular, (b) rectangular, (c) honeycomb, (d) irregular octagon, and (e) regular octagon

Based on the flow chart, a geometric model of the structure should be initially prepared in CAD software, including SolidWorks or Catia. Considering Table 2, filling densities of 40%, 60%, 80%, and 100% were also designed in the section of a cylinder with an outer diameter

of 9 mm and a height of 78 mm, by SolidWorks software. To achieve desired filling densities these geometries were arranged next to each other at different distances. In addition, two solid layers are considered as a perimeter with a width of 0.4 mm for each layer [52].

Table 2: The cross-section of structures for metamaterials

Fill pattern	40%	60%	80%
Honeycomb	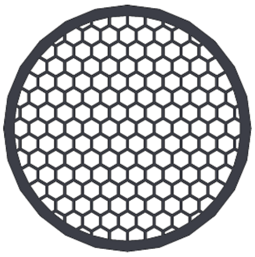	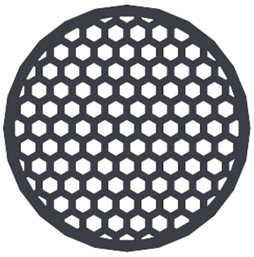	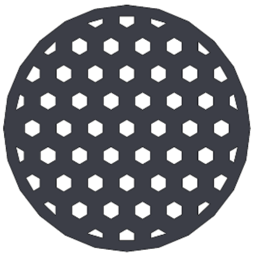
Rectangular	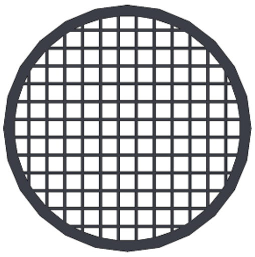	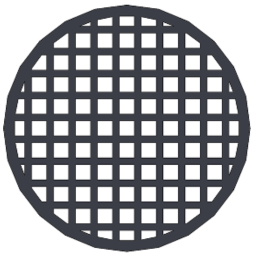	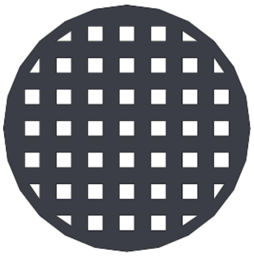
Triangular	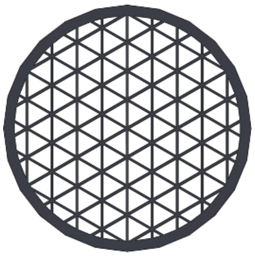	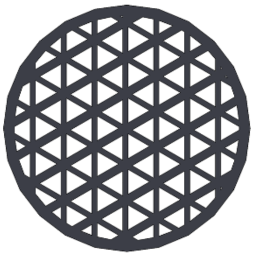	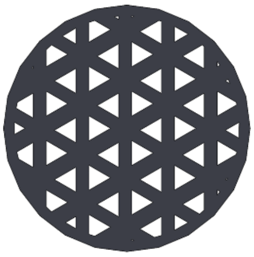
Regular octagon	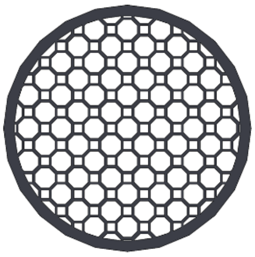	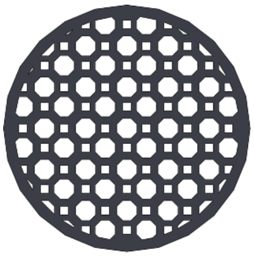	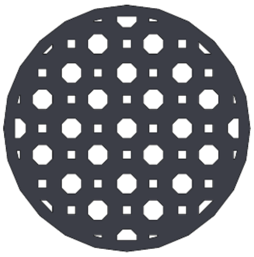
Irregular octagon	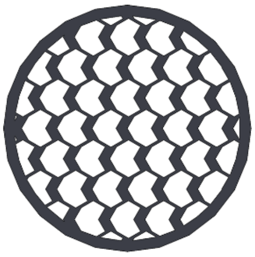	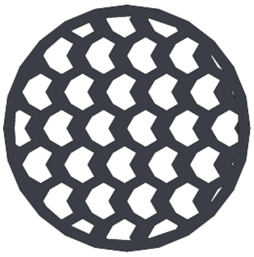	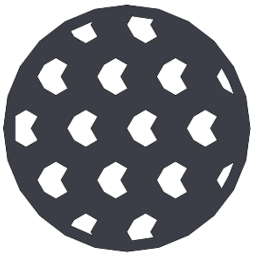
Full filled (100%)	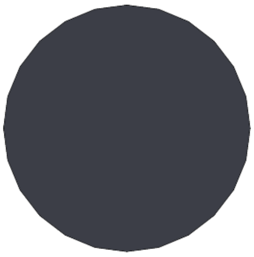		

A finite element model is fabricated in SIMULIA- ABAQUS for stress analysis besides the topology optimization. To calculate bending stress according to the rotating-bending fatigue testing method [53], the samples were subjected to a 10 N load at the free end of a cantilever which is clamped at the other end; therefore, the displacements of the supported end are constrained in three directions (Fig 3(a)). In the rotating-bending test, a common method used to evaluate the high cycle fatigue behavior of materials, the specimen is subjected to cyclic loading through rotational motion. During the rotating-bending test, the applied stress levels are typically kept below the yield strength of the material. This ensures that the deformation experienced by the specimen remains within the elastic range (<70 MPa [47]), where the material can fully recover its original shape after each cycle. The focus is on assessing the material performance to withstand cyclic loading without undergoing plastic deformation or failure. In this regard, Static, General simulations were performed in Abaqus/Standard. Regarding the mesh size, 8-node linear hexahedral solid elements with reduced integration (C3D8R) are considered with the global size of 0.7 mm to provide optimal accuracy and decrease CPU time and storage requirements (Fig 3(b)) [54]. Therefore, the number of elements meshed in each sample is listed in Table 3. There were some areas with thickness less than 0.7 mm. In such cases, to prevent the distorted elements in these thin areas, the minimum size control parameter (set to 0.1 of the global size) and the curvature control parameter (set with a maximum deviation factor of 0.1) adjusted the element size. Increasing the maximum deviation factor allows for a greater deviation between the actual geometry and the approximated geometry, and can be useful in ensuring mesh quality in thin regions. The minimum size control and curvature control parameters used in this study were recommended by the ABAQUS software algorithm.

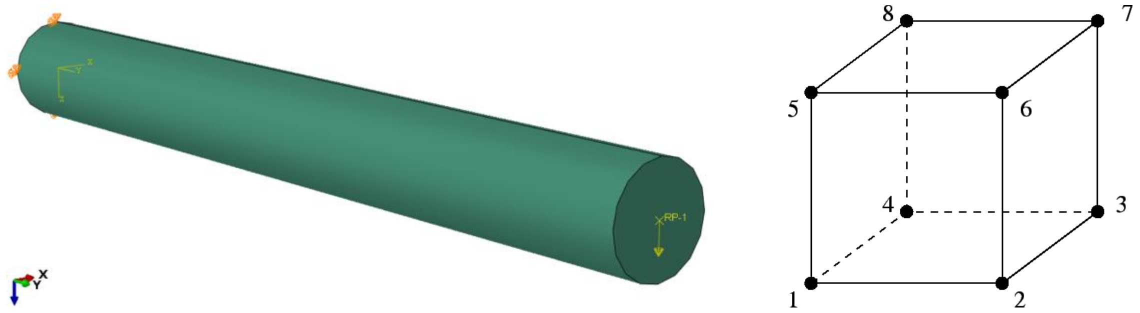

(a) (b)

Fig 3: (a) The applied load and boundary conditions and (b) the C3D8R element

Table 3: The number of elements in different models

Fill pattern / Infill density	40%	60%	80%	100%
Honeycomb	163947	93129	124653	
Rectangular	102897	51837	22644	
Triangular	149850	73704	90021	17538
Regular octagon	118690	13961397	131262	
Irregular octagon	101232	93936	26418	

2-3) Topology and Shape Optimization

Topology optimization is a proper method for designing that provides engineers with a powerful code in order to evaluate the solution space and enhance the designing reliability for any structures, in macro- and micro-scales. A wide range of various techniques exists such

as the solid isotropic material with penalization (SIMP) or the rational approximation of material properties (RAMP). Then, the tool carries out several iterations to find a consecutive answer by redesigning and simulating the structure. As the RAMP interpolation scheme is suitable for dynamic problems, the SIMP algorithm was used in this study.

For this topology optimization, the superior technique is the SIMP approach, which estimates an optimal distribution of the material under boundary conditions, loadings, manufacturing constraints, and any other requirements for the performance and properties.

The main technique in topology optimization is to discretize the solution domain into a grid of finite elements, which is entitled isotropic solid microstructures. Then, two events occur either the element is materially filled or the element becomes a void without a material (material-removing). This issue could be written as follows in mathematics by the density distribution of the material (ρ), which is a discrete binary value [54],

- $\rho_{(e)} = 1$ for material requiring,
- $\rho_{(e)} = 0$ for material removing.

Then, since the material density is a continuous value; therefore, the elastic module varies continuously in all elements, as the following equation [54],

$$E(\rho_e) = \rho_e^p E_0 \quad (1)$$

In this power law for each element (e), ρ_e is the relative density of the material, E_0 is the elastic module, and finally, p is a factor for the penalty which shows the contribution of elements with intermediate values of the density to the total stiffness. The literature illustrated that the superior value for the penalty factor is 3 [54].

As known, when the elastic module reduces, the element stiffness decreases. Based on the SIMP approach, the stiffness could be calculated as follows [54],

$$K_{SIMP}(\rho) = \sum_{e=1}^{N} \left[\rho_{min} + (1 - \rho_{min})\rho_e^p\right] K_e \quad (2)$$

In this formulation, ρ_{min} is the minimum relative density and K_e is the matrix for the element stiffness, where N is the element number. As a note, through the optimization iteration, a sensitivity analysis is done in order to find the influence of the density variation on the objective of the problem.

In the present study, the full-filled model was also used to find a topologically optimized structure. Then, the objectivities and the constraints were defined for the optimization in the topological design technique by simulation-based SIMULIA-Tosca, with a sensitivity-based optimizer to find an optimized trend for designing.

The optimization objective was to minimize the structure mass (the mass summation for all elements, ΣW_e) and also the strain energy (the compliance of a structure which is defined as the summation of the strain energy for all elements, $\Sigma u^t K u$. In this case, u is the displacement and K is the matrix to the global stiffness). This job was done under the stress constraint, which must be less or equal to 70 MPa which is the yield stress of polylactic acid (PLA). It should be noted that because of reduced relative density during the topology optimization, interpolation is required to calculate the stresses of elements, so the scaled element centroidal von-Mises stress of the design area is used in the optimization process [54],

$$Stress = \max \left| \frac{(\sigma_{vMises})^2}{\left(f(\rho_i)\sigma_y\right)^2} . \sigma_y \right| \quad (3)$$

where, σ_{vMises} is the element centroidal von-Mises stress, and ρ_i demonstrates a parameter for interpolating the stress in the element. This issue leads to a reduction in the current

relative density due to the topology optimization. Moreover, the reference stress, σ_y is given as follow [54],

$$\sigma_y = \min\left\{\alpha_1 \max\left|\frac{(\sigma_{vMises})^2}{\left(f(\rho_i)\sigma_{ref_1}\right)^2}.\sigma_{ref_1}\right| + \alpha_2 \max\left|\frac{(\sigma_{vMises})^2}{\left(f(\rho_i)\sigma_{ref_2}\right)^2}.\sigma_{ref_2}\right|\right\} \quad (4)$$

α_1 and α_2 are the weighting factors and σ_{ref} is the reference stress calculated during the initial optimization iteration [54].

As an important note, a mesh smoothing process was used by an additional shape optimization. This job was to have better quality for elements and also to minimize the value of von-Mises stress, as a minor modification. In other words, shape optimization was utilized in order to minimize the stress concentration in the finite element model, as a stress homogenization process [54].

Failing to properly realize the optimized design may result in a product that does not perform as intended or that is not feasible to manufacture, and therefore, it is crucial to carefully consider a design realization step in the topology optimization process to achieve a feasible component design concerning the manufacturing constraints. Therefore, for further post-processing development, some modifications were performed manually inspired by the geometry resulting from shape optimization, used to redesign a new part.

2-4) Fatigue Performance

To evaluate the fatigue lifetime, equation (5) was used [55]:

$$\sigma_a = \sigma_f'\left(2N_f\right)^b \quad (5)$$

in which b is the fatigue strength exponent, and σ_f' is the fatigue strength coefficient. Besides, σ_a is the stress amplitude which is equal to the maximum stress obtained from numerical simulation. Now the fatigue lifetime, N_f, can be calculated.

These parameters are material constants and differ notably when the printing conditions change. Nevertheless, b and σ_f' are provided in Table 4 for two print directions based on the previous studies by the authors [56]. In this table, fatigue tests were conducted at each stress level three times. Then, an S-N diagram was provided that fitted all the data. Moreover, the average of the results at each stress level was calculated and then again, another S-N diagram was provided. Although these material properties in Table 4 are not exactly as same as the material behavior of the fabricated samples in this work; they could be an initial prediction for the lifetime estimation. Besides, in this work, the infill density and the filling pattern were considered to study. These parameters could have effects on the stress value. This influence was also investigated by microscopic images, as to check the 3D printing quality.

Table 4: The parameters of the material including fatigue properties [56]

Material / Print direction		All data			Mean value		
		b	σ_f'	R^2	b	σ_f'	R^2
PLA	Horizontal	-0.2850	321.27	0.9680	-0.2880	334.29	0.9805
	Vertical	-0.2880	180.25	0.9573	-0.3010	207.90	0.9993

2-5) Two-Objective Optimization

A quantitative technique for solving two-objective optimization was used to find the optimized value of filling parameters. Therefore, using these optimization objectives leads to having a minimal mass and a maximal fatigue lifetime. In this case, three sets of objective

functions were considered. The first one uses the mass (M) and fatigue lifetime (N_f). The mass can be calculated with the production of material density and the volume of each specimen by fatigue lifetime is also obtained from equation 5. Mass reduction ratio and fatigue lifetime reduction ratio compared to the solid sample according to Table 5 are the second set of objective functions. The third set includes the distance from the optimal point (maximum mass loss and least lifetime reduction), D, and the sum of mass and fatigue lifetime, S.
In Table 5, M is mass and N_f is the fatigue lifetime. $\Delta N_f\%$ and $\Delta M\%$ are changed in fatigue lifetime and mass compared to the solid sample, respectively. $N_{f(solid)}$ and M_{solid} are the fatigue lifetime and mass of the solid sample, S is the sum of mass and fatigue lifetime changes, and D is the distance from the optimal point. Due to the decrease in mass compared to the solid sample, the mass change is considered negative.

Table 5: The combination of objective functions

Set	f_1	f_2
Set 1	max: $N_f = 0.5 \times \left(\frac{\sigma_a}{\sigma_f'}\right)^{\frac{1}{b}}$	min: $M = \rho V$
Set 2	min: $\Delta N_f\% = \frac{N_{f(solid)} - N_f}{N_{f(solid)}} \times 100\%$	min: $\Delta M\% = -\frac{M_{solid} - M}{M_{solid}} \times 100\%$
Set 3	min: $S = \Delta N_f\% + \Delta M\%$	min: $D = \sqrt{\left(\Delta N_f\%\right)^2 + (\Delta M\% + 100\%)^2}$

Considering different objectives in the Pareto front diagram, the performance-price ratio was used as an optimization reference. These objectives are similar to the fatigue lifetime and the mass (Set 3 in Table 5). Higher fatigue lifetime means better performance and lower mass means lower cost.
Accordingly, all stages of this analysis could be summarized based, as follows [57],
Step 1: Sorting the solutions in the diagram of the Pareto front by the objective (f_1) in ascending behavior [57].
Step 2: Considering the average line slopes for the average variability, based on objective functions (f_1 and f_2). The formulations for these slopes ($k_1^{(m)}$ and $k_2^{(m)}$) are as follows [57],

$$k_1^{(m)} = \frac{1}{2}\left(\frac{f_2^{(m)} - f_2^{(m-1)}}{f_1^{(m)} - f_1^{(m-1)}} + \frac{f_2^{(m+1)} - f_2^{(m)}}{f_1^{(m+1)} - f_1^{(m)}}\right), m = 2, 3, \ldots, M-1 \quad (6)$$

$$k_1^{(m)} = \frac{1}{2}\left(\frac{f_1^{(m)} - f_1^{(m-1)}}{f_2^{(m)} - f_2^{(m-1)}} + \frac{f_1^{(m+1)} - f_1^{(m)}}{f_2^{(m+1)} - f_2^{(m)}}\right), m = 2, 3, \ldots, M-1 \quad (7)$$

Let M denote the number of Pareto non-inferior solutions, and let x^m $(\mathrm{m} = 1,2,\ldots,\mathrm{M})$ denote the set of these solutions. In the Pareto front, the slope at endpoints ($m = 1$ and M) are $k_1^{(1)}$, $k_2^{(2)}$, $k_1^{(M)}$, and $k_2^{(M)}$, which could be proposed as follows [57],

$$k_1^{(1)} = \frac{f_2^{(2)} - f_2^{(1)}}{f_1^{(2)} - f_1^{(1)}} \quad (8)$$

$$k_2^{(1)} = \frac{f_1^{(2)} - f_1^{(1)}}{f_2^{(2)} - f_2^{(1)}} \quad (9)$$

$$k_1^{(M)} = \frac{f_2^{(M)} - f_2^{(M-1)}}{f_1^{(M)} - f_1^{(M-1)}} \quad (10)$$

$$k_2^{(M)} = \frac{f_1^{(M)} - f_1^{(M-1)}}{f_2^{(M)} - f_2^{(M-1)}} \quad (11)$$

Step 3: Performing the sensitivity analysis between two average variabilities in a specific non-inferior Pareto solution. For this purpose, the sensitivity ratio is defined as $\delta_1^{(m)}$, which is proposed by a ratio of $k_1^{(m)}$ to f_1. Then, $\delta_2^{(m)}$ could be also considered as the ratio of $k_2^{(m)}$ to f_2. These formulations could be found as follows [57],

$$\delta_1^{(m)} = \frac{k_1^{(m)}}{f_1^{(m)}}, m = 1, 2, \dots, M, f_1^{(m)} \neq 0 \quad (12)$$

$$\delta_2^{(m)} = \frac{k_2^{(m)}}{f_2^{(m)}}, m = 1, 2, \dots, M, f_2^{(m)} \neq 0 \quad (13)$$

Step 4: Nondimensionalizing the sensitivity ratio for a better comparison. Supposing ε_1^m and ε_2^m are the results of δ_1^m and δ_2^m, respectively, the non-dimensionalization leads to [57],

$$\varepsilon_1^{(m)} = \frac{\delta_1^{(m)}}{\sum_{i=1}^{M} \delta_1^{(i)}}, m = 1, 2, \dots, M \quad (14)$$

$$\varepsilon_2^{(m)} = \frac{\delta_2^{(m)}}{\sum_{i=1}^{M} \delta_2^{(i)}}, m = 1, 2, \dots, M \quad (15)$$

Step 5: Considering two values for the solution ($x^{(i)}$ and $x^{(j)}$) in the Pareto front diagram, if $\varepsilon_l^{(i)} > \varepsilon_l^{(j)}$ $(l = 1, 2)$; then, $x^{(i)}$ dominates $x^{(j)}$ and $x^{(i)} > x^{(j)}$; else, $x^{(i)}$ becomes the non-dominant solution or non-inferior solution in the sensitivity analysis [57].

Step 6: Considering a bias degree for various objectives between the value of 0 and 1. Then, For M^* as the solution number in the domain of X^*, w_1^m and w_2^m are defined as [57],

$$w_1^m = \frac{\varepsilon_1^{(m)}}{\varepsilon_1^{(m)} + \varepsilon_2^{(m)}}, m \in M^* \quad (16)$$

$$w_2^m = \frac{\varepsilon_2^{(m)}}{\varepsilon_1^{(m)} + \varepsilon_2^{(m)}}, m \in M^* \quad (17)$$

where,

$$w_1^m + w_2^m = 1, m \in M^* \quad (18)$$

For objective functions of f_1 and f_2, $(w_1)_{max}$ and $(w_2)_{max}$ have a strong value of the bias degree [57].

Step 7: Using Formulation (19), $\Delta\varepsilon^{(m)}$ (m = 1, 2, …, M) could be considered as [57],

$$\Delta\varepsilon^{(m)} = \left|\varepsilon_1^{(m)} - \varepsilon_2^{(m)}\right|, m = 1, 2, \dots, M \quad (19)$$

Step 8: Lowering $\Delta\varepsilon^{(m)}$ to obtain $(\Delta\varepsilon)_{min}$, which is the best unbiased solution. Notably, in this condition, the values of f_1 and f_2 are acceptable [57].

$$(\Delta\varepsilon)_{min} = \min\{\Delta\varepsilon^{(1)}, \Delta\varepsilon^{(2)}, \dots, \Delta\varepsilon^{(M)}\} \quad (20)$$

Step 9: End.

2-6) 3D Printing

Printing some regions of a part before starting the main study is a method of optimally manufacturing specimens, thereby reducing the financial and time loss caused by the trial-and-error stage. The selected patterns, including honeycomb, rectangular, triangular, regular octagon, and irregular octagon with 60% infill were printed along with rectangular patterns

with 40% and 80% of filling densities were printed as shown in Fig 4. The green lines in Fig 4 represent the desired part of the print samples that was cut and evaluated through inspection in the FESEM.

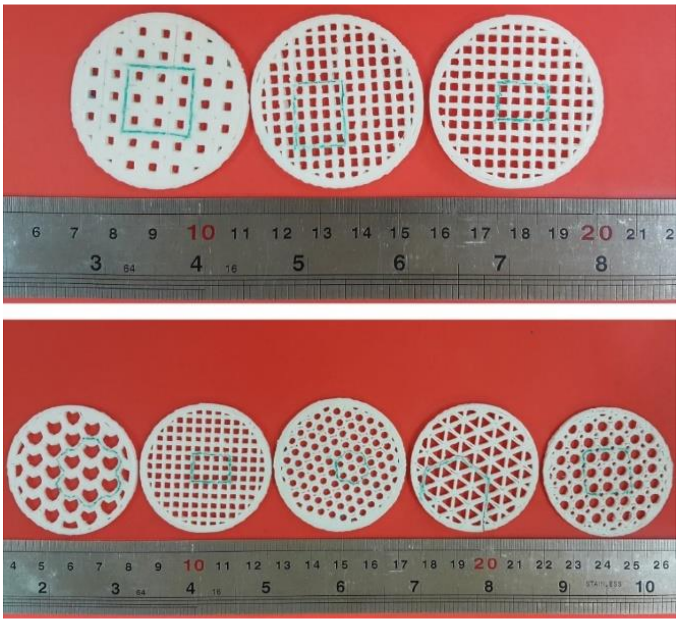

Fig 4: The geometry of the 3D-printed specimens with different filling patterns and infill densities

In order to provide the specimens, an FDM 3D printer was developed in the research laboratory of advanced materials behavior (AMB), as shown in Fig 5. Table 6 shows the conditions under which all of the samples were printed. To ensure optimal results and utilize the capabilities of an industrial-duty 3D-printer machine, the samples were printed at a scale of 5 times larger than the original CAD models. One other reason is due to the machine limitations. This approach allowed to produce lattice structures with circular sections and a diameter of 45 millimeters, which would have been challenging to achieve with smaller scales. While this approach allowed for the successful printing of the specimens, it is important to investigate the potential effects of scaling on the accuracy of the geometrical features and the resulting mechanical properties in future investigations. Previous studies demonstrated clear size effects on the mechanical response of additively manufactured lattice structures [58]. 3D printing was done with white-colored PLA filaments, which diameter was 1.75 mm, by the YouSu Company. The samples were printed with 100% infill and no top and bottom solid layers printed, allowing for clear visualization of the print path.

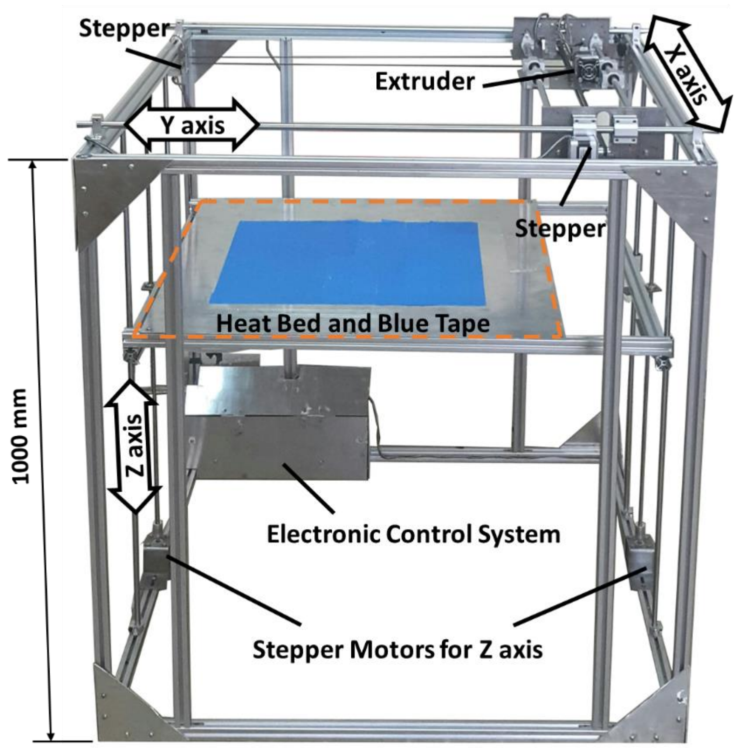

Fig 5: The FDM 3D printer used for additive manufacturing of the specimens

Table 6: The parameters of 3D printing [56, 59- 60]

Process variable	Operating condition
Nozzle diameter (mm)	0.40
Filament diameter (mm)	1.75
Layer height (mm)	0.20
Perimeter	2
Top and bottom solid layers	0
Print speed (mm/s)	10
Travel speed (mm/s)	30
Nozzle temperature (°C)	210 (First layer)<br>180 (Entire layers)
Bed temperature (°C)	25 (Room temperature)

2-7) Inspections

A close examination of the samples was performed after printing. For this purpose, a conductive golden layer was applied on the samples by gold sputtering and inspected using a TESCAN (MIRA 3 LMU) ~~FESEM~~. Imaging was performed with magnifications of different magnitudes. The length of the vertices and width of lines were measured in the microscopic images to understand the accuracy of prints.

3) Results and Discussion

3-1) Stress Analysis

Fill pattern and filling density are critical conditions for the fabrication of AM components. To investigate the influence of these parameters on the performance of printed parts, specimens

with different filling patterns and densities were modeled in ABAQUS software. Due to the great dependence of the stress analysis results on boundary conditions and loadings, especially the maximum stress, the constraints must be carefully set. Based on the results from stress analysis, the maximum stress occurs in the clamped region. As expected, there is a tensile state of axial stress at the top of the beam and a compressive axial stress state at the bottom. Thin walls between the patterns cause stress concentration, so the lower filling density cause higher stress. But the filling pattern affects differently in each filling percentage. As shown in Fig 6(a), honeycomb samples had higher stress in 40% and 80% infill, whereas in the case of irregular octagons, maximum stress occurred in 60% infill. However, irregular octagon, rectangular and regular octagon had the lowest stresses in 40%, 60%, and 80% of infill, respectively.

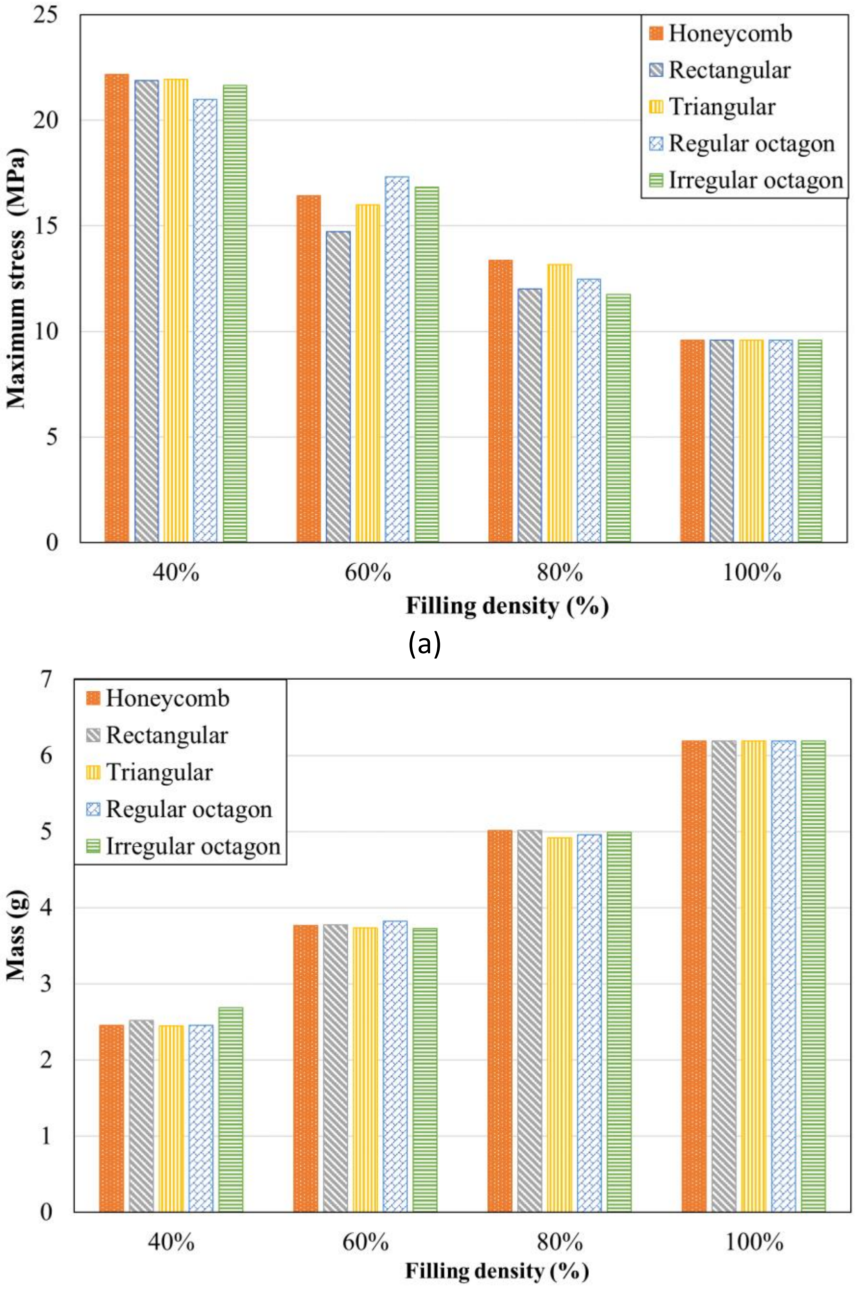

Fig 6: The influence of filling parameters on (a) maximum bending stress and (b) mass

The comparison of weight reduction in this study is conducted by considering the calculated weight values, taking into account the theoretical density of the material and the measured volumes of the samples in the CAD model. By utilizing these parameters, the weight of the objects can be estimated. Based on the results shown in Fig 6(b), the mass of the honeycomb and rectangular lattice structures was nearly identical at various infill densities, with the rectangular pattern being slightly heavier at 40% infill. In addition, the triangular pattern exhibited less mass compared to the other three patterns. The irregular octagon, regular octagon, and rectangular patterns were the heaviest at 40%, 60%, and 80% infills, respectively. However, it is clear that by decreasing filling density, specimens get lighter, and the triangular pattern has the lowest mass in all cases. The stress distribution contours obtained from finite element simulation are summarized in Table 7 and 8. As shown in stress distribution contours, the bending load generates compressive stress on the bottom side of the clamped end and tensile stress on its top side. However, in fatigue testing, the load is cyclic and changes the loading direction periodically, which leads to alternating regions of compressive and tensile stresses. Therefore, the specimens undergo both compressive and tensile stresses during fatigue testing, which can affect their durability and mechanical performance. Although the stress distribution in PLA specimens under cyclic loading cannot be measured experimentally, the fatigue behavior of the material is still worth investigating in future researches. This information can be compared to numerical results.

Table 7: The von-Mises stress distribution contours for different patterns

Pattern	40%	60%	80%
Honeycomb	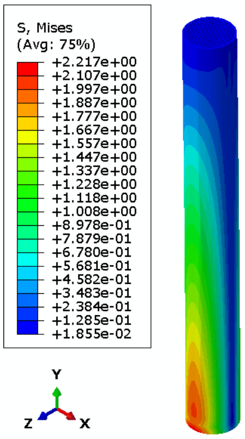	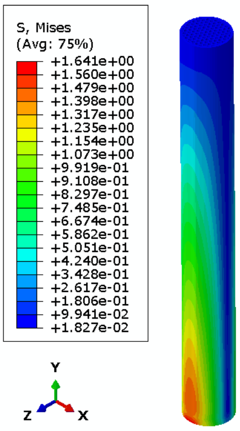	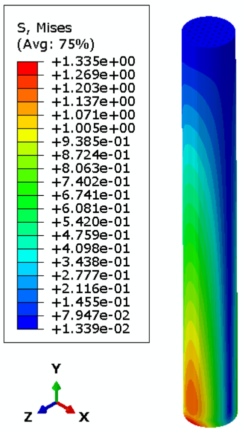
Rectangular	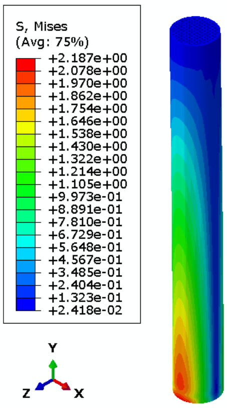	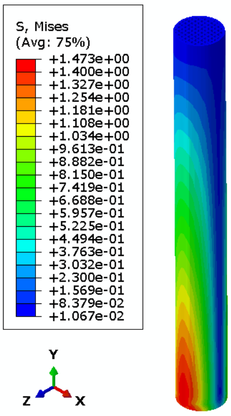	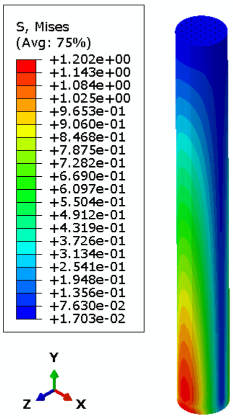

Triangular	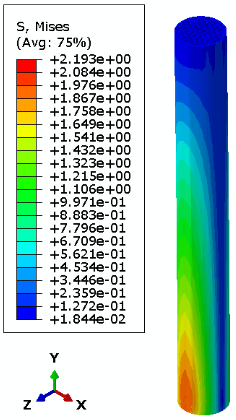	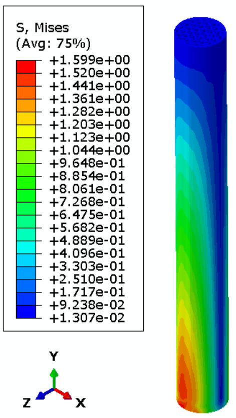	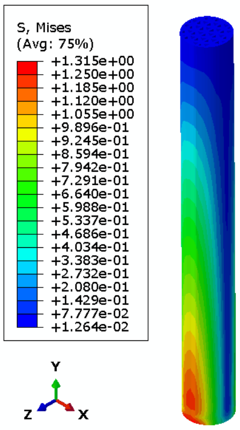
Regular octagon	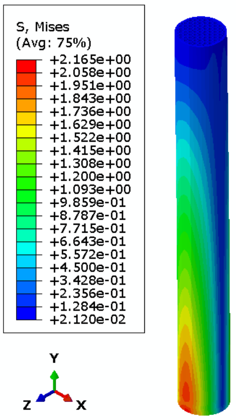	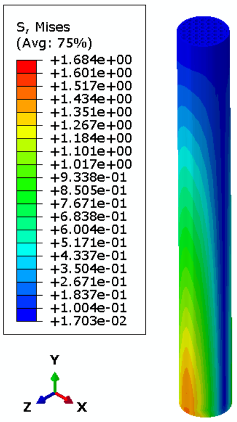	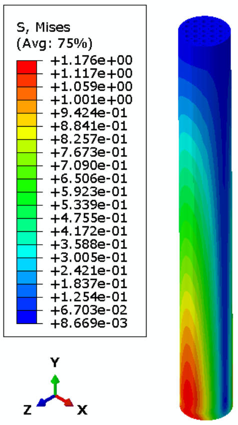
Irregular octagon	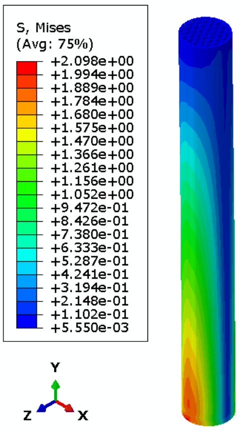	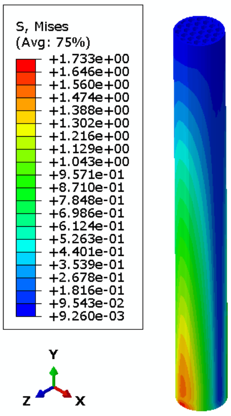	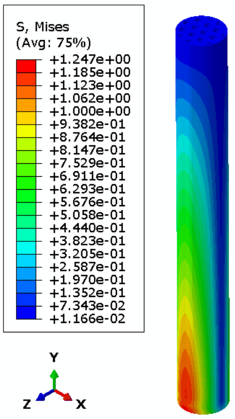
Full filled (100%)		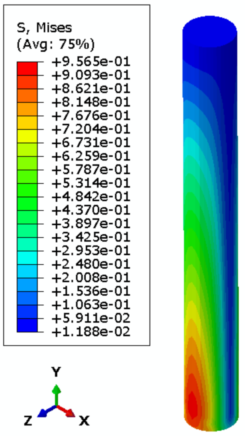	

Table 8: The stress distribution contours in Y direction (σ_{yy}) for different patterns

<table>
<tr><th>Pattern</th><th>40%</th><th>60%</th><th>80%</th></tr>
<tr><td>Honeycomb</td><td>
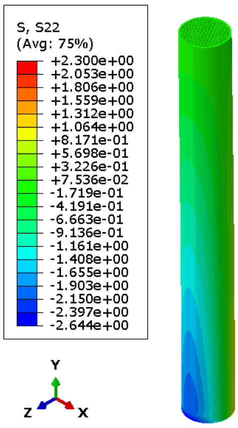

</td><td>
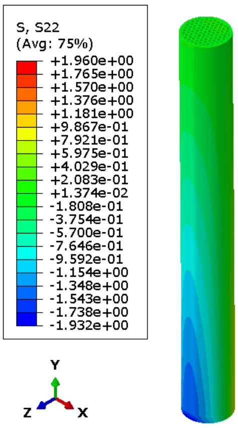

</td><td>
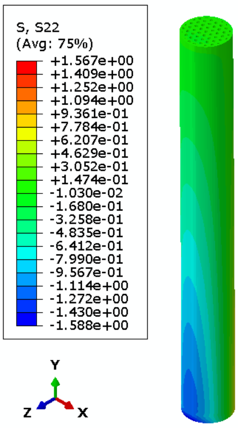

</td></tr>
<tr><td>Rectangular</td><td>
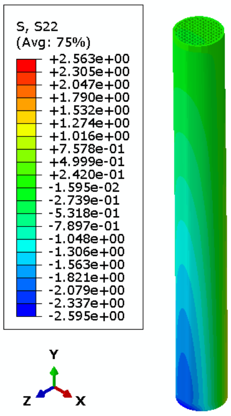

</td><td>
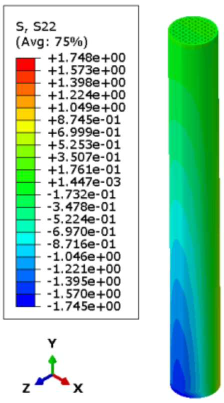

</td><td>
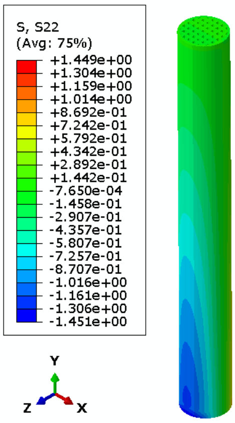

</td></tr>
<tr><td>Triangular</td><td>
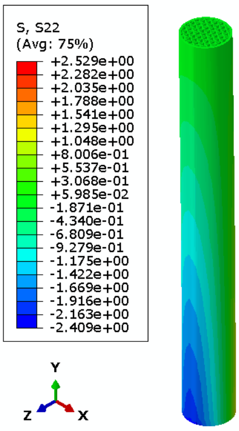

</td><td>
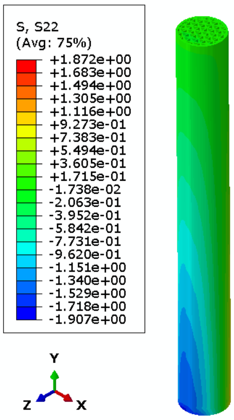

</td><td>
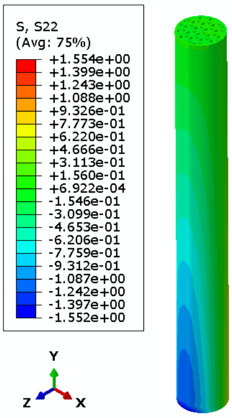

</td></tr>
<tr><td>Regular octagon</td><td>
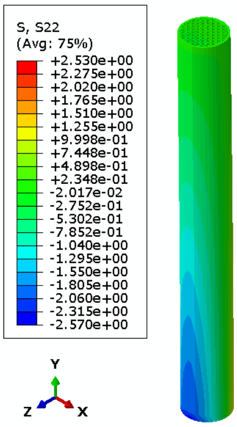

</td><td>
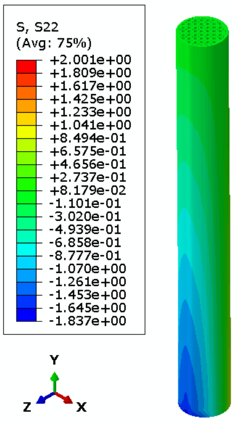

</td><td>
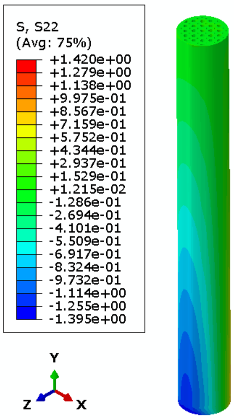

</td></tr>
</table>

<table>
<tr><td>Irregular octagon</td><td>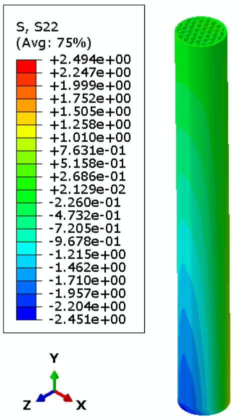</td><td>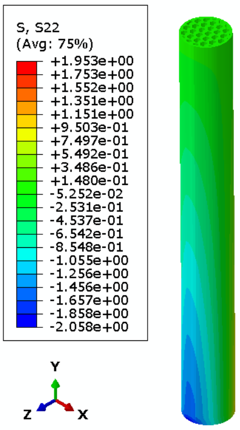</td><td>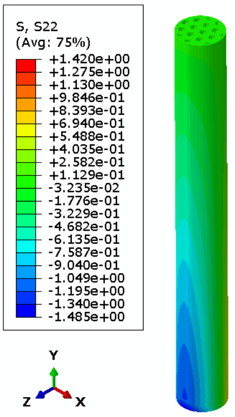</td></tr>
<tr><td>Full filled (100%)</td><td colspan="3">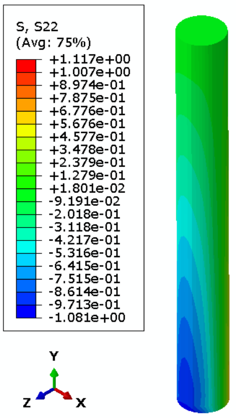</td></tr>
</table>

3-2) Two-Objective Optimization

To find the optimized filling parameters, a multi-objective optimization method proposed by Wang et al. [57], was used. First, based on the objective function proposed in Table 5, the distribution of the Pareto non-inferior solution set was obtained for each set. Using formulations (6)-(13), the sensitivity analysis on the non-inferior solutions of f_1 and f_2 was calculated. Then, based on Formulations (14) and (15), the sensitivity ratio of f_1 and f_2 was nondimensionalized. Formulations (16) and (17) were considered to find w_1 (for f_1) and w_2 (for f_2). Based on the dominance relationship of the bias degree of the 15 Pareto non-inferior solutions from each set of objective functions, eight solutions have been ruled out. The remaining solutions form the subset of non-inferior solutions based on the bias degree. Using Formulation (19), the values of Δε are obtained for each set of objective functions and presented in Tables 9 and 10 for horizontal and vertical print directions, respectively. The index of Δε in these tables indicates the corresponding objective function number. Therefore, $(\Delta\varepsilon)_1$, $(\Delta\varepsilon)_2$, and $(\Delta\varepsilon)_3$ are related to the first, second, and third set of objective functions. The minimum value of this parameter, $(\Delta\varepsilon)_{min}$, can be the superior unbiased solution, which could be acceptable for f_1 and f_2.

In the case of vertical print direction, the values of Δε are listed in Table 9. The solutions of 5th, 6th, 7th, 8th, 9th, 10th, 13th, and 15th from Set 1, 5th, 6th, 7th, 8th, 9th, 10th, 13th, and 14th from Set 2 and 4th, 5th, 6th, 9th, 10th, 11th, 14th, and 15th from Set 3 are dominated and therefore, they should be ruled out. Hence, the solution of 14th, 11th, and 13th are good results for vertical print direction and acceptable for both objectives in the first, second, and third set of objective functions, respectively. As it is obvious, by selecting different objective functions, the results will be different.

Table 9: Δε corresponding to Pareto non-inferior solutions for vertical print direction

No.	Filling density	Fill pattern	$(\Delta\varepsilon)_1$	$(\Delta\varepsilon)_2$	$(\Delta\varepsilon)_3$
1	40	Honeycomb	0.1095	0.0902	0.4380
2	40	Rectangular	0.3254	0.2972	0.4344
3	40	Triangular	0.3342	0.3029	0.4474
4	40	Irregular octagon	0.1166	0.1183	0.4177
5	40	Regular octagon	0.1085	0.0956	0.4645
6	60	Honeycomb	0.5889	0.1199	0.4561
7	60	Rectangular	0.6461	0.1046	2.3375
8	60	Triangular	0.4181	0.0665	2.3249
9	60	Irregular octagon	0.3591	0.0696	0.4842
10	60	Regular octagon	0.0293	0.0322	0.3795
11	80	Honeycomb	1.4354	0.6114	0.3521
12	80	Rectangular	1.3944	0.6027	0.0628
13	80	Triangular	0.0903	0.0394	0.0073
14	80	Irregular octagon	0.0735	0.0295	0.3310
15	80	Regular octagon	0.2226	0.0927	0.3872

Table 10: Δε corresponding to Pareto non-inferior solutions for horizontal print direction

No.	Filling density	Fill pattern	$(\Delta\varepsilon)_1$	$(\Delta\varepsilon)_2$	$(\Delta\varepsilon)_3$
1	40	Honeycomb	0.1092	0.0893	0.0907
2	40	Rectangular	0.3264	0.2948	0.0461
3	40	Triangular	0.3353	0.3000	0.0281
4	40	Irregular octagon	0.1152	0.1178	0.0469
5	40	Regular octagon	0.1066	0.0940	0.0668
6	60	Honeycomb	0.5302	0.1176	0.0129
7	60	Rectangular	0.5815	0.1004	0.1055
8	60	Triangular	0.3729	0.0633	0.0934
9	60	Irregular octagon	0.3198	0.0670	0.0116
10	60	Regular octagon	0.0265	0.0328	0.0980
11	80	Honeycomb	1.3343	0.6003	0.0484
12	80	Rectangular	1.2958	0.5945	0.0632
13	80	Triangular	0.0839	0.0400	0.0390
14	80	Irregular octagon	0.0691	0.0289	0.0497
15	80	Regular octagon	0.2083	0.0932	0.0756

On the other hand, for longitudinal print direction, according to the data listed in Table 10, the 1st, 5th, 6th, 7th, 8th, 9th, 10th, and 13th solutions from Set 1 are dominated. The 1st, 6th, 7th, 8th, 9th, 10th, 13th, and 14th solutions should be ruled out from Set 2 based on the dominance relationship. The 1st, 2nd, 5th, 7th, 8th, 11th, and 15th solutions are also the remaining solutions from Set 3. Therefore, the 14th, 15th, and 2nd solutions meet equation 20 and are determined to be the unbiased and good solutions in the case of horizontal print direction for Sets 1, 2, and 3, respectively. Notably, the quantized values of the bias degree for objectives are also calculated.

The decision can be balanced and selected more efficiently according to the quantitative indicator. For example, if objective f_1 is preferred in the third set of objectives, the 7th non-inferior solution should be selected as the bias degree of the solution, which reached 0.8112

for f_1 and it is only 0.1888 for f_2. Moreover, if the objective of f_2 is preferred, the 1st non-inferior solution is selected as the bias degree of the solution reaches 0.7742 for f_2 and it is only 0.2258 for f_1.
In Fig 7, the Pareto front indicates that there was not much change in the fatigue lifetime of vertical specimens (green points) even with a severe reduction in mass. On the other hand, the horizontal orientation (blue points) resulted in layer lines being placed horizontally in the direction of the bending force, making it the optimal orientation for mechanical strength. Despite the need for support during printing, this orientation provides the best mechanical strength. This result is also obtained in the literature [56,61], so horizontal print direction is selected in this study.

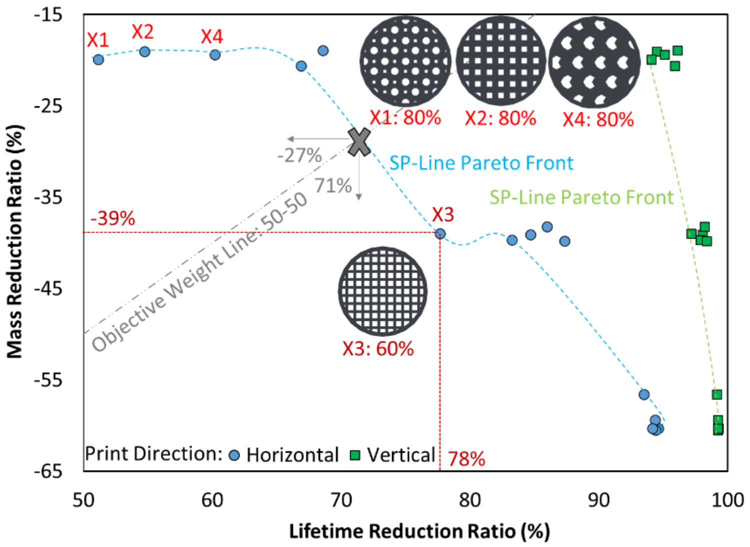

Fig 7: The Pareto front for filling parameters

In Fig 7, the point at which the 50-50 line crosses the Pareto front indicates a state of a 50% mass reduction and a 50% increase in the lifetime compared to the solid sample. According to the importance of mass in biomechanical applications, the rectangular pattern with 60% filling can be a good choice among the two samples that are closest to the point where the 50-50 line meets the Pareto front. Considering the interaction of objective functions, in the rectangular pattern with 60% of the filling density, the fatigue life has decreased by only 22%, despite the 39% reduction in mass. In addition, this sample has the shortest distance to the optimal point in the results of two-objective optimization by selecting the third set of objective functions (in Table 5) with an emphasis on the second objective. Based on this, fatigue samples should be printed with a 60% rectangular pattern.
The obtained results in the literature have also illustrated that the infill pattern through 3D printing influenced the mechanical responses of AM structures. For example, the triangular infill pattern with 40% infill density in PLA achieved a maximum peak force in low-velocity impact testing compared to the quarter cubic, grid, and tri-hexagon patterns [62]. The optimal filling pattern for the nylon matrix with a reinforcement under the uniaxial tension-tension fatigue test was triangular, with 20% of the filling percentage [63]. Moreover, the triangular shape had a superior tensile strength in the composites, reinforced with continuous carbon fibers and chopped carbon fibers (Onyx) [64]. The rectilinear pattern presented the best mechanical performance, having a high elastic module, tensile strength, and impact resistance, compared to the grid, honeycomb, and triangle [65]. The specimens manufactured

through a filling pattern of honeycomb with 75% of the filling density could be preferred to the rectilinear samples to increase the fatigue lifetime of PLA-wood composite structures [52].

Vigneshwaran and Venkateshwaran [66] found that changing the filling percentage (in naturally available wood flour reinforced with 30-90% of biodegradable PLA) multiplied the tensile performance and also the stiffness by factors of 2.0 and 1.5, respectively for wood-PLA bio-composites. According to their results, the printing pattern, whether hexagonal, rectangular, or triangular did not find to affect the final characteristics of bio-composites. The rectangular-filled nylon structure illustrated the maximum value for tensile properties compared to triangular- and hexagonal-filled components. On the other hand, the triangular-filled part had the minimum value of tensile properties, while the hexagonal-filled sample demonstrated a lower value for tensile performance than the rectangular-filled part [67]. The highest value of strength and lowest value of deformation are related to the irregular octagon structure and under 10 N compression load compared to the regular octagon under 10 N compression load [51]. As a result, depending on the material and the conditions of use, the performance of filling parameters is different and should be considered in the design domain.

3-3) Numerical Results and Optimization

The topology optimization procedure was done in ABAQUS with the SIMP technique. Fig 8 shows the resulting plot of this process. According to this figure, a mass of 1.1767 g and a total strain energy of 0.056 J were achieved after 50 cycles. As a result of the optimization efforts, the desired optimized geometry was obtained. A threshold of the ISO value is generally used for regulating, which the finite elements are displayed. This value is utilized to obtain the positions on the finite element edges, where the new nodes are made and must be between zero and unity. Larger values lead to a model with a smaller volume. The determined design is depicted in Fig 9. Notably, it should emphasize that the proposed geometry only demonstrates the elements with a density higher than 20% to maintain the continuity of components.

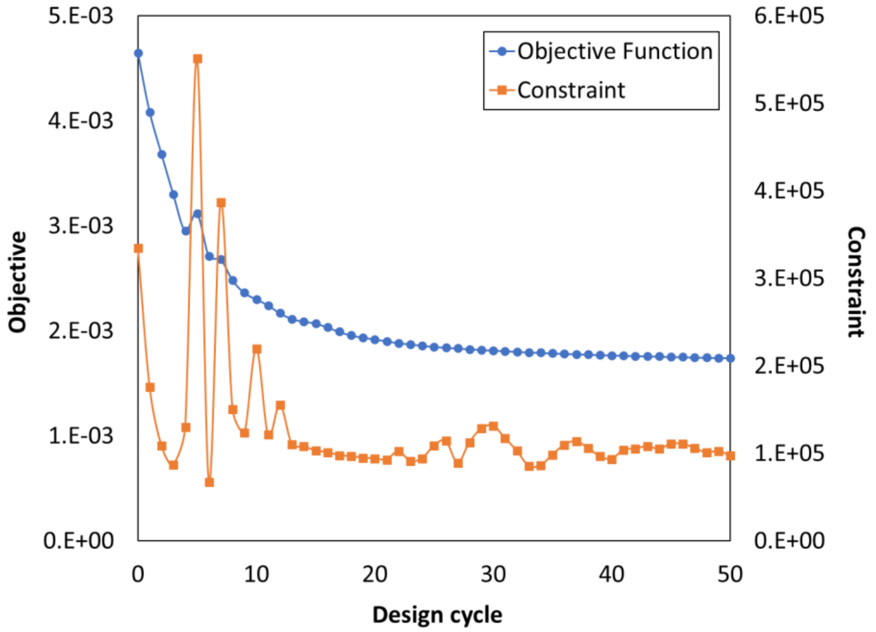

Fig 8: The variations of design responses through design cycles

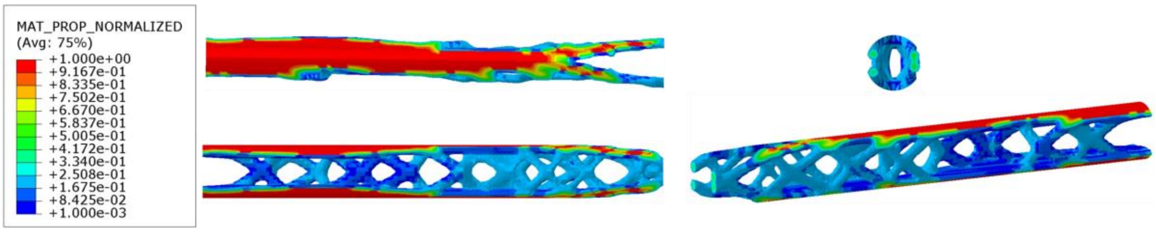

Fig 9: The extracted geometry from topology optimization

The optimized geometry in Fig 9 was reached after 50 design cycles. An animation is also included in supporting information (Fig S1) that shows the topology optimization process developments during each design cycle, providing a visual representation of the design process. Such visual support presents a better understanding of the design evolution and the optimization steps taken to arrive at the final design. The optimized geometry has a unique characteristic in its design - a quadruple thread-like pattern surrounded by a shell. This pattern is evident throughout the structure and adds a distinct visual aspect to the overall design. The quadruple thread-like pattern with its shell provides an optimized structure with improved mechanical performance by reducing mass and enhancing strength in specific locations. The literature supports that the outer shell affects bending strength. Samples with ABS: TPU: ABS sandwiched structure showed higher flexural strength due to ABS material in outer layers, aiding compression and expansion during loading [5]. The thread-like pattern serves as a unique solution in topology optimization and can be customized to meet specific design requirements. Accordingly, the design realization step was performed for further considerations of manufacturability. This could be a crucial step in topology optimization since it is the stage where the optimized design is translated into a manufacturable and practical product. In this step, the abstract and optimized design obtained from the topology optimization process is transformed into a tangible, functional product that can be manufactured. The design realization step involves several important tasks, including geometry clean-up, and preparation of the final design for manufacturing. In this step, the design must be refined to ensure that it meets all design requirements and constraints and can be manufactured using the desired materials and processes. The realized design, which is presented in Fig 10, is believed to be conceptually feasible due to rotational symmetry. This design approach has potential applications in various industries, such as biomedical to produce stents, where reducing mass and increasing strength are critical considerations. The printed specimen in Fig 11 exhibits the desired geometry and structural features as intended in this design. This physical realization served as a tangible representation of the proposed model and validated the effectiveness of the present design process. It provided concrete evidence that the optimized design can be successfully translated into a physical object through 3D printing.

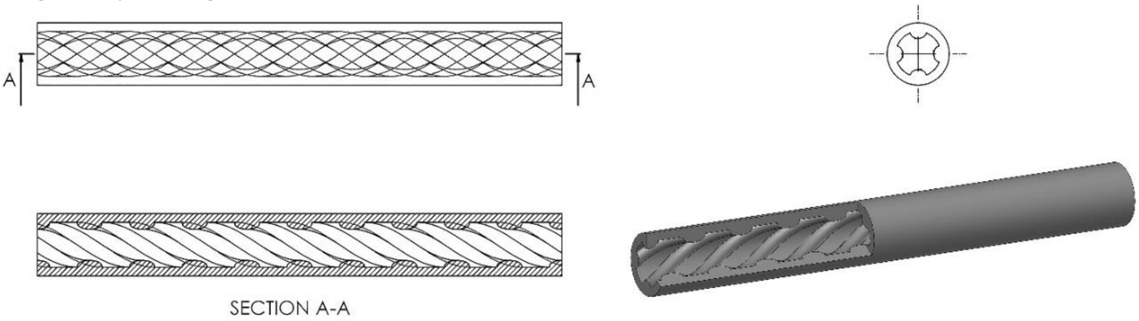

Fig 10: The geometry of the designed sample based on the result of topology optimization

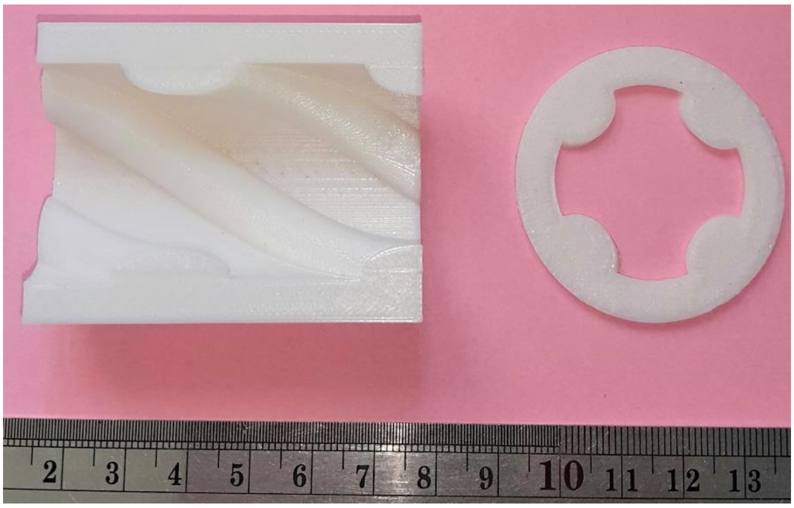

Fig 11: The designed sample printed based on the result of topology optimization

Fig 12 and 13 present the results of the static analysis of the optimized components. Fig 12 presents three geometries after (a) topology optimization, (b) shape optimization, and (c) design realization steps. The figure provides a visual depiction of how the optimization process affected the stress distribution of the component and how the optimized design was implemented in the final product. The maximum von-Mises stress was 97.08 MPa, which was observed after the topology optimization and located at the point shown in Fig 12(a). As it can be seen, the obtained geometry proposed several sharp edges and also some irregular surfaces. This issue is related to mesh refinement under boundary conditions.

Thus, a post process should be done based on smoothing and repairing the previous operations on such a sharp surface, by a shape optimization in order to minimize the von-Mises stress. As a result, maximum stress homogenized and decreased to 44.30 MPa after 15 design cycles (Fig 12(b)). The geometry after the design realization step is the final product of the optimization process. This geometry represents the optimized component that has been manufactured using 3D printing technology. The optimized component was designed based on the results of the multi-objective optimization, which took into consideration factors fatigue lifetime and mass. Despite the increase in mass compared to the topology optimized sample, the maximum von-Mises stress greatly reduced, based on Fig 12(c). With a mass of 3.56 g, this sample is lighter than the samples with 60% of filling density. However, due to less stress, it tolerates more cycles. The design realization step is the final step in the optimization process and the optimized component is now ready to be used in applications that require its unique properties. Figure 13 displays the stress distribution along the component axis in optimized geometries. The contour lines in the figure accurately represent the stress distribution, including stress concentration regions, mirroring the pattern observed in the von-Mises stress. Additionally, the figure distinctly reveals areas with compressive stresses.

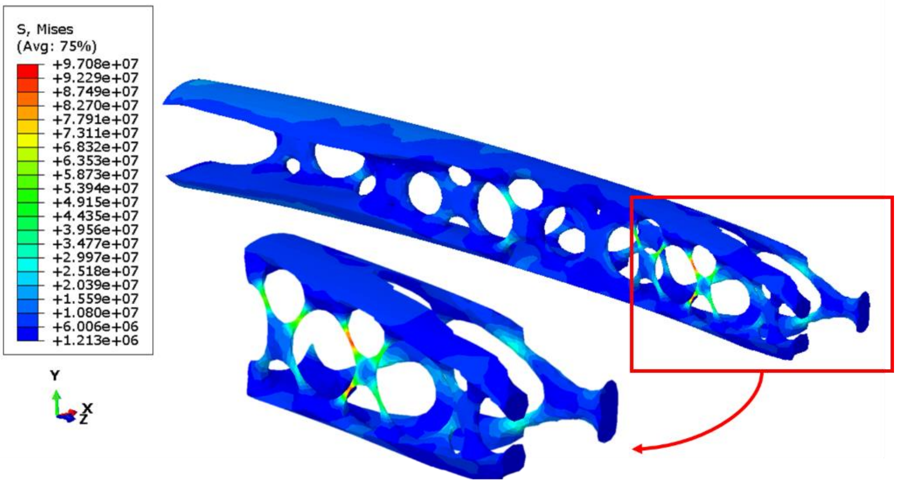

(a)

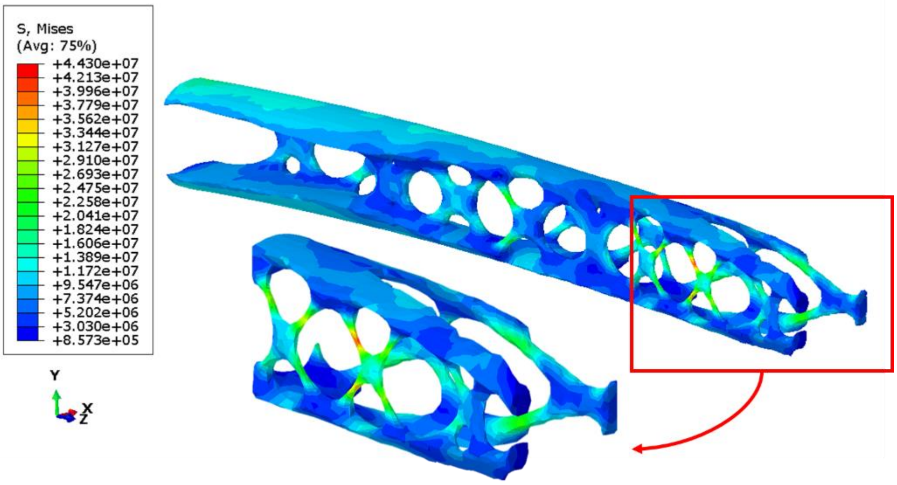

(b)

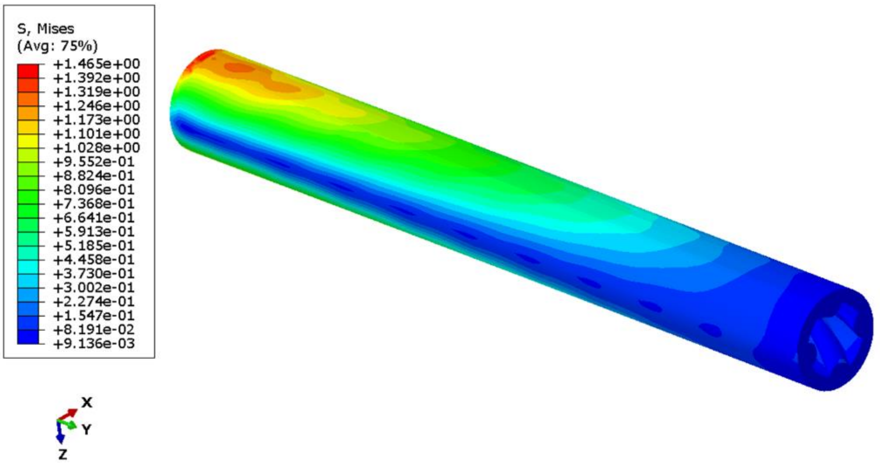

(c)

Fig 12: The von-Mises Stress distribution in the optimized component after (a) topology optimization and (b) shape optimization (c) design realization

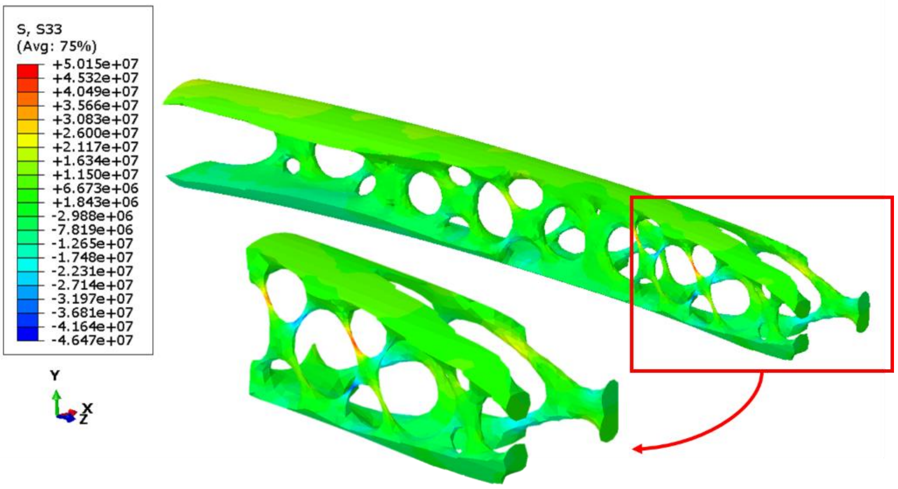

(a)

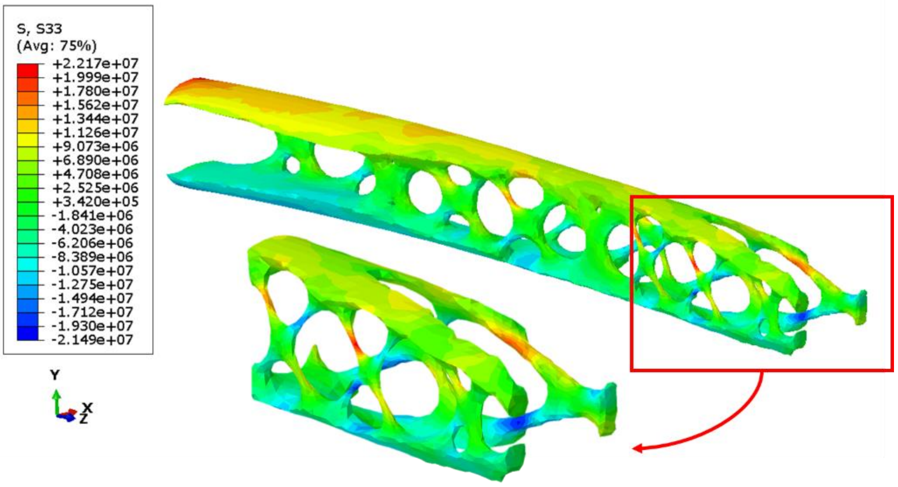

(b)

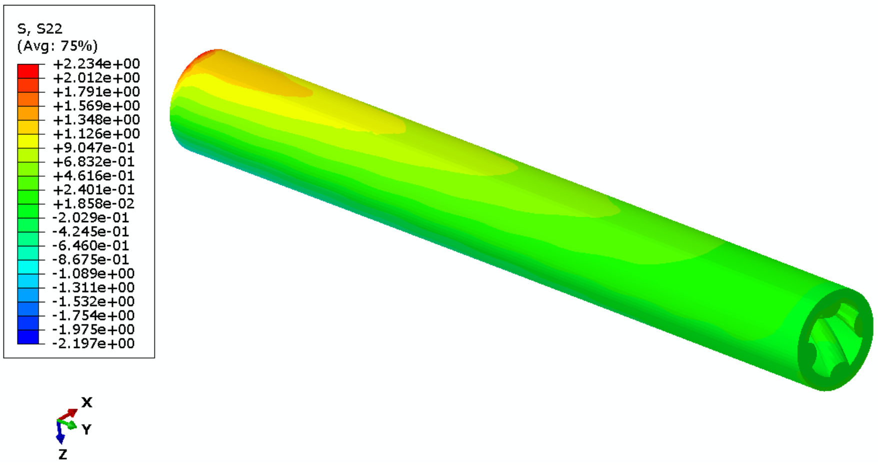

(c)

Fig 13: Stress distribution in the direction of cylinder axis in the optimized component after (a) topology optimization and (b) shape optimization (c) design realization

Table 11 compares the results obtained from the various optimization processes. This table provides a quantitative evaluation of the impact of the optimization process on the mass of components, maximum von-Mises stress and fatigue lifetime based on the printing direction. The comparison allows the assess the effectiveness of the optimization process and draw

conclusions about the optimal values. This information can be useful for engineers and designers in future 3D printing projects.

Table 11: The comparison of the results through the optimization processes

Optimization process	Mass (gr)	Maximum von-Mises stress (MPa)	Fatigue lifetime (cycle)	
			Horizontal	Vertical
Topology	1.18	97.08	37	6
Shape	1.18	44.30	564	85
Realized design	3.56	1.46	80424865	7050142

3-4) Morphological Characteristics

The desired part of the print samples, which are marked with green lines in Fig 4, has been cut, evaluated by inspection in the FESEM, and then examined for dimensional and geometrical accuracies. In order to assure adequate electrical conductivity for FESEM observation, samples were coated with a golden layer. The imaging process was performed with different magnifications. The printed samples had acceptable overall compliance of the shape with designed geometries, according to low magnification images (Fig 14). However, it is also obvious that the regularity of the rectangular pattern was the best, while the regular octagon pattern was the most irregular. Easy to be noticed by the eye, the line spacing is significant in the honeycomb pattern. In addition, with higher fill percentages, the spacing between lines decreases.
As illustrated in Fig 15, voids could be observed at the turning points of the extruder. According to previous studies, this void enhances by increasing the temperature of the extrusion. An incorrect control of the temperature through filament depositing can affect the adhesion between 3D-printed layers and also the filament sides along the filling pattern through the raster (Fig 16(a)) [68]. In other words, the air gap between the rasters enhances by increasing the deposition temperature. Notably, the raster width has a higher value at lower temperatures and therefore, it effectively reduces by enhancing the deposition temperature [69].

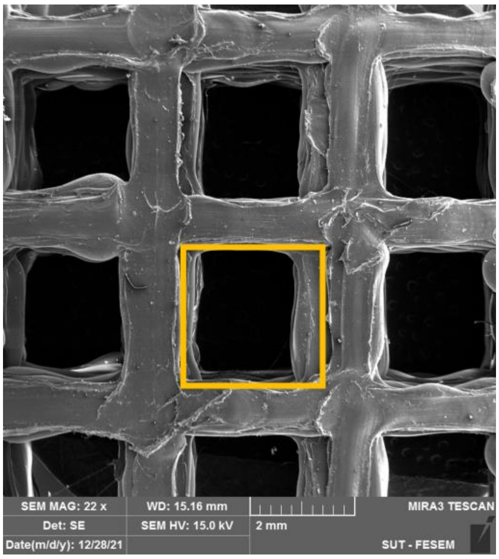

Rectangular (40%)

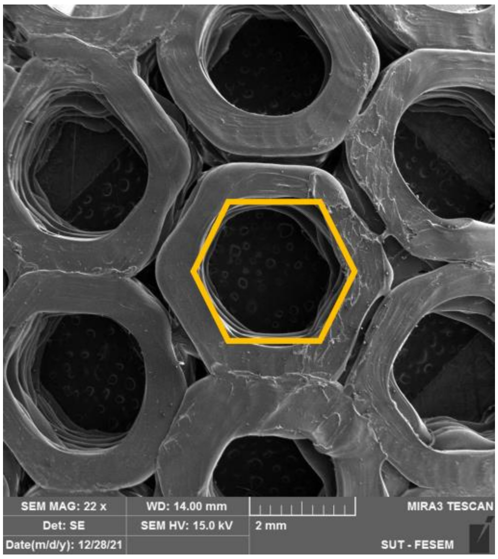

Honeycomb

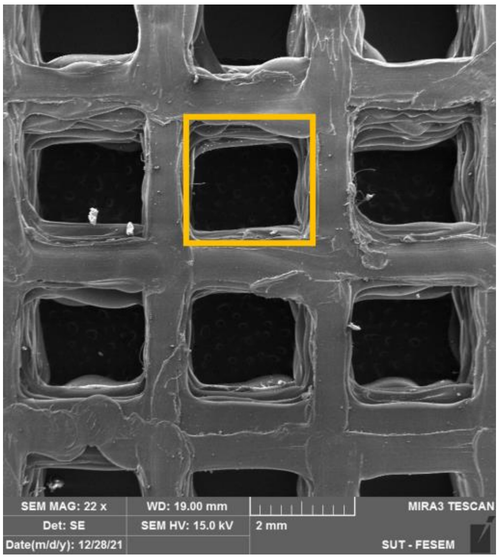

Rectangular (60%)

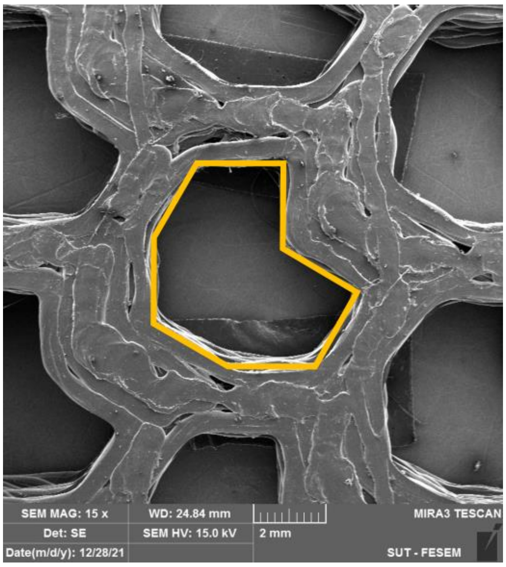

Irregular octagon

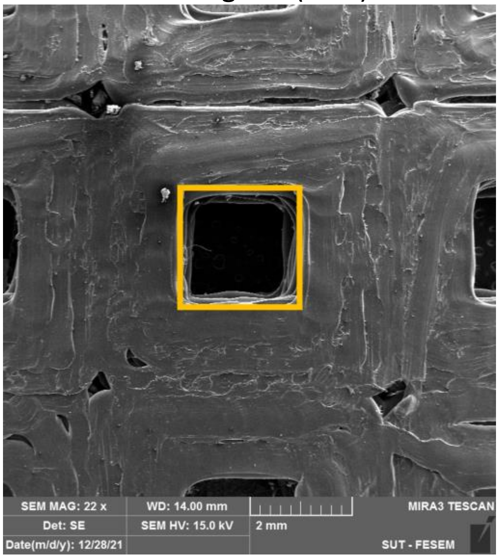

Rectangular (80%)

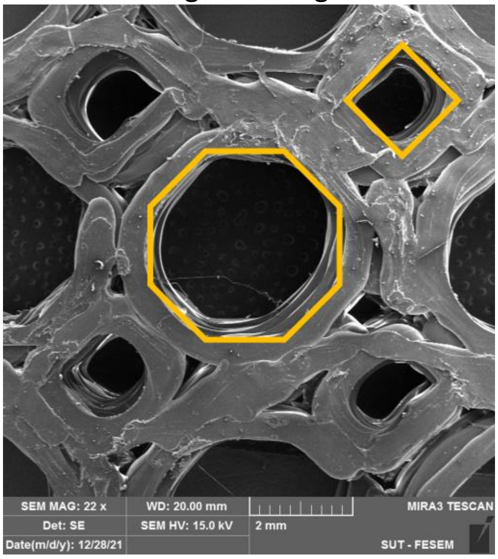

Regular octagon

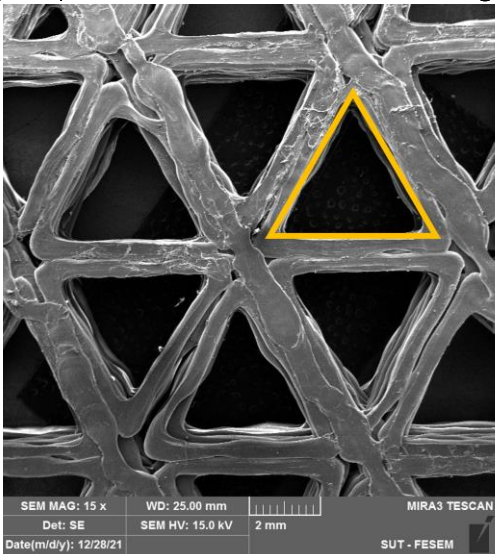

Triangular

Fig 14: The low-magnification FESEM images of the printed samples

As observed in Fig 16(b), some small spherical-shaped defects may be generated during the fabrication of the filament, which can negatively impact the mechanical properties. Additionally, the extrusion feed rate plays a critical role in determining the quality of printed parts, as shown in Fig 16(c). If the extrusion feed rate is not balanced with the extrusion temperature and printing speed, inconsistent fusion can occur, resulting in defects. Another parameter that can affect the quality of printed parts is the distance between the nozzle and the build platform [70]. This distance can impact the adhesion between the layers and can cause defects similar to those shown in Fig 16(c). It is essential to consider these factors during the design and optimization of 3D-printed parts to achieve the desired mechanical properties and avoid potential defects.

Based on the microscopic images, different kinds of voids can significantly impact the quality and properties of 3D-printed parts, and the infill patterns examined in this study is one such example [56]. The infill patterns, often referred to as meta-structures, play a crucial role in influencing the properties of the components. Additionally, there are other forms of anisotropy that can arise from changes in printing paths or inadequate print parameters. While these factors were not specifically investigated in this study, it was reported from the porosity analysis that the adhesion between the laminated layers has no significant role to play in flexural properties [6].

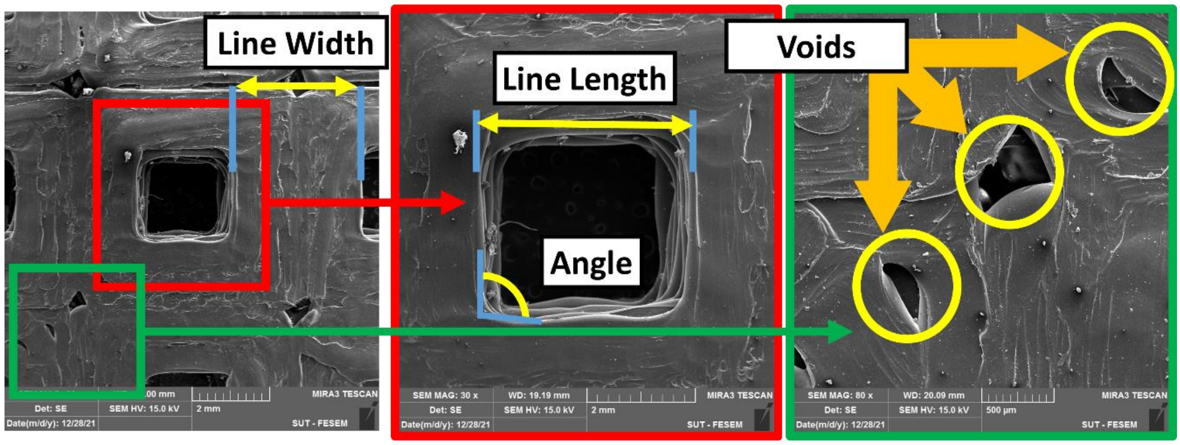

Fig 15: The FESEM micrographs of the 3D-printed PLA with rectangular pattern and 80% of infill at different resolutions

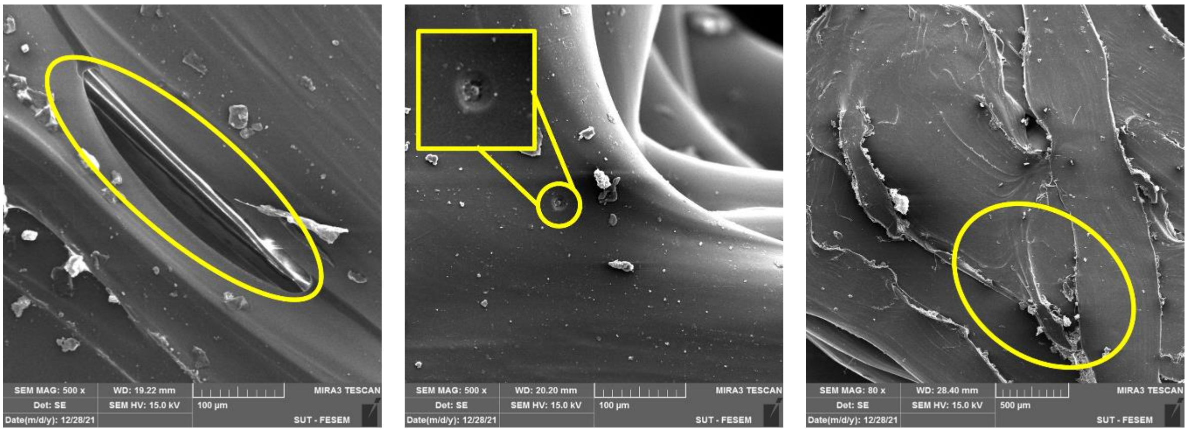

(a) (b) (c)

Fig 16: The defects observed in the samples with (a) 60% infill regular octagon, (b) 80% infill rectangular, and (c) 60% infill irregular octagon

As illustrated in Figs 15 and 17, the length of the vertices and width of lines were measured in the microscopic images to visualize dimensional accuracy. Specifically, Fig 15 shows a schematic of the measured line width and line length.

The measured line width in this picture does not refer to the actual line width produced by the nozzle. Rather, it is the width of the walls in the structure, which may consist of multiple print paths.

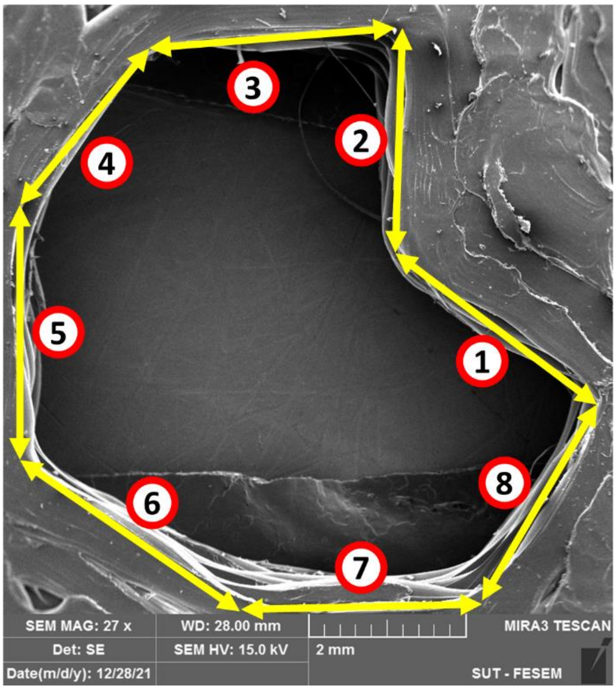

ID	Length (mm)	Angle (degree)
1	3.329	146.4
2	3.087	85.7
3	3.526	3.4
4	3.067	38.8
5	3.463	92.6
6	3.556	145.9
7	3.300	0.2
8	3.402	60.0

Fig 17: The dimensional accuracy of the irregular octagon, measured from FESEM micrographs of the 3D-printed PLA with 60% of infill

Fig 16 shows the measured values in an irregular octagon as a sample. The ImageJ software was used to measure the length and width of the lines, as well as the angle between them. The process repeated three times to ensure accuracy. The results were within a reasonable tolerance based on the difference between the demanded and measured values as well as the standard deviations according to Table 12. The comparison between the average values measured from the microscopic images and corresponding CAD models is done in this table. The purpose of this analysis was to assess how accurately the 3D printer was able to reproduce the intended geometry of the parts, and to identify any potential sources of error or variability in the printing process. By comparing the actual dimensions of the printed parts with the intended dimensions, it is possible to gain insight into the overall quality and reliability of the printing process, and to identify opportunities for improvement. A scatterplot was used to investigate the relationship between demanded and measured data (Fig 16). The rectangular sample with 40% of infill and the regular Octagon with 60% of infill was the worst parameters.

Table 12: The dimensional variations of samples printed as per the experimental design

Parameter / Fill pattern	Rectangular			Triangular	Honeycomb	Regular octagon	Irregular octagon
Demanded Infill (%)	40	60	80	60	60	60	60
Measured Infill (%)	74.61	77.44	90.27	69.7	75.55	70.24	71.66
Standard Deviation	86.53	29.07	12.84	16.17	25.92	17.07	19.43
Demanded Line Width (%)	2.75	2.75	2.75	4.50	1.25	1.50	2.50
Measured Line Width (%)	2.78	2.57	2.41	4.36	1.49	1.54	2.39
Standard Deviation	0.12	0.13	0.09	0.17	0.10	0.10	0.15
Demanded Line Length (mm)	0.50	1.25	3.00	1.00	1.60	1.25	3.3
Measured Line Length (mm)	0.83	1.18	3.41	1.02	1.57	1.46	3.39
Standard Deviation	0.10	0.14	0.13	0.05	0.11	0.22	0.09

Based on the scatterplot presented in Fig 18, it is evident that there exists a strong positive and linear relationship between the demanded and measured line widths (Fig 18(a)) as well as the demanded and measured line lengths (Fig 18(b)). This indicates that as the demanded line width or line length increases, there is a corresponding increase in the measured values, highlighting the consistent and proportional behavior between the two variables in the printing process. Dimension reduction compared to the designed value occurs as a result of excessive deposition of the material during the extrusion process (due to not considering the dwell time at the endpoints of raster paths) and transverse flow (due to the relationship between temperature and viscosity). This is while the increase in dimensions can be due to the shrinkage of the printed polymer [71]. When there is an inaccuracy in the nozzle position, an imperfect configuration will occur. However, this issue could be solved by the calibration of the 3D printing machine [68]. It seems that such inaccuracy increases with the decrease in filling percentage and therefore the thickness of the walls. The sum of these items results in differences in filling densities and should be considered in the design phase.

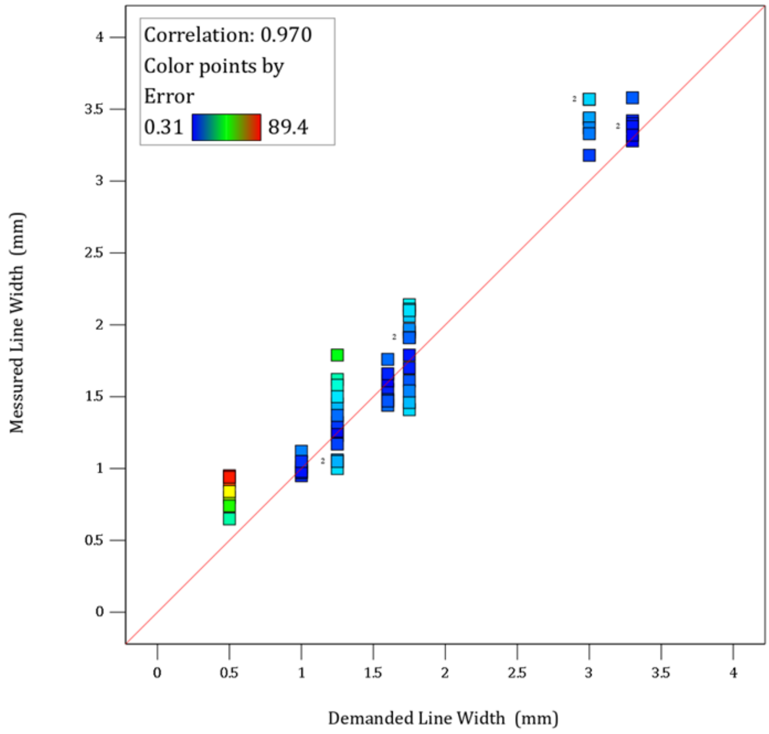

(a)

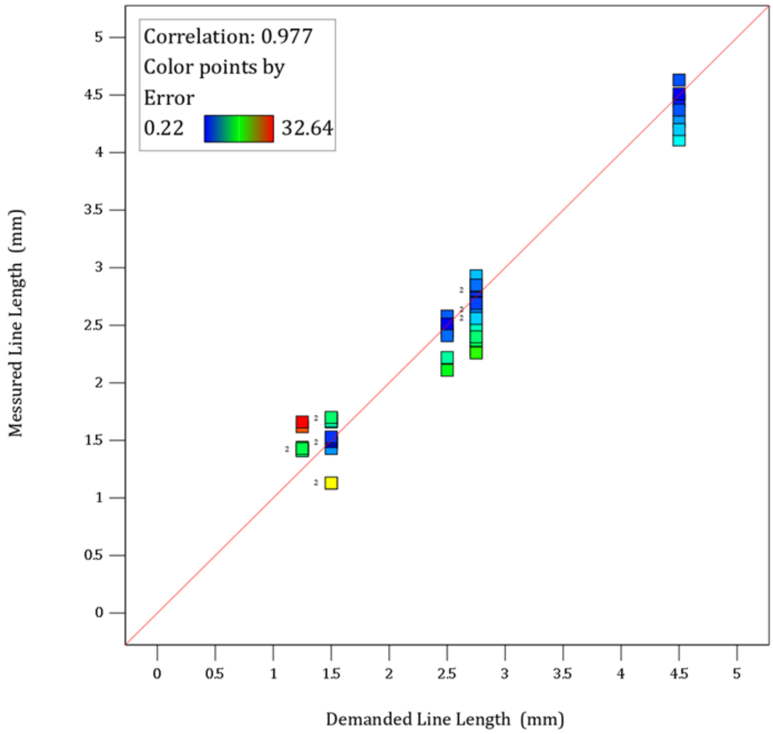

(b)

Fig 18: The scatter plot of demanded values versus the measured data for (a) the line length and (b) the line width

Designing for high-performance in additive manufacturing, despite its limitations, requires exploring various unit cells and meta-structures to identify patterns that interact effectively

with material behavior. In this research, the emphasis was primarily on numerical simulation and optimization of 3D-printed materials. The results were utilized to determine the parameters for experimental tests, the data of which has already been published [59-60]. For further investigations, the following subjects are suggested,

- Path planning and defining the route that the print head takes
- Providing topology optimization algorithms to achieve desired properties for specific applications
- Using exactly the material properties of the meta-structure and anisotropy (considering the 3D printing factors) for the lifetime prediction
- Examine the fatigue lifetime experimentally for the topology-optimized samples.

4) Conclusions

This research used multi-objective optimization to determine optimal parameters for PLA cylinders, considering fatigue lifetime, mass, and infill patterns. Structural topology optimization was conducted using a two-objective algorithm. Additionally, FESEM analysis ensured dimensional accuracy, and the influence of inherent defects in the FDM process was considered. The results are listed below:

- The stress analysis results showed that although the triangular pattern had the lowest mass in all cases, irregular octagon, rectangular and regular octagon had the lowest stresses in 40%, 60%, and 80% of the infill, respectively.
- The study introduced three sets of objective functions for design purposes, considering mass changes, fatigue lifetime changes, and the distance from the optimal point. The optimal solution was found to be the horizontally printed rectangular filling pattern with a 60% infill density. This pattern achieved a 39% reduction in weight while experiencing only a 22% decrease in fatigue lifetime. The mass reduction was determined through software analysis of designed CAD models, while the infill density was assessed using the ImageJ software.
- Topology optimization in Dassault Systems software proposed a metamaterial structure to minimize mass and strain energy under stress constraints. Achieved results: mass of 1.1767g, strain energy of 0.056 J (after 50 cycles), maximum von-Mises stress of 97.08 MPa. Microscopic inspections studied manufacturing defects and their impact on topology optimization.
- Shape optimization was performed to reduce stress concentration by displacing boundary nodes. Maximum von-Mises stress decreased to 44.30 MPa after 15 design cycles, leading to increased fatigue lifetime by 527 and 79 cycles compared to topology optimization in vertical and horizontal samples, respectively.
- Finally, a design realization step was performed, and a new geometry was introduced that has 96.70% lower stress and 201.69% higher mass compared with topology-optimized geometry. This is while this geometry is lighter than 60% infill samples with different patterns.
- Based on FESEM images, the rectangular pattern has the best regularity, while the regular octagon pattern was the most irregular.
- The line spacing is significant, especially in the honeycomb pattern, and should be controlled by printing temperature.
- The scatterplot shows a strong positive and linear relationship between the demanded and measured line length and line width.

- Inaccuracy in the position of the nozzle increases with the decrease in filling percentage and, therefore, the thickness of the walls. However, the rectangular sample with 40% infill and Regular Octagon with 60% infill was the worst parameters.

Declaration of competing interest

The authors illustrate that they have no competing financial interests or personal relationships that could have appeared to influence this work.

Data Availability

The datasets used and/or analyzed during the current study available from the corresponding author on reasonable request.

References

[1] "Additive manufacturing – General principles – Terminology, Standard," *Int. Organ. Stand.*, no. 52900:2015, 2015, doi: 10.1520/ISOASTM52900-15.

[2] M. Kun, C. Chan, S. Ramakrishna, A. Kulkarni, and K. Vadodaria, "Textile-based scaffolds for tissue engineering," in *Advanced Textiles for Wound Care*, Second Edi., Elsevier Ltd, 2019, pp. 329–362. doi: 10.1016/b978-0-08-102192-7.00012-6.

[3] F. N. Habib, P. Iovenitti, S. H. Masood, and M. Nikzad, "Fabrication of polymeric lattice structures for optimum energy absorption using multi jet fusion technology," *Mater. Des.*, vol. 155, pp. 86–98, 2018, doi: 10.1016/j.matdes.2018.05.059.

[4] S. M. Esfarjani, A. Dadashi, and M. Azadi, "Topology optimization of additive-manufactured metamaterial structures: A review focused on multi-material types," *Forces Mech.*, vol. 7, p. 100100, 2022, doi: 10.1016/J.FINMEC.2022.100100.

[5] S. Kumar, I. Singh, S. S. R. Koloor, D. Kumar, and M. Y. Yahya, "On Laminated Object Manufactured FDM-Printed ABS/TPU Multimaterial Specimens: An Insight into Mechanical and Morphological Characteristics," *Polymers (Basel).*, vol. 14, no. 19, p. 4066, 2022, doi: 10.3390/POLYM14194066.

[6] S. Kumar, I. Singh, D. Kumar, M. Y. Yahya, and S. S. Rahimian Koloor, "Mechanical and Morphological Characterizations of Laminated Object Manufactured 3D Printed Biodegradable Poly(lactic)acid with Various Physical Configurations," *J. Mar. Sci. Eng.*, vol. 10, no. 12, p. 1954, 2022, doi: 10.3390/JMSE10121954.

[7] J. Sun and N. M. Litchinitser, "Metamaterials," *Fundam. Appl. Nanophotonics*, pp. 253–307, 2016, doi: 10.1016/B978-1-78242-464-2.00009-9.

[8] H. Cui *et al.*, "Three-dimensional printing of piezoelectric materials with designed anisotropy and directional response," *Nat. Mater.*, vol. 18, pp. 234–241, 2019, doi: 10.1038/s41563-018-0268-1.

[9] X. Zheng *et al.*, "Ultralight, ultrastiff mechanical metamaterials," *Science (80-.).*, vol. 344, no. 6190, pp. 1373–1377, 2014, doi: 10.1126/science.1252291.

[10] S. Kumar, R. Singh, A. Batish, and T. P. Singh, "Additive manufacturing of smart materials exhibiting 4-D properties: A state of art review," vol. 35, no. 9, pp. 1358–1381, 2019, doi: 10.1177/0892705719895052.

[11] A. A. Zadpoor, "Meta-biomaterials," *Biomater. Sci.*, vol. 8, no. 1, pp. 18–38, 2019, doi: 10.1039/C9BM01247H.

[12] J. Fan *et al.*, "A review of additive manufacturing of metamaterials and developing trends," *Mater. Today*, vol. 50, pp. 303–328, 2021, doi:

10.1016/J.MATTOD.2021.04.019.
[13] W. Wu, P. Liu, and Z. Kang, "A novel mechanical metamaterial with simultaneous stretching- and compression-expanding property," *Mater.*, vol. 208, p. 109930, 2021, doi: 10.1016/j.matdes.2021.109930.
[14] G. Agrawal, A. Gupta, R. Chowdhury, and A. Chakrabarti, "Robust topology optimization of negative Poisson's ratio metamaterials under material uncertainty," *Finite Elem. Anal. Des.*, vol. 198, p. 103649, 2022, doi: 10.1016/J.FINEL.2021.103649.
[15] D. Li, W. Liao, N. Dai, G. Dong, Y. Tang, and Y. M. Xie, "Optimal design and modeling of gyroid-based functionally graded cellular structures for additive manufacturing," *CAD Comput. Aided Des.*, vol. 104, pp. 87–99, 2018, doi: 10.1016/j.cad.2018.06.003.
[16] Y. Tang, A. Kurtz, and Y. F. Zhao, "Bidirectional Evolutionary Structural Optimization (BESO) based design method for lattice structure to be fabricated by additive manufacturing," *CAD Comput. Aided*, vol. 69, pp. 91–101, 2015, doi: 10.1016/j.cad.2015.06.001.
[17] B. Fox and A. Subic, "An industry 4.0 approach to the 3D printing of composite materials," *Engineering*, vol. 5, no. 4, pp. 621–623, 2019, doi: 10.1016/j.eng.2019.06.003.
[18] A. P. Garland, B. C. White, B. H. Jared, M. Heiden, E. Donahue, and B. L. Boyce, "Deep convolutional neural networks as a rapid screening tool for complex additively manufactured structures," *Addit. Manuf.*, vol. 35, p. 101217, 2020, doi: 10.1016/j.addma.2020.101217.
[19] X. Qi, G. Chen, Y. Li, X. Cheng, and C. Li, "Applying neural-network-based machine learning to additive manufacturing: current applications, challenges, and future perspectives," *Engineering*, vol. 5, no. 4, pp. 721–729, 2019, doi: 10.1016/j.eng.2019.04.012.
[20] G. X. Gu, C. T. Chen, D. J. Richmond, and M. J. Buehler, "Bioinspired hierarchical composite design using machine learning: Simulation, additive manufacturing, and experiment," *Mater. Horizons*, vol. 5, pp. 939–945, 2018, doi: 10.1039/c8mh00653a.
[21] G. X. Gu, C. T. Chen, and M. J. Buehler, "De novo composite design based on machine learning algorithm," *Extrem. Mech. Lett.*, vol. 18, pp. 19–28, 2018, doi: 10.1016/j.eml.2017.10.001.
[22] C. Qian, R. K. Tan, and W. Ye, "Design of architectured composite materials with an efficient, adaptive artificial neural network-based generative design method," *Acta Mater.*, vol. 225, p. 117548, Feb. 2022, doi: 10.1016/J.ACTAMAT.2021.117548.
[23] Peter W. Christensen and A. Klarbring, "An Introduction to Structural Optimization," *Springer Sci. Bus. Media*, 2019.
[24] J. Zhu, H. Zhou, C. Wang, L. Zhou, S. Yuan, and W. Zhang, "A review of topology optimization for additive manufacturing: Status and challenges," *Chinese J. Aeronaut.*, vol. 34, no. 1, pp. 91–110, 2021, doi: 10.1016/j.cja.2020.09.020.
[25] J. P. Meng, B. Fang, and H. B. Tan, "Crystal structure of methyl 2-(4-(3-(2,4-difluorophenyl)pyrazolo[1,5-a]pyrimidin-6-yl)phenyl)acetate, C21H15F2N3O2," vol. 234, no. 4, pp. 805–807, 2019.
[26] J. Liu *et al.*, "Current and future trends in topology optimization for additive manufacturing," *Struct. Multidiscip. Optim.*, vol. 57, pp. 2457–2483, 2018, doi: 10.1007/s00158-018-1994-3.
[27] J. Kiendl and C. Gao, "Controlling toughness and strength of FDM 3D-printed PLA components through the raster layup," *Compos. Part B Eng.*, vol. 180, p. 107562, 2020,

doi: 10.1016/j.compositesb.2019.107562.
[28] S. Li, S. Yuan, J. Zhu, C. Wang, J. Li, and W. Zhang, "Additive manufacturing-driven design optimization: Building direction and structural topology," *Addit. Manuf.*, vol. 36, p. 101406, 2020, doi: 10.1016/j.addma.2020.101406.
[29] S. Li, "Why We need a global history of political thought," *Concepts Hist. John Dunn. Lect. China. Palgrave Macmillan, Singapore.*, 2021.
[30] J. Wu, A. Clausen, and O. Sigmund, "Minimum compliance topology optimization of shell–infill composites for additive manufacturing," *Comput. Methods Appl. Mech. Eng.*, vol. 326, pp. 358–375, 2017, doi: 10.1016/j.cma.2017.08.018.
[31] N. S. F. Jap, G. M. Pearce, A. K. Hellier, N. Russell, W. C. Parr, and W. R. Walsh, "The effect of raster orientation on the static and fatigue properties of filament deposited ABS polymer," *Int. J. Fatigue*, vol. 124, pp. 328–337, 2019, doi: 10.1016/j.ijfatigue.2019.02.042.
[32] T. Pepelnjak, J. Stojšić, L. Sevšek, D. Movrin, and M. Milutinović, "Influence of Process Parameters on the Characteristics of Additively Manufactured Parts Made from Advanced Biopolymers," *Polymers (Basel).*, vol. 15, no. 3, p. 716, 2023, doi: 10.3390/POLYM15030716.
[33] C. Abeykoon, P. Sri-Amphorn, and A. Fernando, "Optimization of fused deposition modeling parameters for improved PLA and ABS 3D printed structures," *Int. J. Light. Mater. Manuf.*, vol. 3, no. 3, pp. 284–297, 2020, doi: 10.1016/j.ijlmm.2020.03.003.
[34] T. N. A. T. Rahim, A. M. Abdullah, and H. Md Akil, "Recent developments in fused deposition modeling-based 3D printing of polymers and their composites," *Polym. Rev.*, vol. 59, no. 4, pp. 589–624, 2019, doi: 10.1080/15583724.2019.1597883.
[35] Y. G. Zhou, J. R. Zou, H. H. Wu, and B. P. Xu, "Balance between bonding and deposition during fused deposition modeling of polycarbonate and acrylonitrile-butadiene-styrene composites," *Polym. Compos.*, vol. 41, no. 1, pp. 60–72, 2020, doi: 10.1002/pc.25345.
[36] I. Bin Ishak, D. Fleming, and P. Larochelle, "Multiplane fused deposition modeling: a study of tensile strength," *Mech. Based Des. Struct. Mach.*, vol. 47, no. 5, pp. 583–598, 2019, doi: 10.1080/15397734.2019.1596127.
[37] A. K. Sood, R. K. Ohdar, and S. S. Mahapatra, "Parametric appraisal of mechanical property of fused deposition modelling processed parts," *Mater. Des.*, vol. 31, no. 1, pp. 287–295, 2010, doi: 10.1016/j.matdes.2009.06.016.
[38] M. Fernandez-Vicente, W. Calle, S. Ferrandiz, and A. Conejero, "Effect of Infill parameters on tensile mechanical behavior in desktop 3D printing," *3D Print. Addit. Manuf.*, vol. 3, no. 3, pp. 183–192, 2016, doi: 10.1089/3dp.2015.0036.
[39] R. Baptista and M. Guedes, "Porosity and pore design influence on fatigue behavior of 3D printed scaffolds for trabecular bone replacement," *J. Mech. Behav. Biomed. Mater.*, vol. 117, p. 104378, 2021, doi: 10.1016/j.jmbbm.2021.104378.
[40] T. Pepelnjak, A. Karimi, A. Maček, and N. Mole, "Altering the Elastic Properties of 3D Printed Poly-Lactic Acid (PLA) Parts by Compressive Cyclic Loading," *Mater*, vol. 13, no. 19, p. 4456, 2020, doi: 10.3390/MA13194456.
[41] N. A. Fountas, J. D. Kechagias, D. E. Manolakos, and N. M. Vaxevanidis, "Single and multi-objective optimization of FDM-based additive manufacturing using metaheuristic algorithms," *Procedia Manuf.*, vol. 51, pp. 740–747, 2020, doi: 10.1016/J.PROMFG.2020.10.104.
[42] N. Yodo and A. Dey, "Multi-Objective Optimization for FDM Process Parameters with

Evolutionary Algorithms," pp. 419–444, 2021, doi: 10.1007/978-3-030-68024-4_22.
[43] J. John, D. Devjani, S. Ali, S. Abdallah, and S. Pervaiz, "Optimization of 3D printed polylactic acid structures with different infill patterns using Taguchi-grey relational analysis," *Adv. Ind. Eng. Polym. Res.*, 2022, doi: 10.1016/J.AIEPR.2022.06.002.
[44] C. Abeykoon, P. Sri-Amphorn, and A. Fernando, "Optimization of fused deposition modeling parameters for improved PLA and ABS 3D printed structures," *Int. J. Light. Mater. Manuf.*, vol. 3, no. 3, pp. 284–297, 2020, doi: 10.1016/J.IJLMM.2020.03.003.
[45] B. N. Turner and S. A. Gold, "A review of melt extrusion additive manufacturing processes: II. Materials, dimensional accuracy, and surface roughness," *Rapid Prototyp. J.*, vol. 21, no. 3, pp. 250–261, 2015, doi: 10.1108/RPJ-02-2013-0017.
[46] S. H. Ahn, C. Baek, S. Lee, and I. S. Ahn, "Anisotropic Tensile Failure Model of Rapid Prototyping Parts - Fused Deposition Modeling (FDM)," *Int. J. Mod. Phys. B*, vol. 17, no. 08n09, pp. 1510–1516, 2003, doi: 10.1142/S0217979203019241.
[47] S. Farah, D. G. Anderson, and R. Langer, "Physical and mechanical properties of PLA, and their functions in widespread applications — A comprehensive review," *Adv. Drug Deliv. Rev.*, vol. 107, pp. 367–392, 2016, doi: 10.1016/j.addr.2016.06.012.
[48] O. H. Ezeh and L. Susmel, "On the fatigue strength of 3D-printed polylactide (PLA)," *Procedia Struct. Integr.*, vol. 9, pp. 29–36, 2018, doi: 10.1016/j.prostr.2018.06.007.
[49] S. Dezianian and M. Azadi, "Multi-Material Metamaterial Topology Optimization to Minimize the Compliance and the Constraint of Weight: Application of Non-Pneumatic Tire Additive-Manufactured with PLA/TPU Polymers," *Polymers (Basel).*, vol. 15, no. 8, p. 1927, 2023, doi: 10.3390/POLYM15081927.
[50] A. Karimi, N. Mole, and T. Pepelnjak, "Numerical Investigation of the Cycling Loading Behavior of 3D-Printed Poly-Lactic Acid (PLA) Cylindrical Lightweight Samples during Compression Testing," *Appl. Sci.*, vol. 12, no. 16, p. 8018, 2022, doi: 10.3390/APP12168018.
[51] B. Kosic, M. Stojicevic, Z. Jeli, B. Pokonstantinovic, A. Duta, and A. Dragicevic, "3D analysis of diferent metamaterial geometry and simulation of metamaterial usage," *FME Trans.*, vol. 47, no. 2, pp. 349–354, 2019, doi: 10.5937/fmet1902349K.
[52] J. Antonio Travieso-Rodriguez, M. D. Zandi, R. Jerez-Mesa, and J. Lluma-Fuentes, "Fatigue behavior of PLA-wood composite manufactured by fused filament fabrication," *J. Mater. Res. Technol.*, vol. 9, no. 4, pp. 8507–8516, 2020, doi: 10.1016/j.jmrt.2020.06.003.
[53] S. N. I. 1143, "Metallic materials - Rotating bar bending fatigue testing," *ISO Int. Stand.*, 2010.
[54] DASSAULT SYSTEMES and Simulia, "Abaqus Analysis User's Manual," 2016.
[55] M. S. Aghareb Parast, A. Bagheri, A. Kami, M. Azadi, and V. Asghari, "Bending fatigue behavior of fused filament fabrication 3D-printed ABS and PLA joints with rotary friction welding," *Prog. Addit. Manuf.*, pp. 1–17, 2022, doi: 10.1007/S40964-022-00307-5.
[56] M. Azadi, A. Dadashi, S. Dezianian, M. Kianifar, S. Torkaman, and M. Chiyani, "High-cycle bending fatigue properties of additive-manufactured ABS and PLA polymers fabricated by fused deposition modeling 3D-printing," *Forces Mech.*, vol. 3, p. 100016, Sep. 2021, doi: 10.1016/j.finmec.2021.100016.
[57] N. Wang, W. jie Zhao, N. Wu, and D. Wu, "Multi-objective optimization: A method for selecting the optimal solution from Pareto non-inferior solutions," *Expert Syst. Appl.*, vol. 74, pp. 96–104, 2017, doi: 10.1016/j.eswa.2017.01.004.

[58] A. R. Aziz, J. Zhou, D. Thorne, and W. J. Cantwell, “Geometrical Scaling Effects in the Mechanical Properties of 3D-Printed Body-Centered Cubic (BCC) Lattice Structures,” *Polym*, vol. 13, no. 22, p. 3967, 2021, doi: 10.3390/POLYM13223967.
[59] M. Azadi and A. Dadashi, “Experimental fatigue dataset for additive-manufactured 3D-printed Polylactic acid biomaterials under fully-reversed rotating-bending bending loadings,” *Data Br.*, vol. 41, p. 107846, 2022, doi: 10.1016/J.DIB.2022.107846.
[60] A. Dadashi and M. Azadi, “Experimental bending fatigue data of additive-manufactured PLA biomaterial fabricated by different 3D printing parameters,” *Prog. Addit. Manuf.*, 2022, doi: 10.1007/S40964-022-00327-1.
[61] H. Gonabadi, A. Yadav, and S. J. Bull, “The effect of processing parameters on the mechanical characteristics of PLA produced by a 3D FFF printer,” *Int. J. Adv. Manuf. Technol.*, vol. 111, pp. 695–709, 2020, doi: 10.1007/s00170-020-06138-4.
[62] B. Aloyaydi, S. Sivasankaran, and A. Mustafa, “Investigation of infill-patterns on mechanical response of 3D printed poly-lactic-acid,” *Polym. Test.*, vol. 87, p. 106557, 2020, doi: 10.1016/j.polymertesting.2020.106557.
[63] A. D. Pertuz, S. Díaz-Cardona, and O. A. González-Estrada, “Static and fatigue behaviour of continuous fibre reinforced thermoplastic composites manufactured by fused deposition modelling technique,” *Int. J. Fatigue*, vol. 130, p. 105275, 2020, doi: 10.1016/j.ijfatigue.2019.105275.
[64] J. Naranjo-Lozada, H. Ahuett-Garza, P. Orta-Castañón, W. M. H. Verbeeten, and D. Sáiz-González, “Tensile properties and failure behavior of chopped and continuous carbon fiber composites produced by additive manufacturing,” *Addit. Manuf.*, vol. 26, pp. 227–241, Mar. 2019, doi: 10.1016/j.addma.2018.12.020.
[65] V. Cabreira and R. M. C. Santana, “Effect of infill pattern in Fused filament fabrication (FFF) 3D printing on materials performance,” *Matéria*, vol. 25, no. 3, pp. 1–9, 2020.
[66] K. Vigneshwaran and N. Venkateshwaran, “Statistical analysis of mechanical properties of wood-PLA composites prepared via additive manufacturing,” *Int. J. Polym. Anal. Charact.*, vol. 24, no. 7, pp. 584–596, 2019, doi: 10.1080/1023666X.2019.1630940.
[67] H. Mei, Z. Ali, I. Ali, and L. Cheng, “Tailoring strength and modulus by 3D printing different continuous fibers and filled structures into composites,” *Adv. Compos. Hybrid Mater.*, vol. 2, pp. 312–319, 2019, doi: 10.1007/s42114-019-00087-7.
[68] M. Frascio, M. Avalle, and M. Monti, “Fatigue strength of plastics components made in additive manufacturing: First experimental results,” *Procedia Struct. Integr.*, vol. 12, pp. 32–43, 2018, doi: 10.1016/j.prostr.2018.11.109.
[69] A. A. Ansari and M. Kamil, “Effect of print speed and extrusion temperature on properties of 3D printed PLA using fused deposition modeling process,” *Mater. Today Proc.*, vol. 45, pp. 5462–5468, 2021, doi: 10.1016/j.matpr.2021.02.137.
[70] B. N. Turner, R. Strong, and S. A. Gold, “A review of melt extrusion additive manufacturing processes: I. Process design and modeling,” *Rapid Prototyp. J.*, vol. 20, no. 3, pp. 192–204, 2014, doi: 10.1108/RPJ-01-2013-0012/FULL/XML.
[71] S. Singamneni, M. P. Behera, D. Truong, M. J. Le Guen, E. Macrae, and K. Pickering, “Direct extrusion 3D printing for a softer PLA-based bio-polymer composite in pellet form,” *J. Mater. Res. Technol.*, vol. 15, pp. 936–949, Nov. 2021, doi: 10.1016/J.JMRT.2021.08.044.

Supporting information

Fig. S1: The GIF file for the developments during the topology optimization

Click here to access/download
Supporting Information
TopologyOptimization.gif

Figures

Click here to access/download
Supporting Information
Fig.rar

Multi-objective numerical optimization of 3D-printed polylactic acid bio-metamaterial based on topology, filling pattern, and infill density via fatigue lifetime and mass

Ali Dadashi[1] and Mohammad Azadi[1*]

[1] Research Laboratory of Advanced Materials Behavior (AMB), Faculty of Mechanical Engineering, Semnan University, Semnan, Iran
[*]Corresponding Author, Email: m_azadi@semnan.ac.ir

Abstract

Infill parameters are significant with regard to the overall cost and saving material while printing a 3D model. When it comes to printing time, we can decrease the printing time by altering the infill, which also reduces the total process extent. Choosing the right filling parameters affects the strength of the printed model. In this research, the effect of filling density and infill pattern on the fatigue lifetime of cylindrical polylactic acid (PLA) samples was investigated with finite element modeling and analysis. This causes the lattice structure to be considered macro-scale porosity in the additive manufacturing process. Due to the need for multi-objective optimization of several functions at the same time and the inevitable sacrifice of other objectives, the decision was to obtain a set of compromise solutions according to the Pareto-optimal solution technique or the Pareto non-inferior solution approach. As a result, a horizontally printed rectangular pattern with 60% filling was preferred over the four patterns including honeycomb, triangular, regular octagon, and irregular octagon by considering the sum of mass changes and fatigue lifetime changes, and distance from the optimal point, which is the lightest structure with the maximum fatigue lifetime as an objective function with an emphasis on mass as an important parameter in designing scaffolds and biomedical structures. A new structure was also proposed by performing a structural optimization process using computer-aided design tools and also, computer-aided engineering software by Dassault systems. Finally, the selected samples were printed and their 3D printing quality was investigated using field emission scanning electron microscopy inspection.

Keywords: Metamaterial; Multi-objective optimization; Fatigue lifetime; 3D printing quality; Finite element modeling

1) Introduction

The functionality of a fabricated component relies on the combination of properties and geometry, achieved through a series of fabrication techniques [1]. Additive manufacturing (AM) processes offer advantages such as complex 3D geometries and flexible construction of layered structures [2]. Tailoring the build formulation allows for gradual changes in composition or lattice structures [3-4]. For instance, Kumar et al. [5-6] compared multi-blended and hybrid blended PLA matrix in 3D-printed tensile specimens, finding superior mechanical and morphological properties in the multi-blended matrix, reflecting the growing interest in exploring the potential of additive manufacturing.

AM techniques enable the fabrication of metamaterials with unique properties, such as negative Poisson ratio and high strength [7-9]. These metamaterials exhibit extraordinary functions in acoustics, electromagnetics, and mechanics [10]. Bio-metamaterials, designed with geometrical structures at smaller scales, mimic the properties of living tissues and are applied in orthopedic biomaterials for bone tissue regeneration [11]. Although the feasibility and reliability of these processes are still recognized as a challenge [12]. Wu et al. [13] developed a novel mechanical metamaterial with lateral expansion, validated through simulations and experiments. Agrawal et al. [14] proposed a robust topology optimization method for negative Poisson ratio metamaterials, ensuring stable designs with low variation. Researchers have utilized topology optimization (TO) and additive manufacturing (AM) to enhance the design and fabrication of structurally optimized cellular structures [15-16]. The integration of artificial intelligence (AI) and AM has introduced a new design principle, enabling efficient structure and component design through machine learning techniques [17-21]. Qian et al. [22] employed an artificial neural network-based inverse design method, demonstrating improved efficiency and reduced training data requirements for designing architected composite parts. Their approach showcased superior performance compared to other machine-learning approaches.

In order to further increase the mechanical properties of parts fabricated by AM, designing and optimizing approaches have been introduced and developed at various scales. Specifically, on the macroscale, structural optimizations including shape, size, and topology optimization techniques could be considered for guiding the component design [23-24]. Between these methods, polymeric composites can achieve their maximum potential by AM-driven TO [25]. Manufacturing constraints such as minimum size and connectivity limit the optimization according to the manufacturing ability [26]. The anisotropic material property, besides manufacturing constraints, is another parameter that should be applied during the process of designing and optimizing [27]. A transversely isotropic material model and solid anisotropic material with penalization coupled with an AM-driven topology optimization technique were proposed by Li et al. [28-29]. Due to the layered fabrication process, they achieved mechanical improvement in stiffness and strength because of taking advantage of anisotropy. Wu et al. [30] presented a novel method for generating simultaneously optimized solid shell and porous infill in the context of maximum stiffness topology optimization. A material interpolation model, upon which the compliance is minimized, unified the resulting intermediate density distributions while all the fibers follow the direction of the principal stress.

3D printing process parameters have a significant impact on the mechanical properties of printed parts, including printing temperature, speed, filling density, pattern, layer thickness, material properties, surface roughness, and layer thickness [31-33]. Researchers have conducted several studies to further clarify these effects [34-35]. Manufacturing 3D-printed

products requires a clear understanding of the mechanical characteristics of 3D-printed components [36]. Sood et al. [37] found that increasing the layer thickness in ABS 3D-printed components improved impact and bending properties, but had an inverse relationship with tensile performance. Fernandez Vicente et al. [38] examined the influence of different infill patterns on the tensile performance of ABS 3D-printed parts. They found that ABS components with line and honeycomb infill patterns exhibited higher tensile strength compared to those with a rectilinear infill pattern. Baptista and Guedes [39] investigated the impact of infill density and patterns on the static and fatigue characteristics of scaffolds. Lower infill density resulted in higher yield stress and showed no significant fatigue damage after 3600 load cycles. Moreover, the influence of infill line distance on the compressive elastic behavior of 3D printed circular samples was investigated by Pepelnjak et al. [40] They observed a significant difference in the first cycle response and an alteration of stiffness due to elastic and plastic deformation under cyclic.

Fountas et al. [41] examined the performance of different swarm-based evolutionary algorithms in optimizing single and multi-objective problems related to additive manufacturing, specifically fused deposition modeling (FDM). The results showed that algorithms do not perform equally when applied to different optimization problems, and the quality of Pareto non-dominated solutions is an important indicator for multi-objective problems. Yodo and Dey [42] introduced multi-objective optimization methods based on evolutionary algorithms to optimize FDM process parameters, leading to Pareto-optimal solutions for decision-makers.

So far, the literature has been reviewed and by summarizing the presented materials, it can be said that researchers have focused on the following topics:

- The extraordinary properties of meta-materials [8-9], [13]
- Providing optimization algorithms to improve mechanical behavior [17-23]
- Designing optimized structures in different dimensions [26-30]
- Applying additive manufacturing conditions in the optimization process [31-39], [43-46]
- Introducing Pareto-optimal solutions for decision-makers [41-42]

The article discusses the multi-objective numerical optimization of 3D-printed polylactic acid (PLA) bio-metamaterial. The optimization takes into account the factors of topology, filling pattern, and infill density in order to improve the fatigue lifetime and mass of the material. Therefore, the novelties of the study are as follows,

- The use of multi-objective optimization to find the optimum value of topology, filling pattern, and infill density for 3D-printed polylactic acid bio-metamaterial.
- The consideration of both fatigue lifetime and mass in the optimization process.
- The use of field emission scanning electron microscopy (FESEM) images to investigate the dimensional accuracy of 3D-printed components.

2) Material and Methodology

2-1) Material Properties

Despite the effect of different manufacturing parameters on the response of the polymers, the overall mechanical properties of PLA used in this research are listed in Table 1, based on literature [47-48]. Density, elastic module, yield strength, ultimate tensile strength, and Poisson ratio were obtained from technical datasheets provided by the material manufacturer. These properties are typically reported by the manufacturer based on

standard testing methods [47]. Though, endurance limit, which refers to the maximum stress amplitude that a material can withstand for a given number of cycles without failure, cannot be obtained from technical datasheets and requires experimental testing. The endurance limit used in this study [48] was experimental obtained. It should be noted that YouSu brand polylactic acid (PLA) filament, was used in component fabrication for 3D printing. The properties of 3D printed parts can differ from those of the raw filament material. For example, while the density of PLA filament was typically reported as 1.24 g/cm^3 in manufacturer datasheets based on the ASTM D792 standard test method, 3D printed parts can have a different density due to their meta-structure. Additionally, the tensile module and yield strength of PLA filament are reported as 3.5 GPa and 60 MPa, respectively, based on ASTM D1238 standard test method. However, 3D printed parts may exhibit different values for these properties due to the complex interplay of factors such as infill percentage, layer height, printing speed, and other printing parameters. The endurance limit for 95% probability of survival and determined in terms of maximum stress and extrapolated at 2 million cycles to failure cycles, is considered equal to 0.1 final stress [48].

Table 1: Mechanical properties of 3D-printed PLA [47-48]

Characteristics	Unit	Amount
Density	gr/cm^3	1.252
elastic module	MPa	1280
Yield strength	MPa	70
Ultimate tensile strength	MPa	73
Poisson ratio	-	0.36
Endurance limit	MPa	7.3

The compressive and tensile behaviors of PLA are usually considered to be the same, but some studies have focused specifically on the compressive behavior of PLA. These studies aim to better understand how PLA responds to compressive forces and how it can be used in applications where compression is a dominant mode of loading, such as in bone tissue engineering and orthopedics [49-50].

2-2) Numerical Simulation

Structural optimization is an iterative technique that could help the refinement of designs. Then, a result of the proper-designed structural optimization could be a lightweight part, which has durable properties. An integrated computer-aided design (CAD) and computer-aided engineering (CAE) workflow by Dassault systems was proposed (Fig 1).

The use of additive manufacturing enables the fabrication of different patterns with varying mechanical properties. The filling parameters have a direct effect on the response of the metamaterials to external loadings. For instance, a recent study [51] on metal functionally graded gyroids employed three design methods (thickness graded, size graded, and uniform) to provide a map of the mechanical properties of stainless-steel scaffolds. In the proposed study, we selected five infill patterns, including honeycomb, rectangular, triangular, irregular octagon [51], and regular octagon [51] to investigate their effect on fatigue properties and mass, and the results suggest that further investigations on other patterns may be necessary, to compare the fatigue behavior. The infill patterns used in this study are shown in Fig 2.

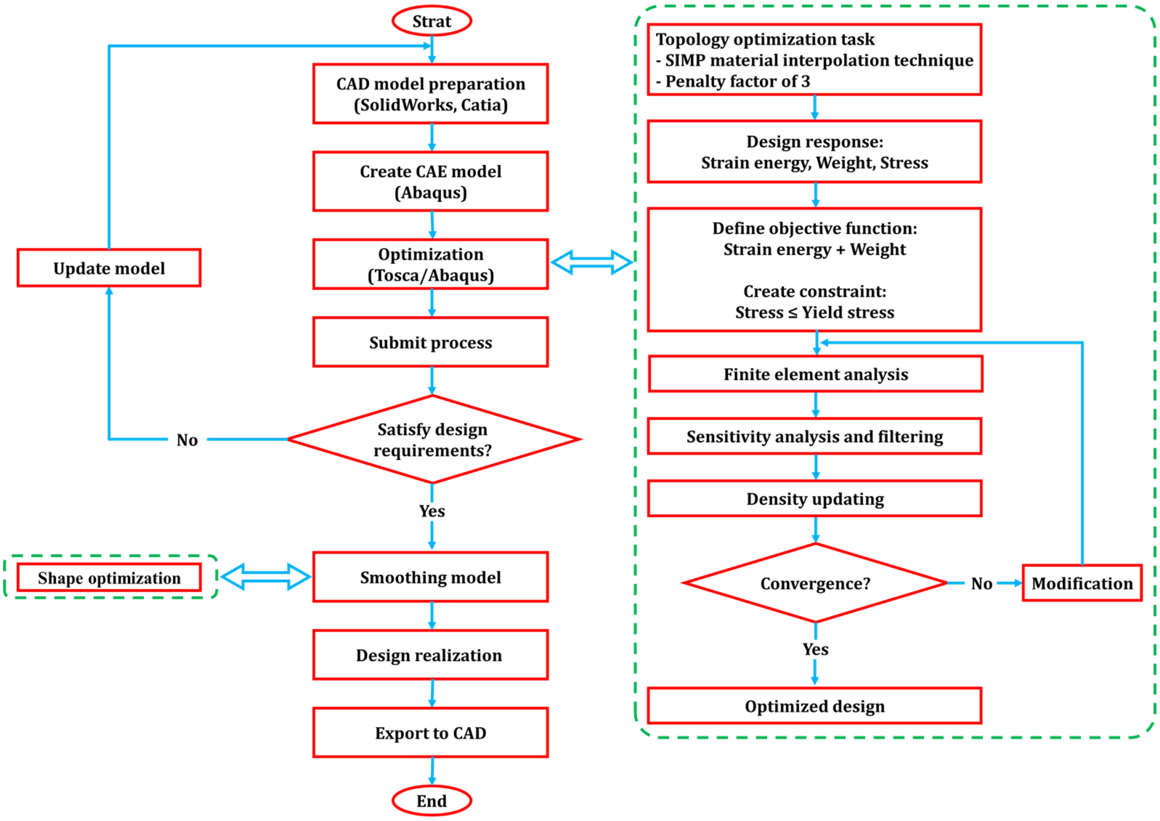

Fig 1: The workflow of topological designing using Dassault systems software

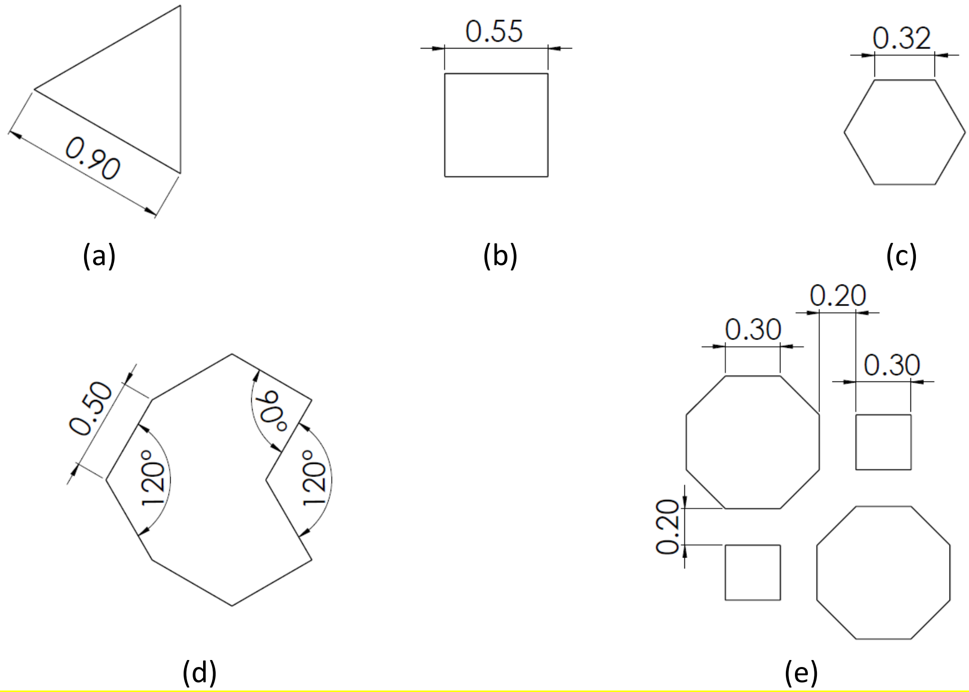

Fig 2: Different filling patterns: (a) triangular, (b) rectangular, (c) honeycomb, (d) irregular octagon, and (e) regular octagon

Based on the flow chart, a geometric model of the structure should be initially prepared in CAD software, including SolidWorks or Catia. Considering Table 2, filling densities of 40%, 60%, 80%, and 100% were also designed in the section of a cylinder with an outer diameter

of 9 mm and a height of 78 mm, by SolidWorks software. To achieve desired filling densities these geometries were arranged next to each other at different distances. In addition, two solid layers are considered as a perimeter with a width of 0.4 mm for each layer [52].

Table 2: The cross-section of structures for metamaterials

Fill pattern	40%	60%	80%
Honeycomb	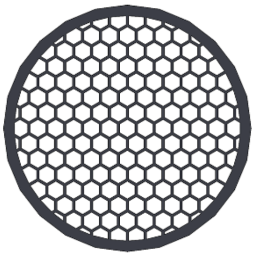	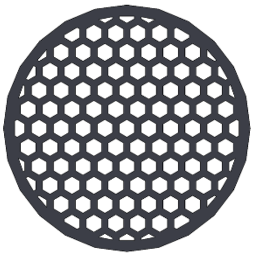	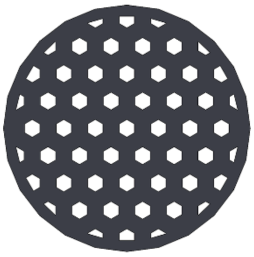
Rectangular	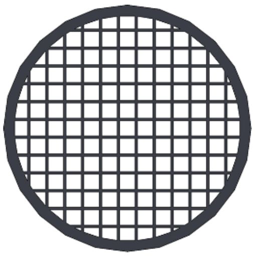	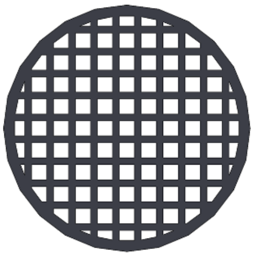	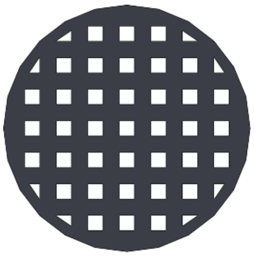
Triangular	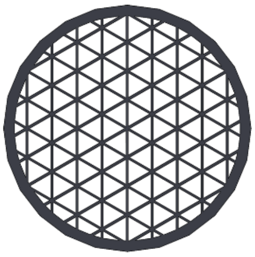	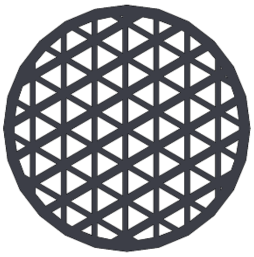	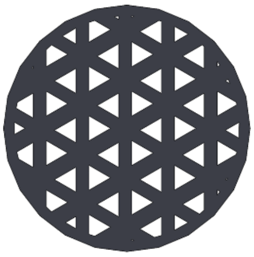
Regular octagon	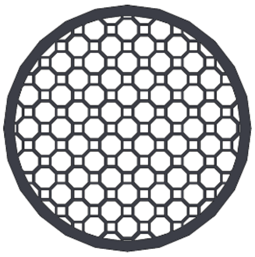	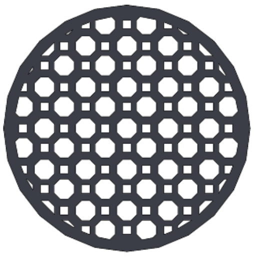	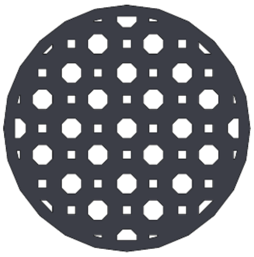
Irregular octagon	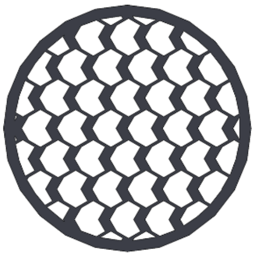	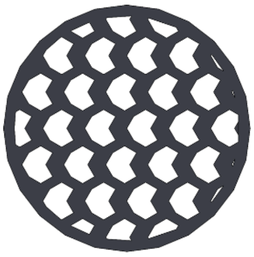	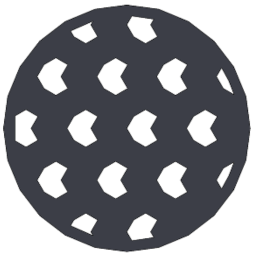
Full filled (100%)		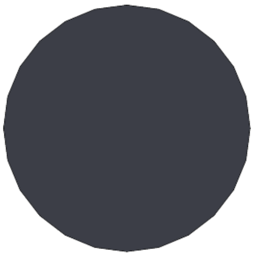	

A finite element model is fabricated in SIMULIA- ABAQUS for stress analysis besides the topology optimization. To calculate bending stress according to the rotating-bending fatigue testing method [53], the samples were subjected to a 10 N load at the free end of a cantilever which is clamped at the other end; therefore, the displacements of the supported end are constrained in three directions (Fig 3(a)). In the rotating-bending test, a common method used to evaluate the high cycle fatigue behavior of materials, the specimen is subjected to cyclic loading through rotational motion. During the rotating-bending test, the applied stress levels are typically kept below the yield strength of the material. This ensures that the deformation experienced by the specimen remains within the elastic range (<70 MPa [47]), where the material can fully recover its original shape after each cycle. The focus is on assessing the material performance to withstand cyclic loading without undergoing plastic deformation or failure. In this regard, Static, General simulations were performed in Abaqus/Standard. Regarding the mesh size, 8-node linear hexahedral solid elements with reduced integration (C3D8R) are considered with the global size of 0.7 mm to provide optimal accuracy and decrease CPU time and storage requirements (Fig 3(b)) [54]. Therefore, the number of elements meshed in each sample is listed in Table 3. There were some areas with thickness less than 0.7 mm. In such cases, to prevent the distorted elements in these thin areas, the minimum size control parameter (set to 0.1 of the global size) and the curvature control parameter (set with a maximum deviation factor of 0.1) adjusted the element size. Increasing the maximum deviation factor allows for a greater deviation between the actual geometry and the approximated geometry, and can be useful in ensuring mesh quality in thin regions. The minimum size control and curvature control parameters used in this study were recommended by the ABAQUS software algorithm.

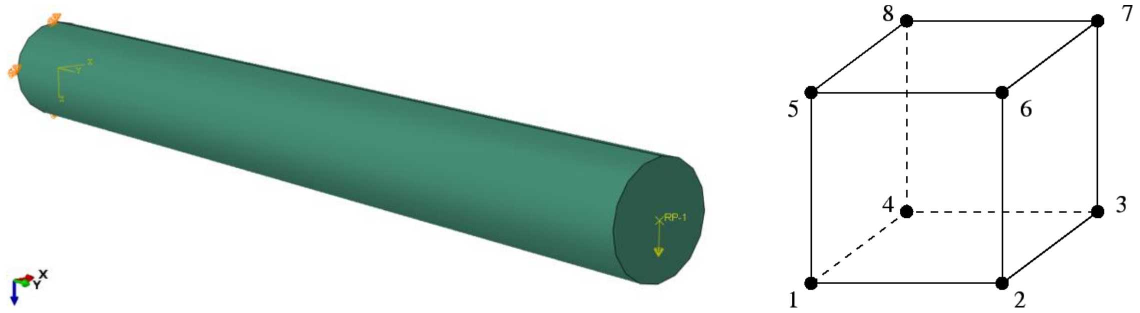

(a) (b)

Fig 3: (a) The applied load and boundary conditions and (b) the C3D8R element

Table 3: The number of elements in different models

Fill pattern / Infill density	40%	60%	80%	100%
Honeycomb	163947	93129	124653	17538
Rectangular	102897	51837	22644	
Triangular	149850	73704	90021	
Regular octagon	118690	13961397	131262	
Irregular octagon	101232	93936	26418	

2-3) Topology and Shape Optimization

Topology optimization is a proper method for designing that provides engineers with a powerful code in order to evaluate the solution space and enhance the designing reliability for any structures, in macro- and micro-scales. A wide range of various techniques exists such

as the solid isotropic material with penalization (SIMP) or the rational approximation of material properties (RAMP). Then, the tool carries out several iterations to find a consecutive answer by redesigning and simulating the structure. As the RAMP interpolation scheme is suitable for dynamic problems, the SIMP algorithm was used in this study.

For this topology optimization, the superior technique is the SIMP approach, which estimates an optimal distribution of the material under boundary conditions, loadings, manufacturing constraints, and any other requirements for the performance and properties.

The main technique in topology optimization is to discretize the solution domain into a grid of finite elements, which is entitled isotropic solid microstructures. Then, two events occur either the element is materially filled or the element becomes a void without a material (material-removing). This issue could be written as follows in mathematics by the density distribution of the material (ρ), which is a discrete binary value [54],

- $\rho_{(e)} = 1$ for material requiring,
- $\rho_{(e)} = 0$ for material removing.

Then, since the material density is a continuous value; therefore, the elastic module varies continuously in all elements, as the following equation [54],

$$E(\rho_e) = \rho_e^p E_0 \quad (1)$$

In this power law for each element (e), ρ_e is the relative density of the material, E_0 is the elastic module, and finally, p is a factor for the penalty which shows the contribution of elements with intermediate values of the density to the total stiffness. The literature illustrated that the superior value for the penalty factor is 3 [54].

As known, when the elastic module reduces, the element stiffness decreases. Based on the SIMP approach, the stiffness could be calculated as follows [54],

$$K_{SIMP}(\rho) = \sum_{e=1}^{N} [\rho_{min} + (1 - \rho_{min})\rho_e^p] K_e \quad (2)$$

In this formulation, ρ_{min} is the minimum relative density and K_e is the matrix for the element stiffness, where N is the element number. As a note, through the optimization iteration, a sensitivity analysis is done in order to find the influence of the density variation on the objective of the problem.

In the present study, the full-filled model was also used to find a topologically optimized structure. Then, the objectivities and the constraints were defined for the optimization in the topological design technique by simulation-based SIMULIA-Tosca, with a sensitivity-based optimizer to find an optimized trend for designing.

The optimization objective was to minimize the structure mass (the mass summation for all elements, ΣW_e) and also the strain energy (the compliance of a structure which is defined as the summation of the strain energy for all elements, $\Sigma u^t K u$. In this case, u is the displacement and K is the matrix to the global stiffness). This job was done under the stress constraint, which must be less or equal to 70 MPa which is the yield stress of polylactic acid (PLA). It should be noted that because of reduced relative density during the topology optimization, interpolation is required to calculate the stresses of elements, so the scaled element centroidal von-Mises stress of the design area is used in the optimization process [54],

$$Stress = \max \left| \frac{(\sigma_{vMises})^2}{(f(\rho_i)\sigma_y)^2} . \sigma_y \right| \quad (3)$$

where, σ_{vMises} is the element centroidal von-Mises stress, and ρ_i demonstrates a parameter for interpolating the stress in the element. This issue leads to a reduction in the current

relative density due to the topology optimization. Moreover, the reference stress, σ_y is given as follow [54],

$$\sigma_y = \min\left\{\alpha_1 \max\left|\frac{(\sigma_{vMises})^2}{\left(f(\rho_i)\sigma_{ref_1}\right)^2}.\sigma_{ref_1}\right| + \alpha_2 \max\left|\frac{(\sigma_{vMises})^2}{\left(f(\rho_i)\sigma_{ref_2}\right)^2}.\sigma_{ref_2}\right|\right\} \quad (4)$$

α_1 and α_2 are the weighting factors and σ_{ref} is the reference stress calculated during the initial optimization iteration [54].

As an important note, a mesh smoothing process was used by an additional shape optimization. This job was to have better quality for elements and also to minimize the value of von-Mises stress, as a minor modification. In other words, shape optimization was utilized in order to minimize the stress concentration in the finite element model, as a stress homogenization process [54].

Failing to properly realize the optimized design may result in a product that does not perform as intended or that is not feasible to manufacture, and therefore, it is crucial to carefully consider a design realization step in the topology optimization process to achieve a feasible component design concerning the manufacturing constraints. Therefore, for further post-processing development, some modifications were performed manually inspired by the geometry resulting from shape optimization, used to redesign a new part.

2-4) Fatigue Performance

To evaluate the fatigue lifetime, equation (5) was used [55]:

$$\sigma_a = \sigma_f'\left(2N_f\right)^b \quad (5)$$

in which b is the fatigue strength exponent, and σ_f' is the fatigue strength coefficient. Besides, σ_a is the stress amplitude which is equal to the maximum stress obtained from numerical simulation. Now the fatigue lifetime, N_f, can be calculated.

These parameters are material constants and differ notably when the printing conditions change. Nevertheless, b and σ_f' are provided in Table 4 for two print directions based on the previous studies by the authors [56]. In this table, fatigue tests were conducted at each stress level three times. Then, an S-N diagram was provided that fitted all the data. Moreover, the average of the results at each stress level was calculated and then again, another S-N diagram was provided. Although these material properties in Table 4 are not exactly as same as the material behavior of the fabricated samples in this work; they could be an initial prediction for the lifetime estimation. Besides, in this work, the infill density and the filling pattern were considered to study. These parameters could have effects on the stress value. This influence was also investigated by microscopic images, as to check the 3D printing quality.

Table 4: The parameters of the material including fatigue properties [56]

Material / Print direction		All data			Mean value		
		b	σ_f'	R^2	b	σ_f'	R^2
PLA	Horizontal	-0.2850	321.27	0.9680	-0.2880	334.29	0.9805
	Vertical	-0.2880	180.25	0.9573	-0.3010	207.90	0.9993

2-5) Two-Objective Optimization

A quantitative technique for solving two-objective optimization was used to find the optimized value of filling parameters. Therefore, using these optimization objectives leads to having a minimal mass and a maximal fatigue lifetime. In this case, three sets of objective

functions were considered. The first one uses the mass (M) and fatigue lifetime (N_f). The mass can be calculated with the production of material density and the volume of each specimen by fatigue lifetime is also obtained from equation 5. Mass reduction ratio and fatigue lifetime reduction ratio compared to the solid sample according to Table 5 are the second set of objective functions. The third set includes the distance from the optimal point (maximum mass loss and least lifetime reduction), D, and the sum of mass and fatigue lifetime, S.
In Table 5, M is mass and N_f is the fatigue lifetime. $\Delta N_f\%$ and $\Delta M\%$ are changed in fatigue lifetime and mass compared to the solid sample, respectively. $N_{f(solid)}$ and M_{solid} are the fatigue lifetime and mass of the solid sample, S is the sum of mass and fatigue lifetime changes, and D is the distance from the optimal point. Due to the decrease in mass compared to the solid sample, the mass change is considered negative.

Table 5: The combination of objective functions

Set	f_1	f_2
Set 1	max: $N_f = 0.5 \times \left(\frac{\sigma_a}{\sigma'_f}\right)^{\frac{1}{b}}$	min: $M = \rho V$
Set 2	min: $\Delta N_f\% = \frac{N_{f(solid)}-N_f}{N_{f(solid)}} \times 100\%$	min: $\Delta M\% = -\frac{M_{solid}-M}{M_{solid}} \times 100\%$
Set 3	min: $S = \Delta N_f\% + \Delta M\%$	min: $D = \sqrt{(\Delta N_f\%)^2 + (\Delta M\% + 100\%)^2}$

Considering different objectives in the Pareto front diagram, the performance-price ratio was used as an optimization reference. These objectives are similar to the fatigue lifetime and the mass (Set 3 in Table 5). Higher fatigue lifetime means better performance and lower mass means lower cost.
Accordingly, all stages of this analysis could be summarized based, as follows [57],
Step 1: Sorting the solutions in the diagram of the Pareto front by the objective (f_1) in ascending behavior [57].
Step 2: Considering the average line slopes for the average variability, based on objective functions (f_1 and f_2). The formulations for these slopes ($k_1^{(m)}$ and $k_2^{(m)}$) are as follows [57],

$$k_1^{(m)} = \frac{1}{2}\left(\frac{f_2^{(m)} - f_2^{(m-1)}}{f_1^{(m)} - f_1^{(m-1)}} + \frac{f_2^{(m+1)} - f_2^{(m)}}{f_1^{(m+1)} - f_1^{(m)}}\right), m = 2, 3, \dots, M-1 \quad (6)$$

$$k_1^{(m)} = \frac{1}{2}\left(\frac{f_1^{(m)} - f_1^{(m-1)}}{f_2^{(m)} - f_2^{(m-1)}} + \frac{f_1^{(m+1)} - f_1^{(m)}}{f_2^{(m+1)} - f_2^{(m)}}\right), m = 2, 3, \dots, M-1 \quad (7)$$

Let M denote the number of Pareto non-inferior solutions, and let x^m $(\mathrm{m} = 1,2,\dots,\mathrm{M})$ denote the set of these solutions. In the Pareto front, the slope at endpoints ($m = 1$ and M) are $k_1^{(1)}$, $k_2^{(2)}$, $k_1^{(M)}$, and $k_2^{(M)}$, which could be proposed as follows [57],

$$k_1^{(1)} = \frac{f_2^{(2)} - f_2^{(1)}}{f_1^{(2)} - f_1^{(1)}} \quad (8)$$

$$k_2^{(1)} = \frac{f_1^{(2)} - f_1^{(1)}}{f_2^{(2)} - f_2^{(1)}} \quad (9)$$

$$k_1^{(M)} = \frac{f_2^{(M)} - f_2^{(M-1)}}{f_1^{(M)} - f_1^{(M-1)}} \quad (10)$$

$$k_2^{(M)} = \frac{f_1^{(M)} - f_1^{(M-1)}}{f_2^{(M)} - f_2^{(M-1)}} \tag{11}$$

Step 3: Performing the sensitivity analysis between two average variabilities in a specific non-inferior Pareto solution. For this purpose, the sensitivity ratio is defined as $\delta_1^{(m)}$, which is proposed by a ratio of $k_1^{(m)}$ to f_1. Then, $\delta_2^{(m)}$ could be also considered as the ratio of $k_2^{(m)}$ to f_2. These formulations could be found as follows [57],

$$\delta_1^{(m)} = \frac{k_1^{(m)}}{f_1^{(m)}}, m = 1, 2, \dots, M, f_1^{(m)} \neq 0 \tag{12}$$

$$\delta_2^{(m)} = \frac{k_2^{(m)}}{f_2^{(m)}}, m = 1, 2, \dots, M, f_2^{(m)} \neq 0 \tag{13}$$

Step 4: Nondimensionalizing the sensitivity ratio for a better comparison. Supposing ε_1^m and ε_2^m are the results of δ_1^m and δ_2^m, respectively, the non-dimensionalization leads to [57],

$$\varepsilon_1^{(m)} = \frac{\delta_1^{(m)}}{\sum_{i=1}^{M} \delta_1^{(i)}}, m = 1, 2, \dots, M \tag{14}$$

$$\varepsilon_2^{(m)} = \frac{\delta_2^{(m)}}{\sum_{i=1}^{M} \delta_2^{(i)}}, m = 1, 2, \dots, M \tag{15}$$

Step 5: Considering two values for the solution ($x^{(i)}$ and $x^{(j)}$) in the Pareto front diagram, if $\varepsilon_l^{(i)} > \varepsilon_l^{(j)}$ $(l = 1, 2)$; then, $x^{(i)}$ dominates $x^{(j)}$ and $x^{(i)} > x^{(j)}$; else, $x^{(i)}$ becomes the non-dominant solution or non-inferior solution in the sensitivity analysis [57].

Step 6: Considering a bias degree for various objectives between the value of 0 and 1. Then, For M^* as the solution number in the domain of X^*, w_1^m and w_2^m are defined as [57],

$$w_1^m = \frac{\varepsilon_1^{(m)}}{\varepsilon_1^{(m)} + \varepsilon_2^{(m)}}, m \in M^* \tag{16}$$

$$w_2^m = \frac{\varepsilon_2^{(m)}}{\varepsilon_1^{(m)} + \varepsilon_2^{(m)}}, m \in M^* \tag{17}$$

where,

$$w_1^m + w_2^m = 1, m \in M^* \tag{18}$$

For objective functions of f_1 and f_2, $(w_1)_{max}$ and $(w_2)_{max}$ have a strong value of the bias degree [57].

Step 7: Using Formulation (19), $\Delta\varepsilon^{(m)}$ (m = 1, 2, …, M) could be considered as [57],

$$\Delta\varepsilon^{(m)} = \left|\varepsilon_1^{(m)} - \varepsilon_2^{(m)}\right|, m = 1, 2, \dots, M \tag{19}$$

Step 8: Lowering $\Delta\varepsilon^{(m)}$ to obtain $(\Delta\varepsilon)_{min}$, which is the best unbiased solution. Notably, in this condition, the values of f_1 and f_2 are acceptable [57].

$$(\Delta\varepsilon)_{min} = \min\{\Delta\varepsilon^{(1)}, \Delta\varepsilon^{(2)}, \dots, \Delta\varepsilon^{(M)}\} \tag{20}$$

Step 9: End.

2-6) 3D Printing

Printing some regions of a part before starting the main study is a method of optimally manufacturing specimens, thereby reducing the financial and time loss caused by the trial-and-error stage. The selected patterns, including honeycomb, rectangular, triangular, regular octagon, and irregular octagon with 60% infill were printed along with rectangular patterns

with 40% and 80% of filling densities were printed as shown in Fig 4. The green lines in Fig 4 represent the desired part of the print samples that was cut and evaluated through inspection in the FESEM.

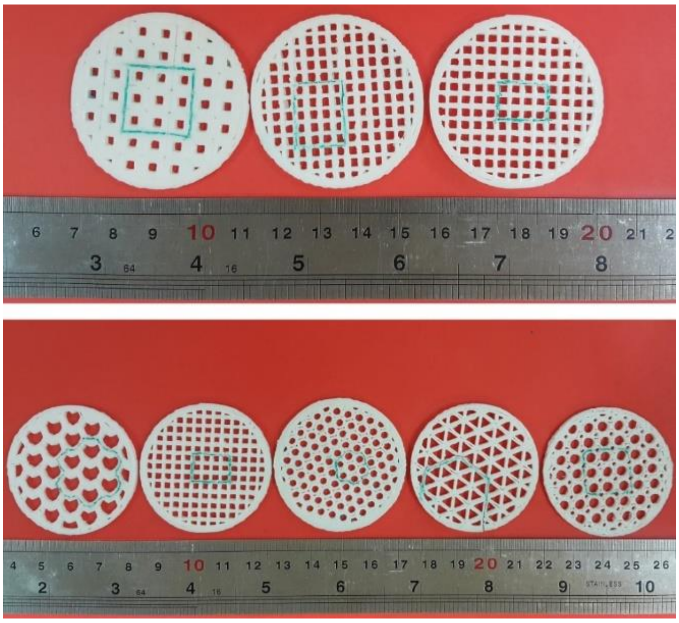

Fig 4: The geometry of the 3D-printed specimens with different filling patterns and infill densities

In order to provide the specimens, an FDM 3D printer was developed in the research laboratory of advanced materials behavior (AMB), as shown in Fig 5. Table 6 shows the conditions under which all of the samples were printed. To ensure optimal results and utilize the capabilities of an industrial-duty 3D-printer machine, the samples were printed at a scale of 5 times larger than the original CAD models. One other reason is due to the machine limitations. This approach allowed to produce lattice structures with circular sections and a diameter of 45 millimeters, which would have been challenging to achieve with smaller scales. While this approach allowed for the successful printing of the specimens, it is important to investigate the potential effects of scaling on the accuracy of the geometrical features and the resulting mechanical properties in future investigations. Previous studies demonstrated clear size effects on the mechanical response of additively manufactured lattice structures [58]. 3D printing was done with white-colored PLA filaments, which diameter was 1.75 mm, by the YouSu Company. The samples were printed with 100% infill and no top and bottom solid layers printed, allowing for clear visualization of the print path.

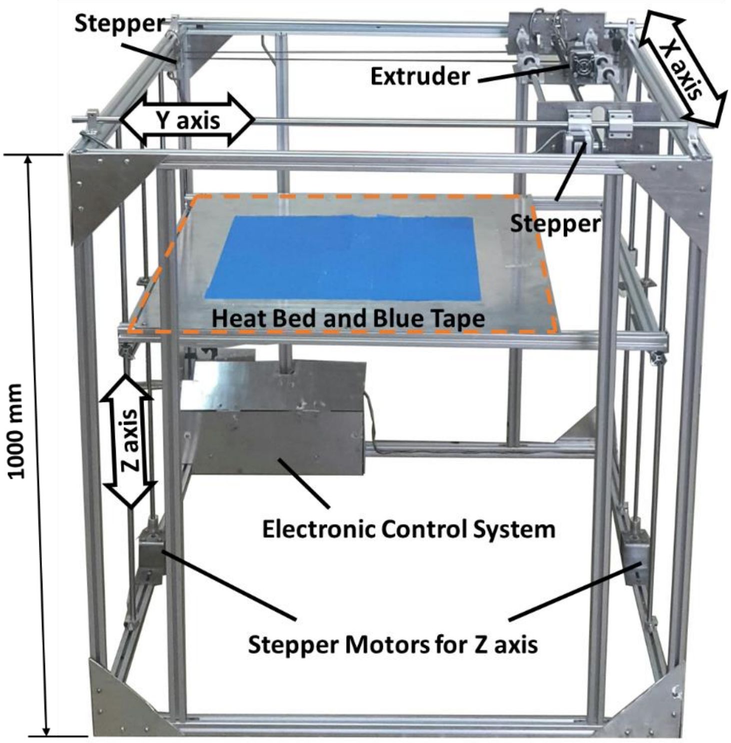

Fig 5: The FDM 3D printer used for additive manufacturing of the specimens

Table 6: The parameters of 3D printing [56, 59- 60]

Process variable	Operating condition
Nozzle diameter (mm)	0.40
Filament diameter (mm)	1.75
Layer height (mm)	0.20
Perimeter	2
Top and bottom solid layers	0
Print speed (mm/s)	10
Travel speed (mm/s)	30
Nozzle temperature (°C)	210 (First layer)<br>180 (Entire layers)
Bed temperature (°C)	25 (Room temperature)

2-7) Inspections

A close examination of the samples was performed after printing. For this purpose, a conductive golden layer was applied on the samples by gold sputtering and inspected using a TESCAN (MIRA 3 LMU) FESEM. Imaging was performed with magnifications of different magnitudes. The length of the vertices and width of lines were measured in the microscopic images to understand the accuracy of prints.

3) Results and Discussion

3-1) Stress Analysis

Fill pattern and filling density are critical conditions for the fabrication of AM components. To investigate the influence of these parameters on the performance of printed parts, specimens

with different filling patterns and densities were modeled in ABAQUS software. Due to the great dependence of the stress analysis results on boundary conditions and loadings, especially the maximum stress, the constraints must be carefully set. Based on the results from stress analysis, the maximum stress occurs in the clamped region. As expected, there is a tensile state of axial stress at the top of the beam and a compressive axial stress state at the bottom. Thin walls between the patterns cause stress concentration, so the lower filling density cause higher stress. But the filling pattern affects differently in each filling percentage. As shown in Fig 6(a), honeycomb samples had higher stress in 40% and 80% infill, whereas in the case of irregular octagons, maximum stress occurred in 60% infill. However, irregular octagon, rectangular and regular octagon had the lowest stresses in 40%, 60%, and 80% of infill, respectively.

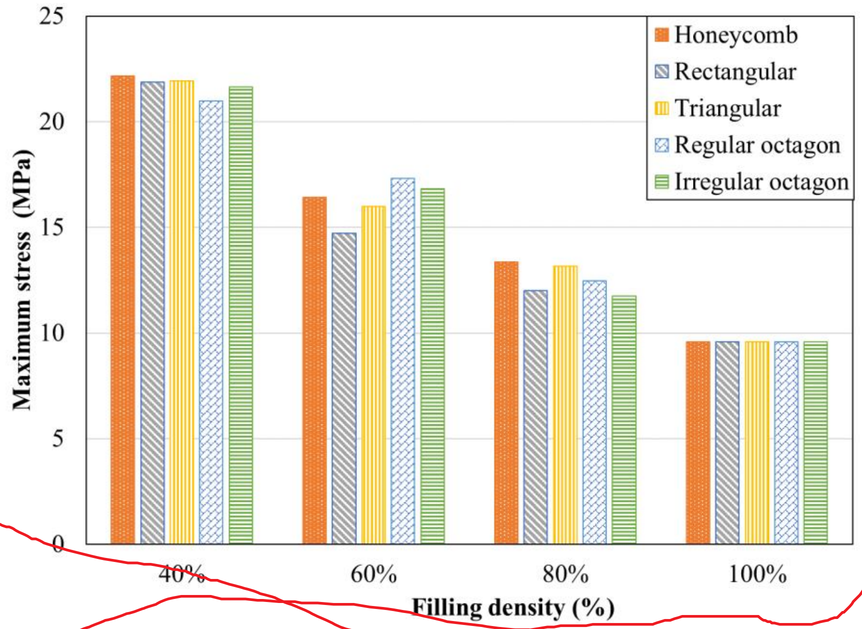

(a)

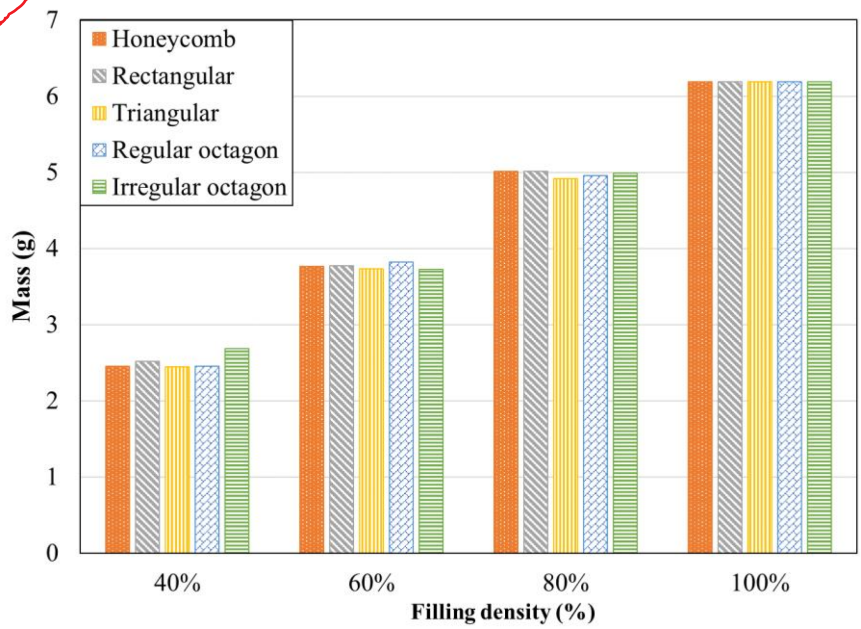

(b)

Fig 6: The influence of filling parameters on (a) maximum bending stress and (b) mass

The comparison of weight reduction in this study is conducted by considering the calculated weight values, taking into account the theoretical density of the material and the measured volumes of the samples in the CAD model. By utilizing these parameters, the weight of the objects can be estimated. Based on the results shown in Fig 6(b), the mass of the honeycomb and rectangular lattice structures was nearly identical at various infill densities, with the rectangular pattern being slightly heavier at 40% infill. In addition, the triangular pattern exhibited less mass compared to the other three patterns. The irregular octagon, regular octagon, and rectangular patterns were the heaviest at 40%, 60%, and 80% infills, respectively. However, it is clear that by decreasing filling density, specimens get lighter, and the triangular pattern has the lowest mass in all cases. The stress distribution contours obtained from finite element simulation are summarized in Table 7 and 8. As shown in stress distribution contours, the bending load generates compressive stress on the bottom side of the clamped end and tensile stress on its top side. However, in fatigue testing, the load is cyclic and changes the loading direction periodically, which leads to alternating regions of compressive and tensile stresses. Therefore, the specimens undergo both compressive and tensile stresses during fatigue testing, which can affect their durability and mechanical performance. Although the stress distribution in PLA specimens under cyclic loading cannot be measured experimentally, the fatigue behavior of the material is still worth investigating in future researches. This information can be compared to numerical results.

Table 7: The von-Mises stress distribution contours for different patterns

Pattern	40%	60%	80%
Honeycomb	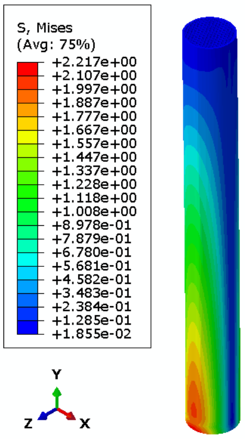	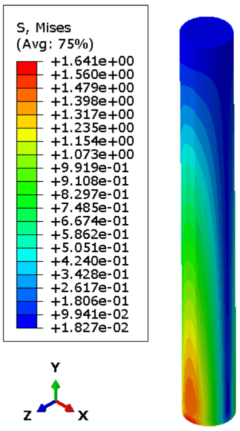	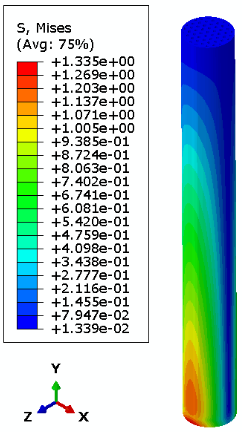
Rectangular	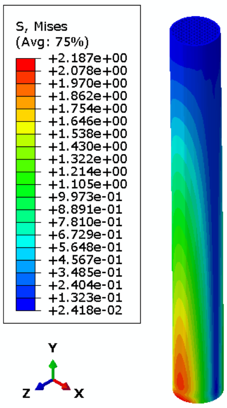	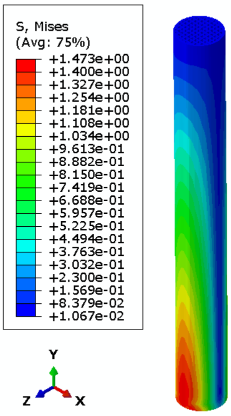	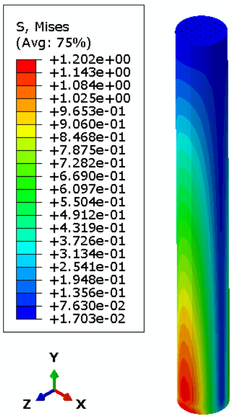

Triangular

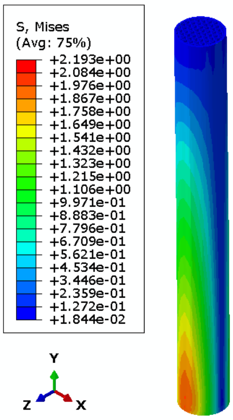

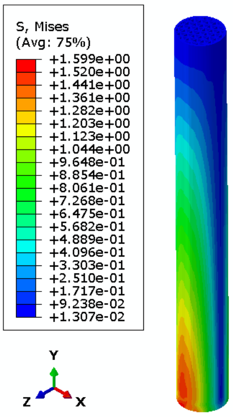

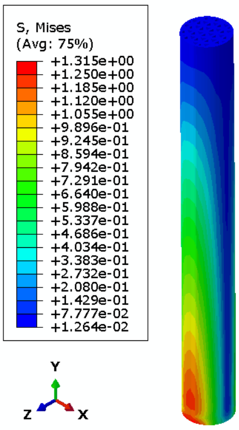

Regular octagon

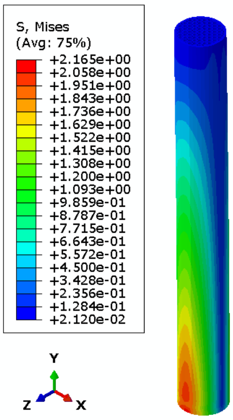

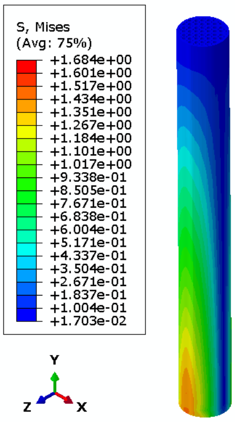

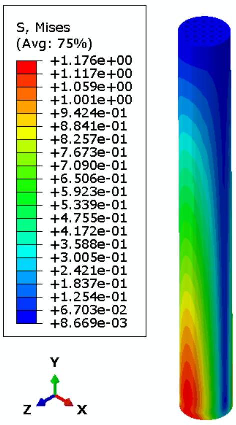

Irregular octagon

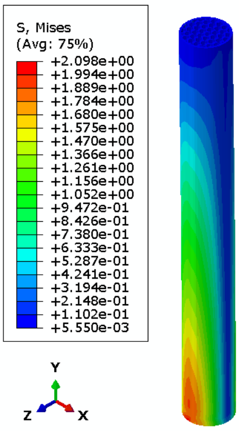

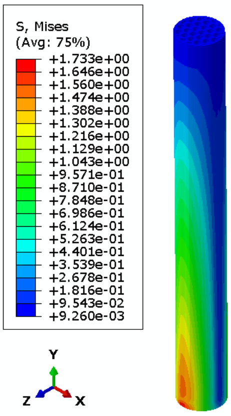

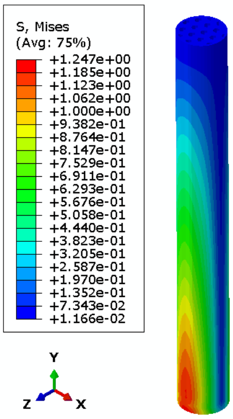

Full filled (100%)

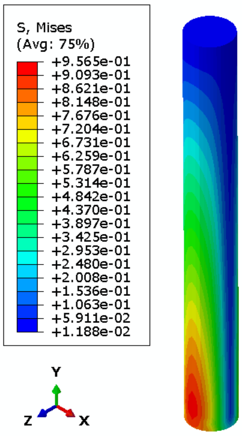

Table 8: The stress distribution contours in Y direction (σ_{yy}) for different patterns

Pattern	40%	60%	80%
Honeycomb	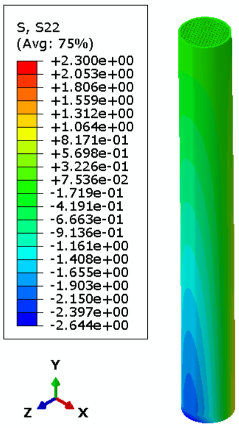	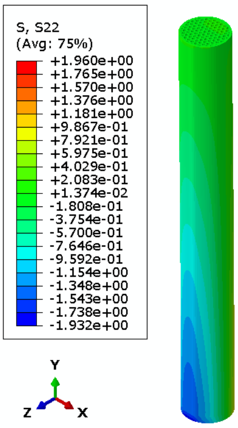	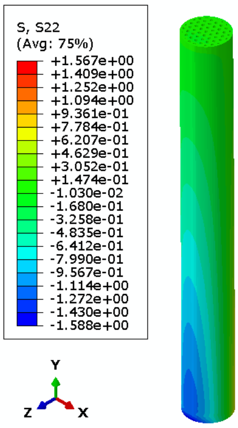
Rectangular	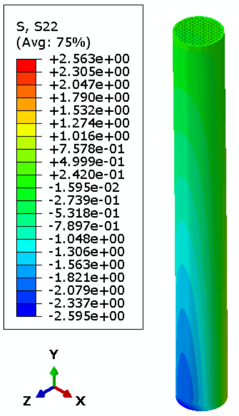	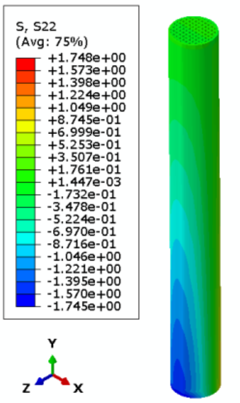	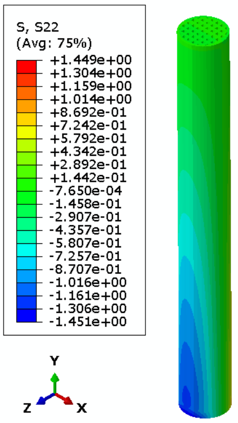
Triangular	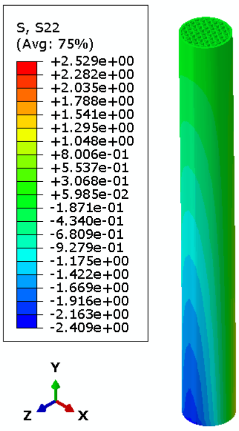	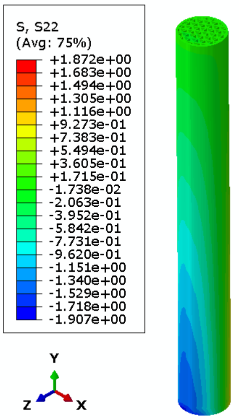	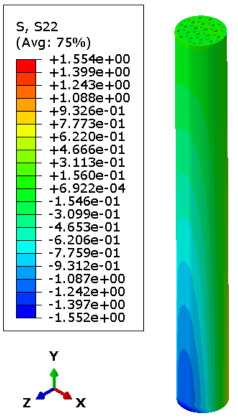
Regular octagon	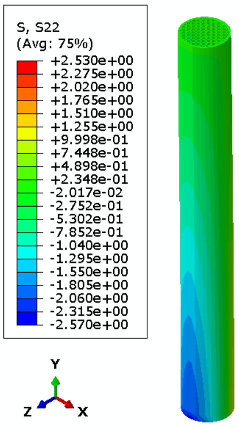	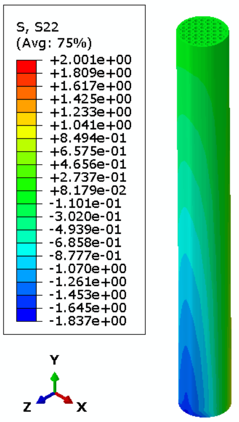	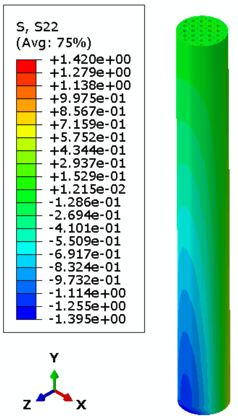

<table>
<tr>
<td>Irregular octagon</td>
<td>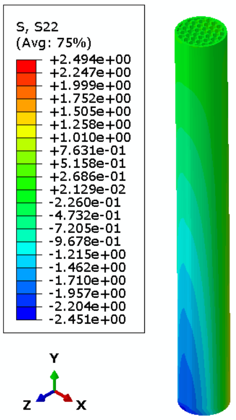
</td>
<td>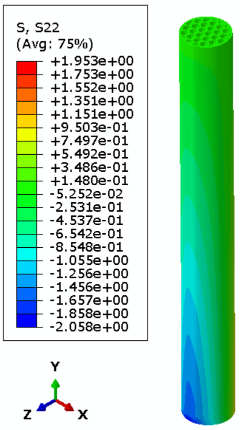
</td>
<td>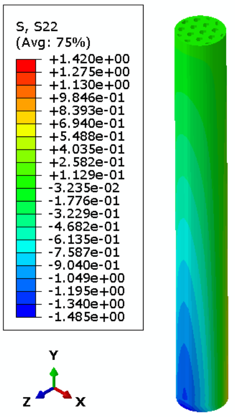
</td>
</tr>
<tr>
<td>Full filled (100%)</td>
<td colspan="3">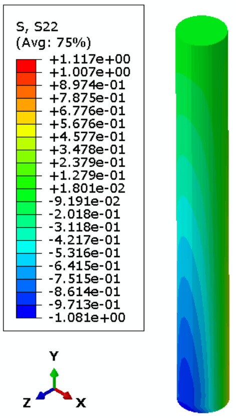
</td>
</tr>
</table>

3-2) Two-Objective Optimization

To find the optimized filling parameters, a multi-objective optimization method proposed by Wang et al. [57], was used. First, based on the objective function proposed in Table 5, the distribution of the Pareto non-inferior solution set was obtained for each set. Using formulations (6)-(13), the sensitivity analysis on the non-inferior solutions of f_1 and f_2 was calculated. Then, based on Formulations (14) and (15), the sensitivity ratio of f_1 and f_2 was ~~nondimensionalized~~. Formulations (16) and (17) were considered to find w_1 (for f_1) and w_2 (for f_2). Based on the dominance relationship of the bias degree of the 15 Pareto non-inferior solutions from each set of objective functions, eight solutions have been ruled out. The remaining solutions form the subset of non-inferior solutions based on the bias degree. Using Formulation (19), the values of Δε are obtained for each set of objective functions and presented in Tables 9 and 10 for horizontal and vertical print directions, respectively. The index of Δε in these tables indicates the corresponding objective function number. Therefore, $(\Delta\varepsilon)_1$, $(\Delta\varepsilon)_2$, and $(\Delta\varepsilon)_3$ are related to the first, second, and third set of objective functions. The minimum value of this parameter, $(\Delta\varepsilon)_{min}$, can be the superior unbiased solution, which could be acceptable for f_1 and f_2.

In the case of vertical print direction, the values of Δε are listed in Table 9. The solutions of 5th, 6th, 7th, 8th, 9th, 10th, 13th, and 15th from Set 1, 5th, 6th, 7th, 8th, 9th, 10th, 13th, and 14th from Set 2 and 4th, 5th, 6th, 9th, 10th, 11th, 14th, and 15th from Set 3 are dominated and therefore, they should be ruled out. Hence, the solution of 14th, 11th, and 13th are good results for vertical print direction and acceptable for both objectives in the first, second, and third set of objective functions, respectively. As it is obvious, by selecting different objective functions, the results will be different.

Table 9: Δε corresponding to Pareto non-inferior solutions for vertical print direction

No.	Filling density	Fill pattern	$(\Delta\varepsilon)_1$	$(\Delta\varepsilon)_2$	$(\Delta\varepsilon)_3$
1	40	Honeycomb	0.1095	0.0902	0.4380
2	40	Rectangular	0.3254	0.2972	0.4344
3	40	Triangular	0.3342	0.3029	0.4474
4	40	Irregular octagon	0.1166	0.1183	0.4177
5	40	Regular octagon	0.1085	0.0956	0.4645
6	60	Honeycomb	0.5889	0.1199	0.4561
7	60	Rectangular	0.6461	0.1046	2.3375
8	60	Triangular	0.4181	0.0665	2.3249
9	60	Irregular octagon	0.3591	0.0696	0.4842
10	60	Regular octagon	0.0293	0.0322	0.3795
11	80	Honeycomb	1.4354	0.6114	0.3521
12	80	Rectangular	1.3944	0.6027	0.0628
13	80	Triangular	0.0903	0.0394	0.0073
14	80	Irregular octagon	0.0735	0.0295	0.3310
15	80	Regular octagon	0.2226	0.0927	0.3872

Table 10: Δε corresponding to Pareto non-inferior solutions for horizontal print direction

No.	Filling density	Fill pattern	$(\Delta\varepsilon)_1$	$(\Delta\varepsilon)_2$	$(\Delta\varepsilon)_3$
1	40	Honeycomb	0.1092	0.0893	0.0907
2	40	Rectangular	0.3264	0.2948	0.0461
3	40	Triangular	0.3353	0.3000	0.0281
4	40	Irregular octagon	0.1152	0.1178	0.0469
5	40	Regular octagon	0.1066	0.0940	0.0668
6	60	Honeycomb	0.5302	0.1176	0.0129
7	60	Rectangular	0.5815	0.1004	0.1055
8	60	Triangular	0.3729	0.0633	0.0934
9	60	Irregular octagon	0.3198	0.0670	0.0116
10	60	Regular octagon	0.0265	0.0328	0.0980
11	80	Honeycomb	1.3343	0.6003	0.0484
12	80	Rectangular	1.2958	0.5945	0.0632
13	80	Triangular	0.0839	0.0400	0.0390
14	80	Irregular octagon	0.0691	0.0289	0.0497
15	80	Regular octagon	0.2083	0.0932	0.0756

On the other hand, for longitudinal print direction, according to the data listed in Table 10, the 1st, 5th, 6th, 7th, 8th, 9th, 10th, and 13th solutions from Set 1 are dominated. The 1st, 6th, 7th, 8th, 9th, 10th, 13th, and 14th solutions should be ruled out from Set 2 based on the dominance relationship. The 1st, 2nd, 5th, 7th, 8th, 11th, and 15th solutions are also the remaining solutions from Set 3. Therefore, the 14th, 15th, and 2nd solutions meet equation 20 and are determined to be the unbiased and good solutions in the case of horizontal print direction for Sets 1, 2, and 3, respectively. Notably, the quantized values of the bias degree for objectives are also calculated.

The decision can be balanced and selected more efficiently according to the quantitative indicator. For example, if objective f_1 is preferred in the third set of objectives, the 7th non-inferior solution should be selected as the bias degree of the solution, which reached 0.8112

for f_1 and it is only 0.1888 for f_2. Moreover, if the objective of f_2 is preferred, the 1st non-inferior solution is selected as the bias degree of the solution reaches 0.7742 for f_2 and it is only 0.2258 for f_1.

In Fig 7, the Pareto front indicates that there was not much change in the fatigue lifetime of vertical specimens (green points) even with a severe reduction in mass. On the other hand, the horizontal orientation (blue points) resulted in layer lines being placed horizontally in the direction of the bending force, making it the optimal orientation for mechanical strength. Despite the need for support during printing, this orientation provides the best mechanical strength. This result is also obtained in the literature [56,61], so horizontal print direction is selected in this study.

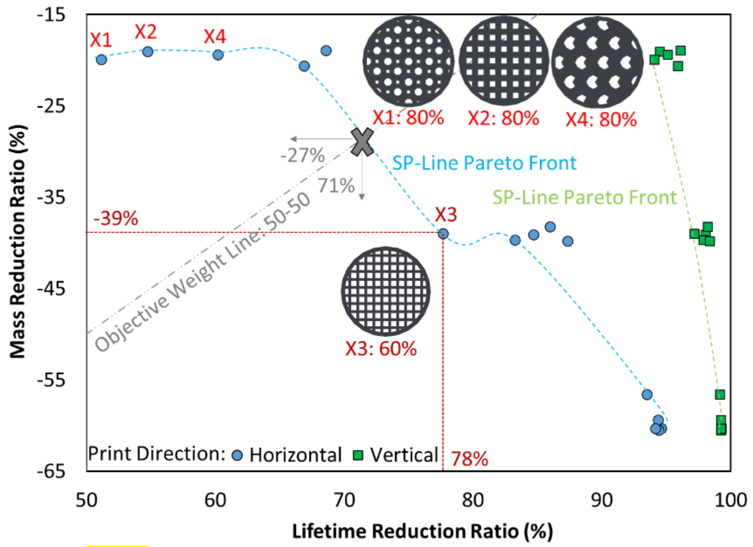

Fig 7: The Pareto front for filling parameters

In Fig 7, the point at which the 50-50 line crosses the Pareto front indicates a state of a 50% mass reduction and a 50% increase in the lifetime compared to the solid sample. According to the importance of mass in biomechanical applications, the rectangular pattern with 60% filling can be a good choice among the two samples that are closest to the point where the 50-50 line meets the Pareto front. Considering the interaction of objective functions, in the rectangular pattern with 60% of the filling density, the fatigue life has decreased by only 22%, despite the 39% reduction in mass. In addition, this sample has the shortest distance to the optimal point in the results of two-objective optimization by selecting the third set of objective functions (in Table 5) with an emphasis on the second objective. Based on this, fatigue samples should be printed with a 60% rectangular pattern.

The obtained results in the literature have also illustrated that the infill pattern through 3D printing influenced the mechanical responses of AM structures. For example, the triangular infill pattern with 40% infill density in PLA achieved a maximum peak force in low-velocity impact testing compared to the quarter cubic, grid, and tri-hexagon patterns [62]. The optimal filling pattern for the nylon matrix with a reinforcement under the uniaxial tension-tension fatigue test was triangular, with 20% of the filling percentage [63]. Moreover, the triangular shape had a superior tensile strength in the composites, reinforced with continuous carbon fibers and chopped carbon fibers (Onyx) [64]. The rectilinear pattern presented the best mechanical performance, having a high elastic module, tensile strength, and impact resistance, compared to the grid, honeycomb, and triangle [65]. The specimens manufactured

through a filling pattern of honeycomb with 75% of the filling density could be preferred to the rectilinear samples to increase the fatigue lifetime of PLA-wood composite structures [52].
Vigneshwaran and Venkateshwaran [66] found that changing the filling percentage (in naturally available wood flour reinforced with 30-90% of biodegradable PLA) multiplied the tensile performance and also the stiffness by factors of 2.0 and 1.5, respectively for wood-PLA bio-composites. According to their results, the printing pattern, whether hexagonal, rectangular, or triangular did not find to affect the final characteristics of bio-composites. The rectangular-filled nylon structure illustrated the maximum value for tensile properties compared to triangular- and hexagonal-filled components. On the other hand, the triangular-filled part had the minimum value of tensile properties, while the hexagonal-filled sample demonstrated a lower value for tensile performance than the rectangular-filled part [67]. The highest value of strength and lowest value of deformation are related to the irregular octagon structure and under 10 N compression load compared to the regular octagon under 10 N compression load [51]. As a result, depending on the material and the conditions of use, the performance of filling parameters is different and should be considered in the design domain.

3-3) Numerical Results and Optimization

The topology optimization procedure was done in ABAQUS with the SIMP technique. Fig 8 shows the resulting plot of this process. According to this figure, a mass of 1.1767 g and a total strain energy of 0.056 J were achieved after 50 cycles. As a result of the optimization efforts, the desired optimized geometry was obtained. A threshold of the ISO value is generally used for regulating, which the finite elements are displayed. This value is utilized to obtain the positions on the finite element edges, where the new nodes are made and must be between zero and unity. Larger values lead to a model with a smaller volume. The determined design is depicted in Fig 9. Notably, it should emphasize that the proposed geometry only demonstrates the elements with a density higher than 20% to maintain the continuity of components.

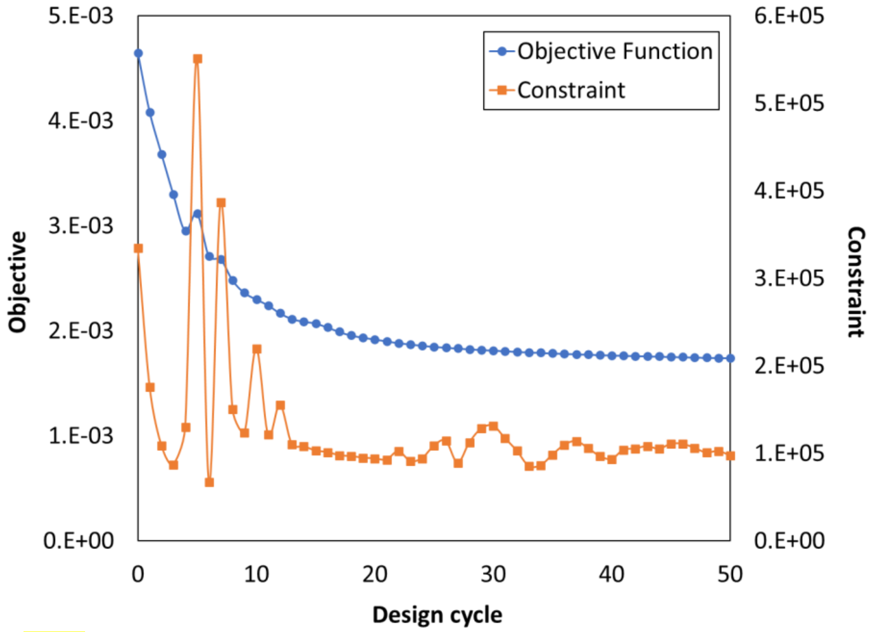

Fig 8: The variations of design responses through design cycles

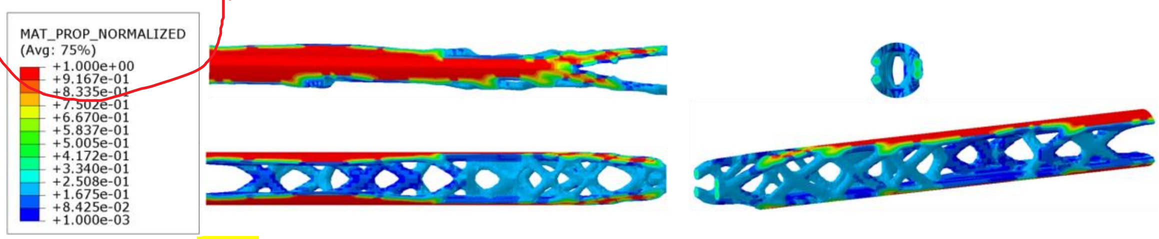

Fig 9: The extracted geometry from topology optimization

The optimized geometry in Fig 9 was reached after 50 design cycles. An animation is also included in supporting information (Fig S1) that shows the topology optimization process developments during each design cycle, providing a visual representation of the design process. Such visual support presents a better understanding of the design evolution and the optimization steps taken to arrive at the final design. The optimized geometry has a unique characteristic in its design - a quadruple thread-like pattern surrounded by a shell. This pattern is evident throughout the structure and adds a distinct visual aspect to the overall design. The quadruple thread-like pattern with its shell provides an optimized structure with improved mechanical performance by reducing mass and enhancing strength in specific locations. The literature supports that the outer shell affects bending strength. Samples with ABS: TPU: ABS sandwiched structure showed higher flexural strength due to ABS material in outer layers, aiding compression and expansion during loading [5]. The thread-like pattern serves as a unique solution in topology optimization and can be customized to meet specific design requirements. Accordingly, the design realization step was performed for further considerations of manufacturability. This could be a crucial step in topology optimization since it is the stage where the optimized design is translated into a manufacturable and practical product. In this step, the abstract and optimized design obtained from the topology optimization process is transformed into a tangible, functional product that can be manufactured. The design realization step involves several important tasks, including geometry clean-up, and preparation of the final design for manufacturing. In this step, the design must be refined to ensure that it meets all design requirements and constraints and can be manufactured using the desired materials and processes. The realized design, which is presented in Fig 10, is believed to be conceptually feasible due to rotational symmetry. This design approach has potential applications in various industries, such as biomedical to produce stents, where reducing mass and increasing strength are critical considerations. The printed specimen in Fig 11 exhibits the desired geometry and structural features as intended in this design. This physical realization served as a tangible representation of the proposed model and validated the effectiveness of the present design process. It provided concrete evidence that the optimized design can be successfully translated into a physical object through 3D printing.

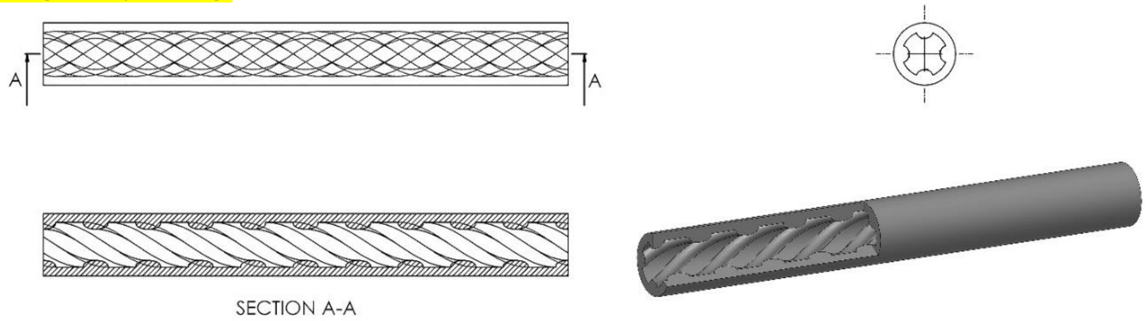

Fig 10: The geometry of the designed sample based on the result of topology optimization

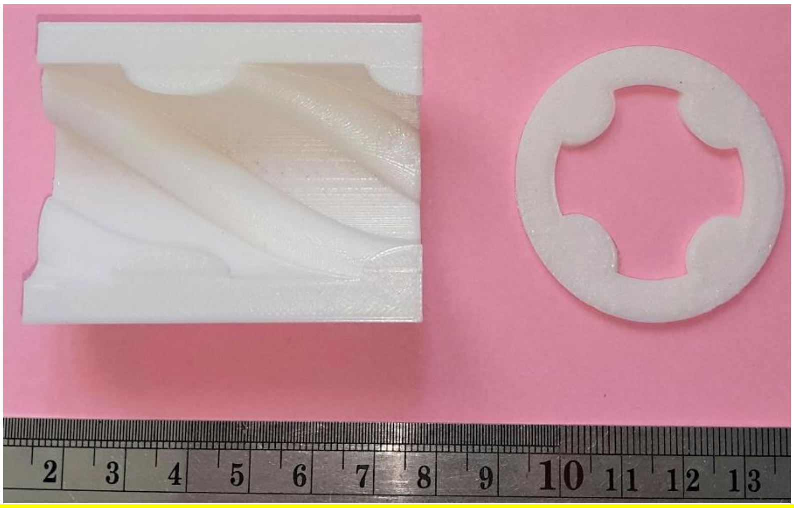

Fig 11: The designed sample printed based on the result of topology optimization

Fig 12 and 13 present the results of the static analysis of the optimized components. Fig 12 presents three geometries after (a) topology optimization, (b) shape optimization, and (c) design realization steps. The figure provides a visual depiction of how the optimization process affected the stress distribution of the component and how the optimized design was implemented in the final product. The maximum von-Mises stress was 97.08 MPa, which was observed after the topology optimization and located at the point shown in Fig 12(a). As it can be seen, the obtained geometry proposed several sharp edges and also some irregular surfaces. This issue is related to mesh refinement under boundary conditions.

Thus, a post process should be done based on smoothing and repairing the previous operations on such a sharp surface, by a shape optimization in order to minimize the von-Mises stress. As a result, maximum stress homogenized and decreased to 44.30 MPa after 15 design cycles (Fig 12(b)). The geometry after the design realization step is the final product of the optimization process. This geometry represents the optimized component that has been manufactured using 3D printing technology. The optimized component was designed based on the results of the multi-objective optimization, which took into consideration factors fatigue lifetime and mass. Despite the increase in mass compared to the topology optimized sample, the maximum von-Mises stress greatly reduced, based on Fig 12(c). With a mass of 3.56 g, this sample is lighter than the samples with 60% of filling density. However, due to less stress, it tolerates more cycles. The design realization step is the final step in the optimization process and the optimized component is now ready to be used in applications that require its unique properties. Figure 13 displays the stress distribution along the component axis in optimized geometries. The contour lines in the figure accurately represent the stress distribution, including stress concentration regions, mirroring the pattern observed in the von-Mises stress. Additionally, the figure distinctly reveals areas with compressive stresses.

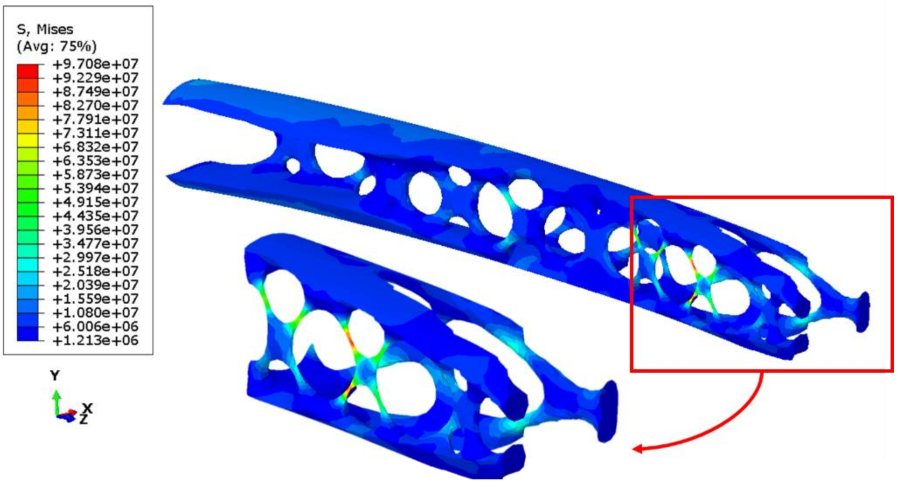

(a)

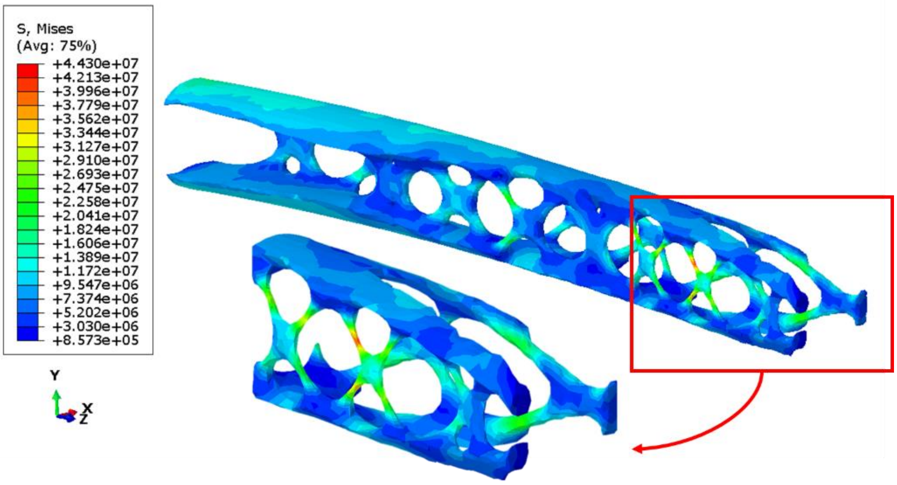

(b)

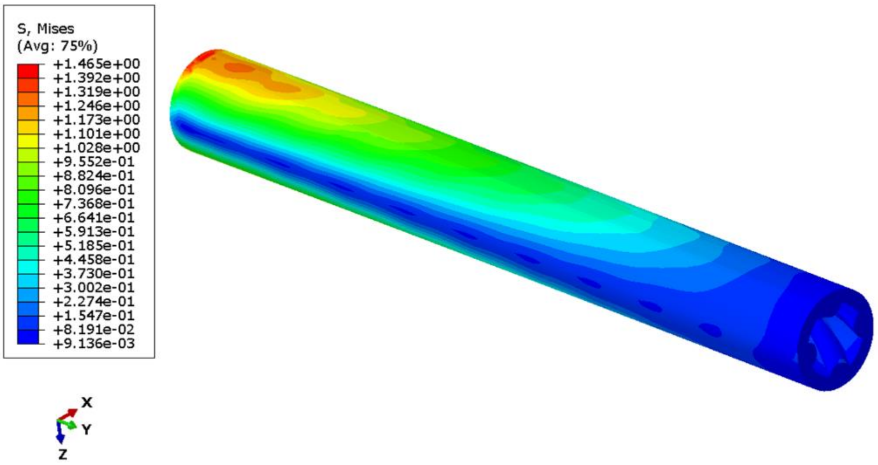

(c)

Fig 12: The von-Mises Stress distribution in the optimized component after (a) topology optimization and (b) shape optimization (c) design realization

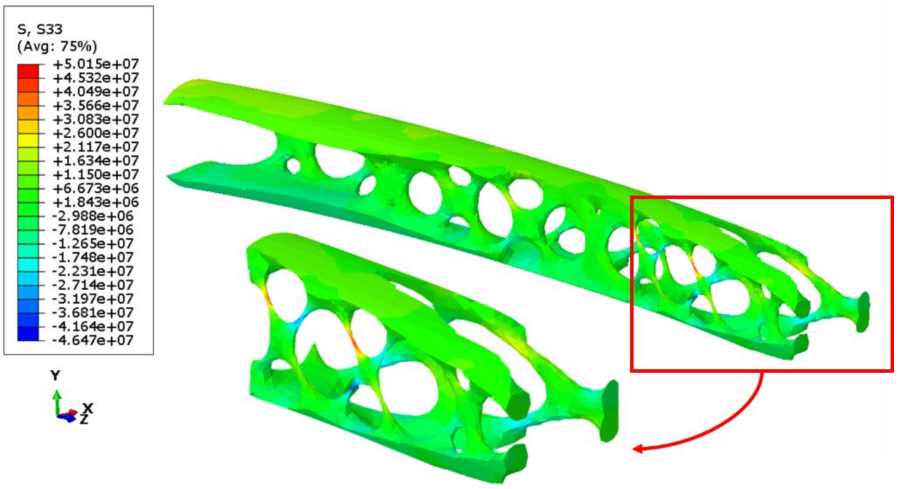

(a)

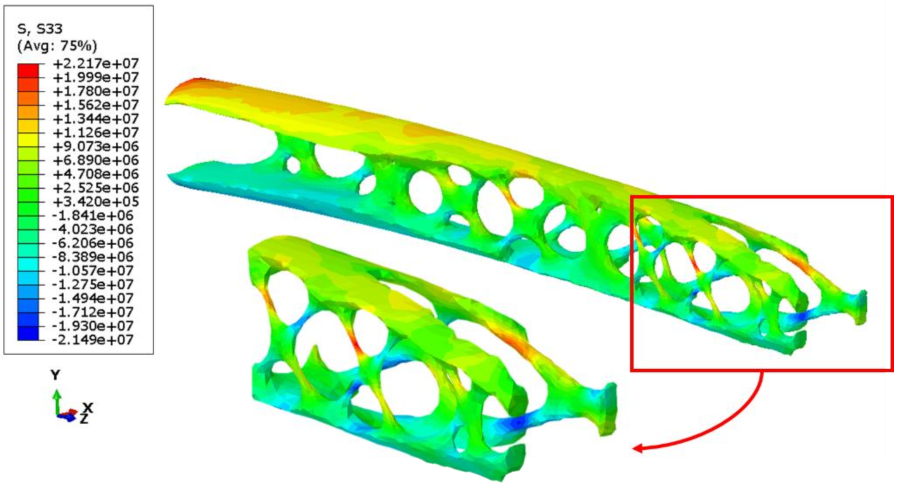

(b)

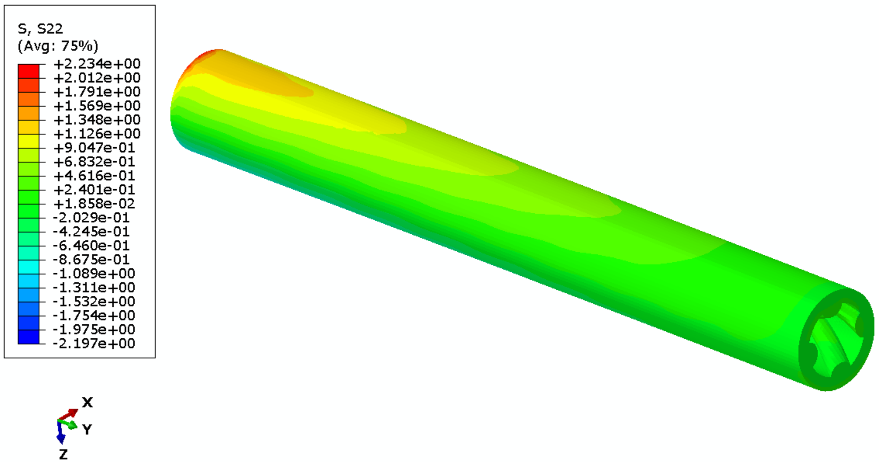

(c)

Fig 13: Stress distribution in the direction of cylinder axis in the optimized component after (a) topology optimization and (b) shape optimization (c) design realization

Table 11 compares the results obtained from the various optimization processes. This table provides a quantitative evaluation of the impact of the optimization process on the mass of components, maximum von-Mises stress and fatigue lifetime based on the printing direction. The comparison allows the assess the effectiveness of the optimization process and draw

conclusions about the optimal values. This information can be useful for engineers and designers in future 3D printing projects.

Table 11: The comparison of the results through the optimization processes

Optimization process	Mass (gr)	Maximum von-Mises stress (MPa)	Fatigue lifetime (cycle)	
			Horizontal	Vertical
Topology	1.18	97.08	37	6
Shape	1.18	44.30	564	85
Realized design	3.56	1.46	80424865	7050142

3-4) Morphological Characteristics

The desired part of the print samples, which are marked with green lines in Fig 4, has been cut, evaluated by inspection in the FESEM, and then examined for dimensional and geometrical accuracies. In order to assure adequate electrical conductivity for FESEM observation, samples were coated with a golden layer. The imaging process was performed with different magnifications. The printed samples had acceptable overall compliance of the shape with designed geometries, according to low magnification images (Fig 14). However, it is also obvious that the regularity of the rectangular pattern was the best, while the regular octagon pattern was the most irregular. Easy to be noticed by the eye, the line spacing is significant in the honeycomb pattern. In addition, with higher fill percentages, the spacing between lines decreases.

As illustrated in Fig 15, voids could be observed at the turning points of the extruder. According to previous studies, this void enhances by increasing the temperature of the extrusion. An incorrect control of the temperature through filament depositing can affect the adhesion between 3D-printed layers and also the filament sides along the filling pattern through the raster (Fig 16(a)) [68]. In other words, the air gap between the rasters enhances by increasing the deposition temperature. Notably, the raster width has a higher value at lower temperatures and therefore, it effectively reduces by enhancing the deposition temperature [69].

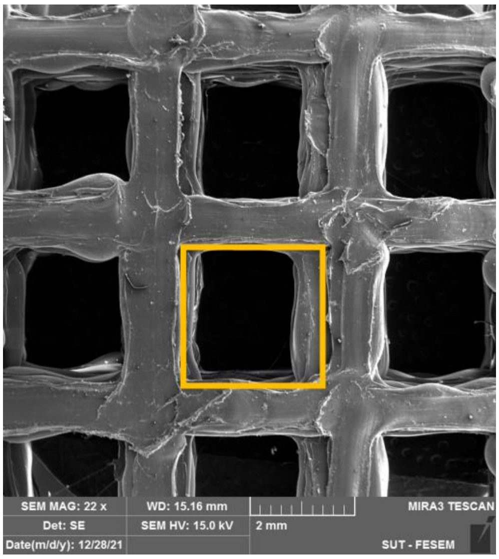

Rectangular (40%)

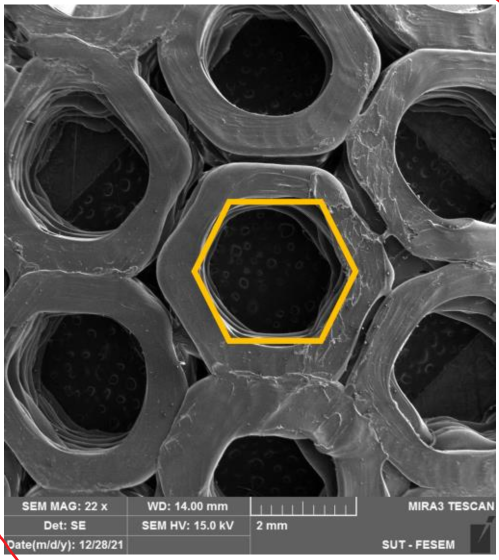

Honeycomb

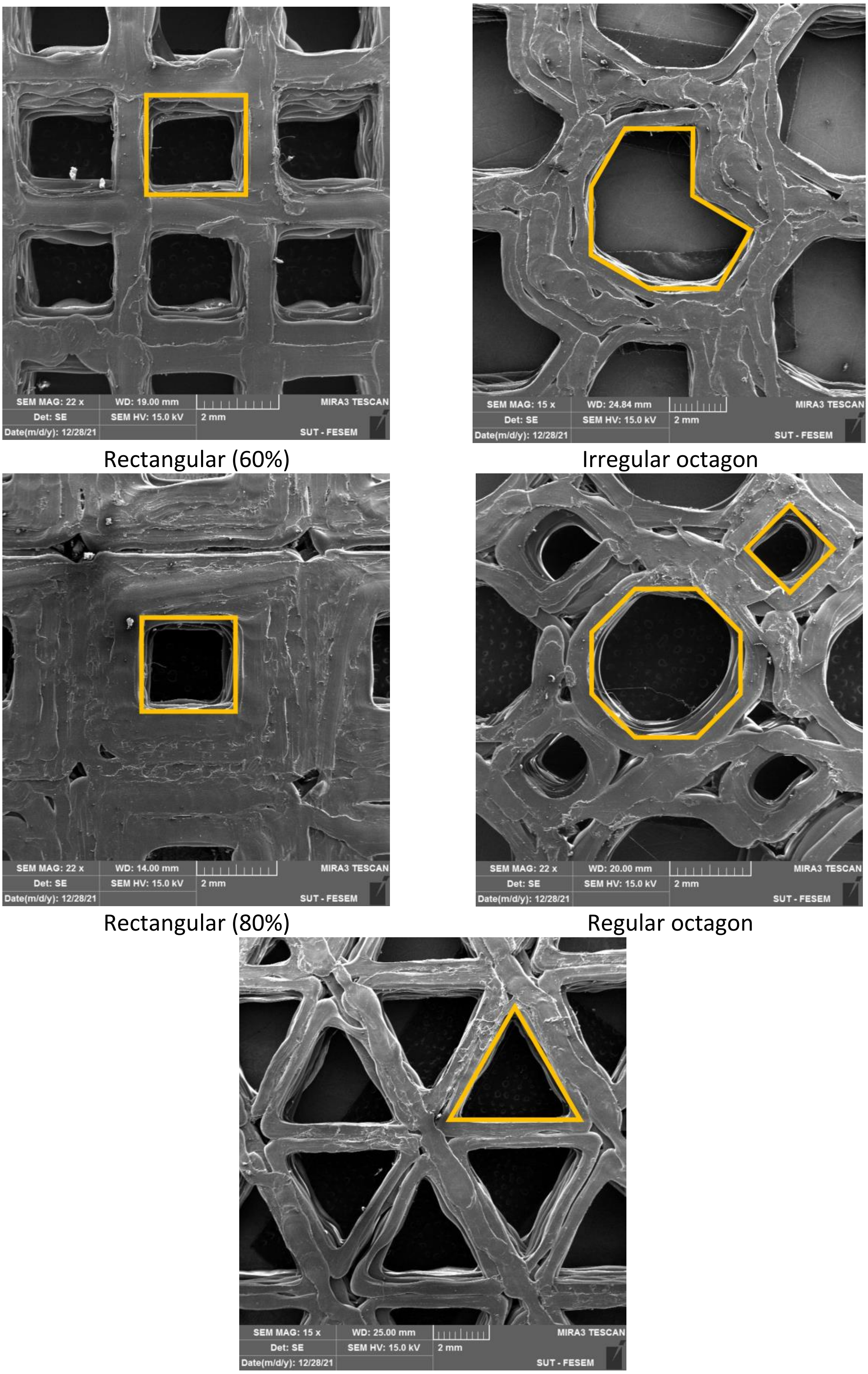

Fig 14: The low-magnification FESEM images of the printed samples

As observed in Fig 16(b), some small spherical-shaped defects may be generated during the fabrication of the filament, which can negatively impact the mechanical properties. Additionally, the extrusion feed rate plays a critical role in determining the quality of printed parts, as shown in Fig 16(c). If the extrusion feed rate is not balanced with the extrusion temperature and printing speed, inconsistent fusion can occur, resulting in defects. Another parameter that can affect the quality of printed parts is the distance between the nozzle and the build platform [70]. This distance can impact the adhesion between the layers and can cause defects similar to those shown in Fig 16(c). It is essential to consider these factors during the design and optimization of 3D-printed parts to achieve the desired mechanical properties and avoid potential defects.

Based on the microscopic images, different kinds of voids can significantly impact the quality and properties of 3D-printed parts, and the infill patterns examined in this study is one such example [56]. The infill patterns, often referred to as meta-structures, play a crucial role in influencing the properties of the components. Additionally, there are other forms of anisotropy that can arise from changes in printing paths or inadequate print parameters. While these factors were not specifically investigated in this study, it was reported from the porosity analysis that the adhesion between the laminated layers has no significant role to play in flexural properties [6].

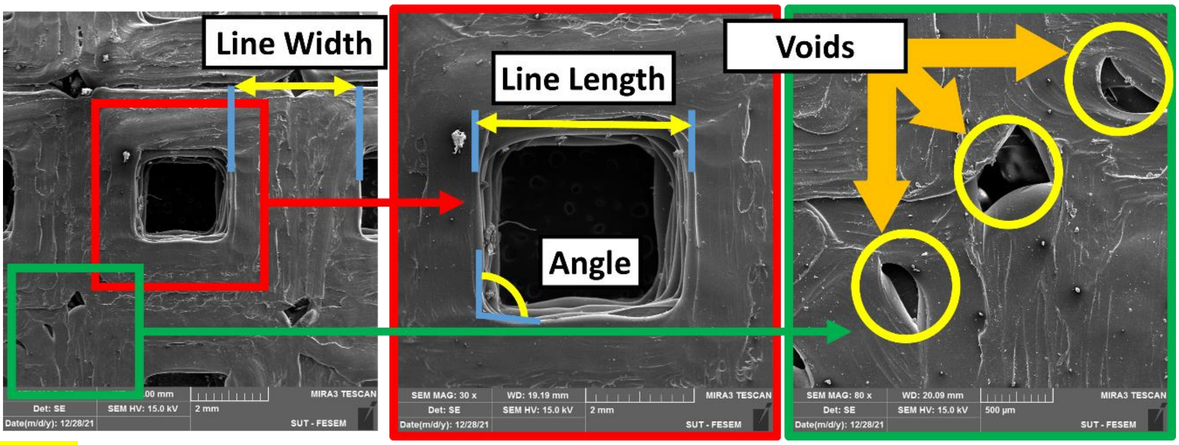

Fig 15: The FESEM micrographs of the 3D-printed PLA with rectangular pattern and 80% of infill at different resolutions

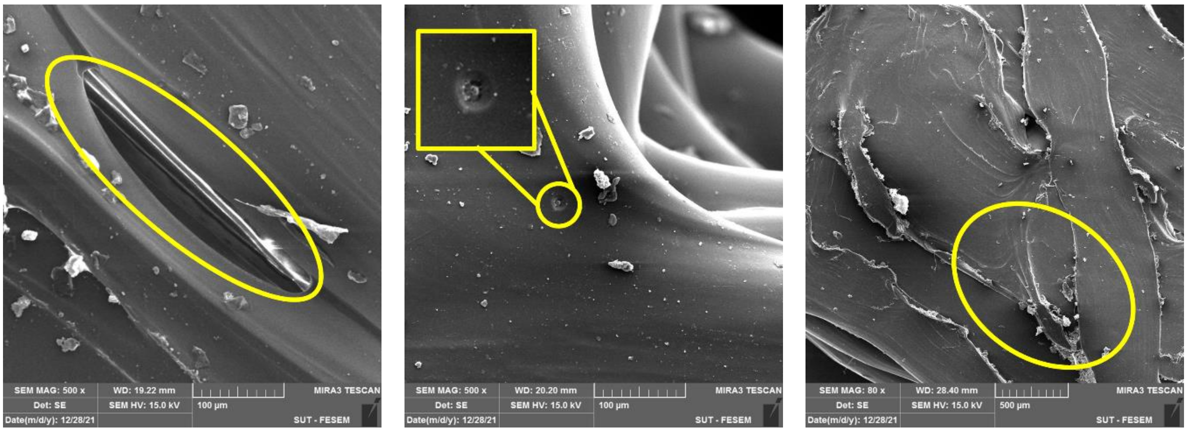

(a) (b) (c)

Fig 16: The defects observed in the samples with (a) 60% infill regular octagon, (b) 80% infill rectangular, and (c) 60% infill irregular octagon

As illustrated in Figs 15 and 17, the length of the vertices and width of lines were measured in the microscopic images to visualize dimensional accuracy. Specifically, Fig 15 shows a schematic of the measured line width and line length.

The measured line width in this picture does not refer to the actual line width produced by the nozzle. Rather, it is the width of the walls in the structure, which may consist of multiple print paths.

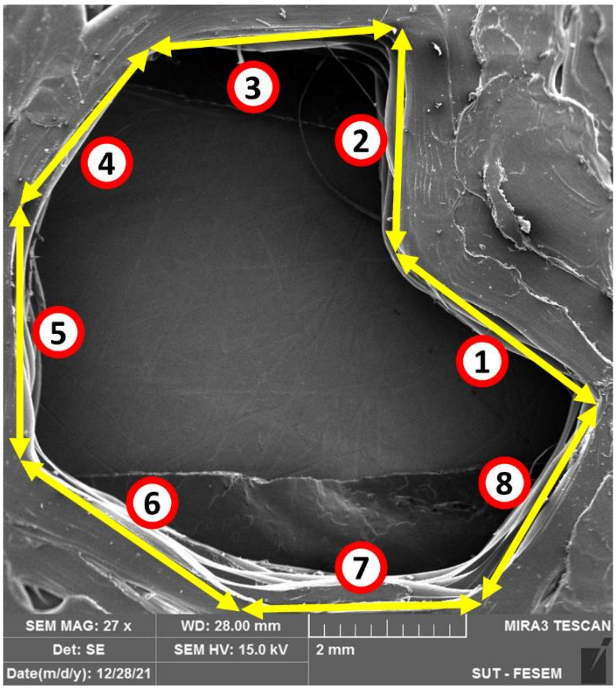

ID	Length (mm)	Angle (degree)
1	3.329	146.4
2	3.087	85.7
3	3.526	3.4
4	3.067	38.8
5	3.463	92.6
6	3.556	145.9
7	3.300	0.2
8	3.402	60.0

Fig 17: The dimensional accuracy of the irregular octagon, measured from FESEM micrographs of the 3D-printed PLA with 60% of infill

Fig 16 shows the measured values in an irregular octagon as a sample. The ImageJ software was used to measure the length and width of the lines, as well as the angle between them. The process repeated three times to ensure accuracy. The results were within a reasonable tolerance based on the difference between the demanded and measured values as well as the standard deviations according to Table 12. The comparison between the average values measured from the microscopic images and corresponding CAD models is done in this table. The purpose of this analysis was to assess how accurately the 3D printer was able to reproduce the intended geometry of the parts, and to identify any potential sources of error or variability in the printing process. By comparing the actual dimensions of the printed parts with the intended dimensions, it is possible to gain insight into the overall quality and reliability of the printing process, and to identify opportunities for improvement. A scatterplot was used to investigate the relationship between demanded and measured data (Fig 16). The rectangular sample with 40% of infill and the regular Octagon with 60% of infill was the worst parameters.

Table 12: The dimensional variations of samples printed as per the experimental design

Parameter / Fill pattern	Rectangular			Triangular	Honeycomb	Regular octagon	Irregular octagon
Demanded Infill (%)	40	60	80	60	60	60	60
Measured Infill (%)	74.61	77.44	90.27	69.7	75.55	70.24	71.66
Standard Deviation	86.53	29.07	12.84	16.17	25.92	17.07	19.43
Demanded Line Width (%)	2.75	2.75	2.75	4.50	1.25	1.50	2.50
Measured Line Width (%)	2.78	2.57	2.41	4.36	1.49	1.54	2.39
Standard Deviation	0.12	0.13	0.09	0.17	0.10	0.10	0.15
Demanded Line Length (mm)	0.50	1.25	3.00	1.00	1.60	1.25	3.3
Measured Line Length (mm)	0.83	1.18	3.41	1.02	1.57	1.46	3.39
Standard Deviation	0.10	0.14	0.13	0.05	0.11	0.22	0.09

Based on the scatterplot presented in Fig 18, it is evident that there exists a strong positive and linear relationship between the demanded and measured line widths (Fig 18(a)) as well as the demanded and measured line lengths (Fig 18(b)). This indicates that as the demanded line width or line length increases, there is a corresponding increase in the measured values, highlighting the consistent and proportional behavior between the two variables in the printing process. Dimension reduction compared to the designed value occurs as a result of excessive deposition of the material during the extrusion process (due to not considering the dwell time at the endpoints of raster paths) and transverse flow (due to the relationship between temperature and viscosity). This is while the increase in dimensions can be due to the shrinkage of the printed polymer [71]. When there is an inaccuracy in the nozzle position, an imperfect configuration will occur. However, this issue could be solved by the calibration of the 3D printing machine [68]. It seems that such inaccuracy increases with the decrease in filling percentage and therefore the thickness of the walls. The sum of these items results in differences in filling densities and should be considered in the design phase.

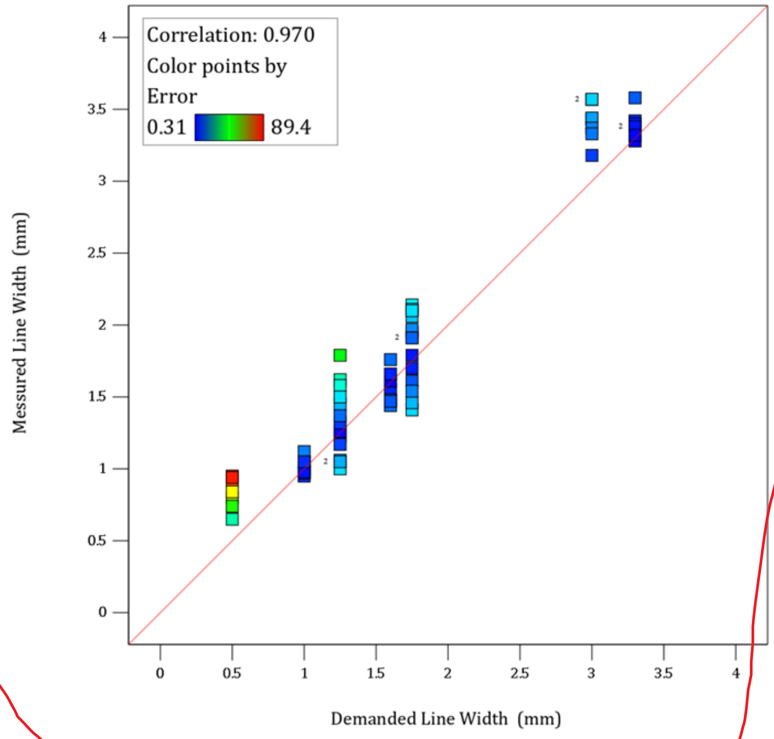

(a)

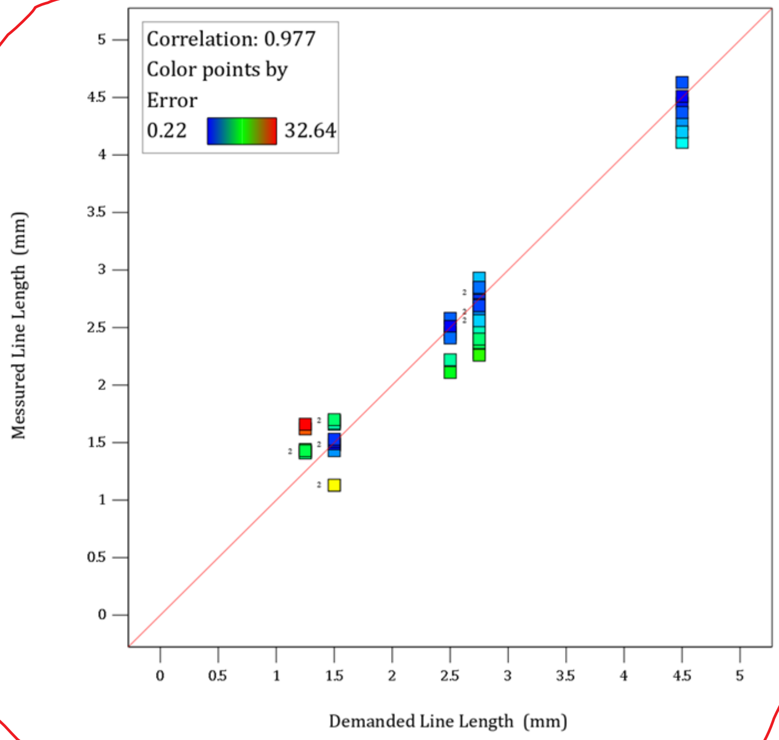

(b)

Fig 18: The scatter plot of demanded values versus the measured data for (a) the line length and (b) the line width

Designing for high-performance in additive manufacturing, despite its limitations, requires exploring various unit cells and meta-structures to identify patterns that interact effectively

with material behavior. In this research, the emphasis was primarily on numerical simulation and optimization of 3D-printed materials. The results were utilized to determine the parameters for experimental tests, the data of which has already been published [59-60]. For further investigations, the following subjects are suggested,

- Path planning and defining the route that the print head takes
- Providing topology optimization algorithms to achieve desired properties for specific applications
- Using exactly the material properties of the meta-structure and anisotropy (considering the 3D printing factors) for the lifetime prediction
- Examine the fatigue lifetime experimentally for the topology-optimized samples.

4) Conclusions

This research used multi-objective optimization to determine optimal parameters for PLA cylinders, considering fatigue lifetime, mass, and infill patterns. Structural topology optimization was conducted using a two-objective algorithm. Additionally, FESEM analysis ensured dimensional accuracy, and the influence of inherent defects in the FDM process was considered. The results are listed below:

- The stress analysis results showed that although the triangular pattern had the lowest mass in all cases, irregular octagon, rectangular and regular octagon had the lowest stresses in 40%, 60%, and 80% of the infill, respectively.
- The study introduced three sets of objective functions for design purposes, considering mass changes, fatigue lifetime changes, and the distance from the optimal point. The optimal solution was found to be the horizontally printed rectangular filling pattern with a 60% infill density. This pattern achieved a 39% reduction in weight while experiencing only a 22% decrease in fatigue lifetime. The mass reduction was determined through software analysis of designed CAD models, while the infill density was assessed using the ImageJ software.
- Topology optimization in Dassault Systems software proposed a metamaterial structure to minimize mass and strain energy under stress constraints. Achieved results: mass of 1.1767g, strain energy of 0.056 J (after 50 cycles), maximum von-Mises stress of 97.08 MPa. Microscopic inspections studied manufacturing defects and their impact on topology optimization.
- Shape optimization was performed to reduce stress concentration by displacing boundary nodes. Maximum von-Mises stress decreased to 44.30 MPa after 15 design cycles, leading to increased fatigue lifetime by 527 and 79 cycles compared to topology optimization in vertical and horizontal samples, respectively.
- Finally, a design realization step was performed, and a new geometry was introduced that has 96.70% lower stress and 201.69% higher mass compared with topology-optimized geometry. This is while this geometry is lighter than 60% infill samples with different patterns.
- Based on FESEM images, the rectangular pattern has the best regularity, while the regular octagon pattern was the most irregular.
- The line spacing is significant, especially in the honeycomb pattern, and should be controlled by printing temperature.
- The scatterplot shows a strong positive and linear relationship between the demanded and measured line length and line width.

- Inaccuracy in the position of the nozzle increases with the decrease in filling percentage and, therefore, the thickness of the walls. However, the rectangular sample with 40% infill and Regular Octagon with 60% infill was the worst parameters.

Declaration of competing interest

The authors illustrate that they have no competing financial interests or personal relationships that could have appeared to influence this work.

Data Availability

The datasets used and/or analyzed during the current study available from the corresponding author on reasonable request.

References

[1] "Additive manufacturing – General principles – Terminology, Standard," *Int. Organ. Stand.*, no. 52900:2015, 2015, doi: 10.1520/ISOASTM52900-15.

[2] M. Kun, C. Chan, S. Ramakrishna, A. Kulkarni, and K. Vadodaria, "Textile-based scaffolds for tissue engineering," in *Advanced Textiles for Wound Care*, Second Edi., Elsevier Ltd, 2019, pp. 329–362. doi: 10.1016/b978-0-08-102192-7.00012-6.

[3] F. N. Habib, P. Iovenitti, S. H. Masood, and M. Nikzad, "Fabrication of polymeric lattice structures for optimum energy absorption using multi jet fusion technology," *Mater. Des.*, vol. 155, pp. 86–98, 2018, doi: 10.1016/j.matdes.2018.05.059.

[4] S. M. Esfarjani, A. Dadashi, and M. Azadi, "Topology optimization of additive-manufactured metamaterial structures: A review focused on multi-material types," *Forces Mech.*, vol. 7, p. 100100, 2022, doi: 10.1016/J.FINMEC.2022.100100.

[5] S. Kumar, I. Singh, S. S. R. Koloor, D. Kumar, and M. Y. Yahya, "On Laminated Object Manufactured FDM-Printed ABS/TPU Multimaterial Specimens: An Insight into Mechanical and Morphological Characteristics," *Polymers (Basel).*, vol. 14, no. 19, p. 4066, 2022, doi: 10.3390/POLYM14194066.

[6] S. Kumar, I. Singh, D. Kumar, M. Y. Yahya, and S. S. Rahimian Koloor, "Mechanical and Morphological Characterizations of Laminated Object Manufactured 3D Printed Biodegradable Poly(lactic)acid with Various Physical Configurations," *J. Mar. Sci. Eng.*, vol. 10, no. 12, p. 1954, 2022, doi: 10.3390/JMSE10121954.

[7] J. Sun and N. M. Litchinitser, "Metamaterials," *Fundam. Appl. Nanophotonics*, pp. 253–307, 2016, doi: 10.1016/B978-1-78242-464-2.00009-9.

[8] H. Cui *et al.*, "Three-dimensional printing of piezoelectric materials with designed anisotropy and directional response," *Nat. Mater.*, vol. 18, pp. 234–241, 2019, doi: 10.1038/s41563-018-0268-1.

[9] X. Zheng *et al.*, "Ultralight, ultrastiff mechanical metamaterials," *Science (80-.).*, vol. 344, no. 6190, pp. 1373–1377, 2014, doi: 10.1126/science.1252291.

[10] S. Kumar, R. Singh, A. Batish, and T. P. Singh, "Additive manufacturing of smart materials exhibiting 4-D properties: A state of art review," vol. 35, no. 9, pp. 1358–1381, 2019, doi: 10.1177/0892705719895052.

[11] A. A. Zadpoor, "Meta-biomaterials," *Biomater. Sci.*, vol. 8, no. 1, pp. 18–38, 2019, doi: 10.1039/C9BM01247H.

[12] J. Fan *et al.*, "A review of additive manufacturing of metamaterials and developing trends," *Mater. Today*, vol. 50, pp. 303–328, 2021, doi:

10.1016/J.MATTOD.2021.04.019.
[13] W. Wu, P. Liu, and Z. Kang, "A novel mechanical metamaterial with simultaneous stretching- and compression-expanding property," *Mater.*, vol. 208, p. 109930, 2021, doi: 10.1016/j.matdes.2021.109930.
[14] G. Agrawal, A. Gupta, R. Chowdhury, and A. Chakrabarti, "Robust topology optimization of negative Poisson's ratio metamaterials under material uncertainty," *Finite Elem. Anal. Des.*, vol. 198, p. 103649, 2022, doi: 10.1016/J.FINEL.2021.103649.
[15] D. Li, W. Liao, N. Dai, G. Dong, Y. Tang, and Y. M. Xie, "Optimal design and modeling of gyroid-based functionally graded cellular structures for additive manufacturing," *CAD Comput. Aided Des.*, vol. 104, pp. 87–99, 2018, doi: 10.1016/j.cad.2018.06.003.
[16] Y. Tang, A. Kurtz, and Y. F. Zhao, "Bidirectional Evolutionary Structural Optimization (BESO) based design method for lattice structure to be fabricated by additive manufacturing," *CAD Comput. Aided*, vol. 69, pp. 91–101, 2015, doi: 10.1016/j.cad.2015.06.001.
[17] B. Fox and A. Subic, "An industry 4.0 approach to the 3D printing of composite materials," *Engineering*, vol. 5, no. 4, pp. 621–623, 2019, doi: 10.1016/j.eng.2019.06.003.
[18] A. P. Garland, B. C. White, B. H. Jared, M. Heiden, E. Donahue, and B. L. Boyce, "Deep convolutional neural networks as a rapid screening tool for complex additively manufactured structures," *Addit. Manuf.*, vol. 35, p. 101217, 2020, doi: 10.1016/j.addma.2020.101217.
[19] X. Qi, G. Chen, Y. Li, X. Cheng, and C. Li, "Applying neural-network-based machine learning to additive manufacturing: current applications, challenges, and future perspectives," *Engineering*, vol. 5, no. 4, pp. 721–729, 2019, doi: 10.1016/j.eng.2019.04.012.
[20] G. X. Gu, C. T. Chen, D. J. Richmond, and M. J. Buehler, "Bioinspired hierarchical composite design using machine learning: Simulation, additive manufacturing, and experiment," *Mater. Horizons*, vol. 5, pp. 939–945, 2018, doi: 10.1039/c8mh00653a.
[21] G. X. Gu, C. T. Chen, and M. J. Buehler, "De novo composite design based on machine learning algorithm," *Extrem. Mech. Lett.*, vol. 18, pp. 19–28, 2018, doi: 10.1016/j.eml.2017.10.001.
[22] C. Qian, R. K. Tan, and W. Ye, "Design of architectured composite materials with an efficient, adaptive artificial neural network-based generative design method," *Acta Mater.*, vol. 225, p. 117548, Feb. 2022, doi: 10.1016/J.ACTAMAT.2021.117548.
[23] Peter W. Christensen and A. Klarbring, "An Introduction to Structural Optimization," *Springer Sci. Bus. Media*, 2019.
[24] J. Zhu, H. Zhou, C. Wang, L. Zhou, S. Yuan, and W. Zhang, "A review of topology optimization for additive manufacturing: Status and challenges," *Chinese J. Aeronaut.*, vol. 34, no. 1, pp. 91–110, 2021, doi: 10.1016/j.cja.2020.09.020.
[25] J. P. Meng, B. Fang, and H. B. Tan, "Crystal structure of methyl 2-(4-(3-(2,4-difluorophenyl)pyrazolo[1,5-a]pyrimidin-6-yl)phenyl)acetate, C21H15F2N3O2," vol. 234, no. 4, pp. 805–807, 2019.
[26] J. Liu *et al.*, "Current and future trends in topology optimization for additive manufacturing," *Struct. Multidiscip. Optim.*, vol. 57, pp. 2457–2483, 2018, doi: 10.1007/s00158-018-1994-3.
[27] J. Kiendl and C. Gao, "Controlling toughness and strength of FDM 3D-printed PLA components through the raster layup," *Compos. Part B Eng.*, vol. 180, p. 107562, 2020,

doi: 10.1016/j.compositesb.2019.107562.
[28] S. Li, S. Yuan, J. Zhu, C. Wang, J. Li, and W. Zhang, "Additive manufacturing-driven design optimization: Building direction and structural topology," *Addit. Manuf.*, vol. 36, p. 101406, 2020, doi: 10.1016/j.addma.2020.101406.
[29] S. Li, "Why We need a global history of political thought," *Concepts Hist. John Dunn. Lect. China. Palgrave Macmillan, Singapore.*, 2021.
[30] J. Wu, A. Clausen, and O. Sigmund, "Minimum compliance topology optimization of shell–infill composites for additive manufacturing," *Comput. Methods Appl. Mech. Eng.*, vol. 326, pp. 358–375, 2017, doi: 10.1016/j.cma.2017.08.018.
[31] N. S. F. Jap, G. M. Pearce, A. K. Hellier, N. Russell, W. C. Parr, and W. R. Walsh, "The effect of raster orientation on the static and fatigue properties of filament deposited ABS polymer," *Int. J. Fatigue*, vol. 124, pp. 328–337, 2019, doi: 10.1016/j.ijfatigue.2019.02.042.
[32] T. Pepelnjak, J. Stojšić, L. Sevšek, D. Movrin, and M. Milutinović, "Influence of Process Parameters on the Characteristics of Additively Manufactured Parts Made from Advanced Biopolymers," *Polymers (Basel).*, vol. 15, no. 3, p. 716, 2023, doi: 10.3390/POLYM15030716.
[33] C. Abeykoon, P. Sri-Amphorn, and A. Fernando, "Optimization of fused deposition modeling parameters for improved PLA and ABS 3D printed structures," *Int. J. Light. Mater. Manuf.*, vol. 3, no. 3, pp. 284–297, 2020, doi: 10.1016/j.ijlmm.2020.03.003.
[34] T. N. A. T. Rahim, A. M. Abdullah, and H. Md Akil, "Recent developments in fused deposition modeling-based 3D printing of polymers and their composites," *Polym. Rev.*, vol. 59, no. 4, pp. 589–624, 2019, doi: 10.1080/15583724.2019.1597883.
[35] Y. G. Zhou, J. R. Zou, H. H. Wu, and B. P. Xu, "Balance between bonding and deposition during fused deposition modeling of polycarbonate and acrylonitrile-butadiene-styrene composites," *Polym. Compos.*, vol. 41, no. 1, pp. 60–72, 2020, doi: 10.1002/pc.25345.
[36] I. Bin Ishak, D. Fleming, and P. Larochelle, "Multiplane fused deposition modeling: a study of tensile strength," *Mech. Based Des. Struct. Mach.*, vol. 47, no. 5, pp. 583–598, 2019, doi: 10.1080/15397734.2019.1596127.
[37] A. K. Sood, R. K. Ohdar, and S. S. Mahapatra, "Parametric appraisal of mechanical property of fused deposition modelling processed parts," *Mater. Des.*, vol. 31, no. 1, pp. 287–295, 2010, doi: 10.1016/j.matdes.2009.06.016.
[38] M. Fernandez-Vicente, W. Calle, S. Ferrandiz, and A. Conejero, "Effect of Infill parameters on tensile mechanical behavior in desktop 3D printing," *3D Print. Addit. Manuf.*, vol. 3, no. 3, pp. 183–192, 2016, doi: 10.1089/3dp.2015.0036.
[39] R. Baptista and M. Guedes, "Porosity and pore design influence on fatigue behavior of 3D printed scaffolds for trabecular bone replacement," *J. Mech. Behav. Biomed. Mater.*, vol. 117, p. 104378, 2021, doi: 10.1016/j.jmbbm.2021.104378.
[40] T. Pepelnjak, A. Karimi, A. Maček, and N. Mole, "Altering the Elastic Properties of 3D Printed Poly-Lactic Acid (PLA) Parts by Compressive Cyclic Loading," *Mater*, vol. 13, no. 19, p. 4456, 2020, doi: 10.3390/MA13194456.
[41] N. A. Fountas, J. D. Kechagias, D. E. Manolakos, and N. M. Vaxevanidis, "Single and multi-objective optimization of FDM-based additive manufacturing using metaheuristic algorithms," *Procedia Manuf.*, vol. 51, pp. 740–747, 2020, doi: 10.1016/J.PROMFG.2020.10.104.
[42] N. Yodo and A. Dey, "Multi-Objective Optimization for FDM Process Parameters with

Evolutionary Algorithms," pp. 419–444, 2021, doi: 10.1007/978-3-030-68024-4_22.
[43] J. John, D. Devjani, S. Ali, S. Abdallah, and S. Pervaiz, "Optimization of 3D printed polylactic acid structures with different infill patterns using Taguchi-grey relational analysis," *Adv. Ind. Eng. Polym. Res.*, 2022, doi: 10.1016/J.AIEPR.2022.06.002.
[44] C. Abeykoon, P. Sri-Amphorn, and A. Fernando, "Optimization of fused deposition modeling parameters for improved PLA and ABS 3D printed structures," *Int. J. Light. Mater. Manuf.*, vol. 3, no. 3, pp. 284–297, 2020, doi: 10.1016/J.IJLMM.2020.03.003.
[45] B. N. Turner and S. A. Gold, "A review of melt extrusion additive manufacturing processes: II. Materials, dimensional accuracy, and surface roughness," *Rapid Prototyp. J.*, vol. 21, no. 3, pp. 250–261, 2015, doi: 10.1108/RPJ-02-2013-0017.
[46] S. H. Ahn, C. Baek, S. Lee, and I. S. Ahn, "Anisotropic Tensile Failure Model of Rapid Prototyping Parts - Fused Deposition Modeling (FDM)," *Int. J. Mod. Phys. B*, vol. 17, no. 08n09, pp. 1510–1516, 2003, doi: 10.1142/S0217979203019241.
[47] S. Farah, D. G. Anderson, and R. Langer, "Physical and mechanical properties of PLA, and their functions in widespread applications — A comprehensive review," *Adv. Drug Deliv. Rev.*, vol. 107, pp. 367–392, 2016, doi: 10.1016/j.addr.2016.06.012.
[48] O. H. Ezeh and L. Susmel, "On the fatigue strength of 3D-printed polylactide (PLA)," *Procedia Struct. Integr.*, vol. 9, pp. 29–36, 2018, doi: 10.1016/j.prostr.2018.06.007.
[49] S. Dezianian and M. Azadi, "Multi-Material Metamaterial Topology Optimization to Minimize the Compliance and the Constraint of Weight: Application of Non-Pneumatic Tire Additive-Manufactured with PLA/TPU Polymers," *Polymers (Basel).*, vol. 15, no. 8, p. 1927, 2023, doi: 10.3390/POLYM15081927.
[50] A. Karimi, N. Mole, and T. Pepelnjak, "Numerical Investigation of the Cycling Loading Behavior of 3D-Printed Poly-Lactic Acid (PLA) Cylindrical Lightweight Samples during Compression Testing," *Appl. Sci.*, vol. 12, no. 16, p. 8018, 2022, doi: 10.3390/APP12168018.
[51] B. Kosic, M. Stojicevic, Z. Jeli, B. Pokonstantinovic, A. Duta, and A. Dragicevic, "3D analysis of diferent metamaterial geometry and simulation of metamaterial usage," *FME Trans.*, vol. 47, no. 2, pp. 349–354, 2019, doi: 10.5937/fmet1902349K.
[52] J. Antonio Travieso-Rodriguez, M. D. Zandi, R. Jerez-Mesa, and J. Lluma-Fuentes, "Fatigue behavior of PLA-wood composite manufactured by fused filament fabrication," *J. Mater. Res. Technol.*, vol. 9, no. 4, pp. 8507–8516, 2020, doi: 10.1016/j.jmrt.2020.06.003.
[53] S. N. I. 1143, "Metallic materials - Rotating bar bending fatigue testing," *ISO Int. Stand.*, 2010.
[54] DASSAULT SYSTEMES and Simulia, "Abaqus Analysis User's Manual," 2016.
[55] M. S. Aghareb Parast, A. Bagheri, A. Kami, M. Azadi, and V. Asghari, "Bending fatigue behavior of fused filament fabrication 3D-printed ABS and PLA joints with rotary friction welding," *Prog. Addit. Manuf.*, pp. 1–17, 2022, doi: 10.1007/S40964-022-00307-5.
[56] M. Azadi, A. Dadashi, S. Dezianian, M. Kianifar, S. Torkaman, and M. Chiyani, "High-cycle bending fatigue properties of additive-manufactured ABS and PLA polymers fabricated by fused deposition modeling 3D-printing," *Forces Mech.*, vol. 3, p. 100016, Sep. 2021, doi: 10.1016/j.finmec.2021.100016.
[57] N. Wang, W. jie Zhao, N. Wu, and D. Wu, "Multi-objective optimization: A method for selecting the optimal solution from Pareto non-inferior solutions," *Expert Syst. Appl.*, vol. 74, pp. 96–104, 2017, doi: 10.1016/j.eswa.2017.01.004.

[58] A. R. Aziz, J. Zhou, D. Thorne, and W. J. Cantwell, "Geometrical Scaling Effects in the Mechanical Properties of 3D-Printed Body-Centered Cubic (BCC) Lattice Structures," *Polym*, vol. 13, no. 22, p. 3967, 2021, doi: 10.3390/POLYM13223967.
[59] M. Azadi and A. Dadashi, "Experimental fatigue dataset for additive-manufactured 3D-printed Polylactic acid biomaterials under fully-reversed rotating-bending bending loadings," *Data Br.*, vol. 41, p. 107846, 2022, doi: 10.1016/J.DIB.2022.107846.
[60] A. Dadashi and M. Azadi, "Experimental bending fatigue data of additive-manufactured PLA biomaterial fabricated by different 3D printing parameters," *Prog. Addit. Manuf.*, 2022, doi: 10.1007/S40964-022-00327-1.
[61] H. Gonabadi, A. Yadav, and S. J. Bull, "The effect of processing parameters on the mechanical characteristics of PLA produced by a 3D FFF printer," *Int. J. Adv. Manuf. Technol.*, vol. 111, pp. 695–709, 2020, doi: 10.1007/s00170-020-06138-4.
[62] B. Aloyaydi, S. Sivasankaran, and A. Mustafa, "Investigation of infill-patterns on mechanical response of 3D printed poly-lactic-acid," *Polym. Test.*, vol. 87, p. 106557, 2020, doi: 10.1016/j.polymertesting.2020.106557.
[63] A. D. Pertuz, S. Díaz-Cardona, and O. A. González-Estrada, "Static and fatigue behaviour of continuous fibre reinforced thermoplastic composites manufactured by fused deposition modelling technique," *Int. J. Fatigue*, vol. 130, p. 105275, 2020, doi: 10.1016/j.ijfatigue.2019.105275.
[64] J. Naranjo-Lozada, H. Ahuett-Garza, P. Orta-Castañón, W. M. H. Verbeeten, and D. Sáiz-González, "Tensile properties and failure behavior of chopped and continuous carbon fiber composites produced by additive manufacturing," *Addit. Manuf.*, vol. 26, pp. 227–241, Mar. 2019, doi: 10.1016/j.addma.2018.12.020.
[65] V. Cabreira and R. M. C. Santana, "Effect of infill pattern in Fused filament fabrication (FFF) 3D printing on materials performance," *Matéria*, vol. 25, no. 3, pp. 1–9, 2020.
[66] K. Vigneshwaran and N. Venkateshwaran, "Statistical analysis of mechanical properties of wood-PLA composites prepared via additive manufacturing," *Int. J. Polym. Anal. Charact.*, vol. 24, no. 7, pp. 584–596, 2019, doi: 10.1080/1023666X.2019.1630940.
[67] H. Mei, Z. Ali, I. Ali, and L. Cheng, "Tailoring strength and modulus by 3D printing different continuous fibers and filled structures into composites," *Adv. Compos. Hybrid Mater.*, vol. 2, pp. 312–319, 2019, doi: 10.1007/s42114-019-00087-7.
[68] M. Frascio, M. Avalle, and M. Monti, "Fatigue strength of plastics components made in additive manufacturing: First experimental results," *Procedia Struct. Integr.*, vol. 12, pp. 32–43, 2018, doi: 10.1016/j.prostr.2018.11.109.
[69] A. A. Ansari and M. Kamil, "Effect of print speed and extrusion temperature on properties of 3D printed PLA using fused deposition modeling process," *Mater. Today Proc.*, vol. 45, pp. 5462–5468, 2021, doi: 10.1016/j.matpr.2021.02.137.
[70] B. N. Turner, R. Strong, and S. A. Gold, "A review of melt extrusion additive manufacturing processes: I. Process design and modeling," *Rapid Prototyp. J.*, vol. 20, no. 3, pp. 192–204, 2014, doi: 10.1108/RPJ-01-2013-0012/FULL/XML.
[71] S. Singamneni, M. P. Behera, D. Truong, M. J. Le Guen, E. Macrae, and K. Pickering, "Direct extrusion 3D printing for a softer PLA-based bio-polymer composite in pellet form," *J. Mater. Res. Technol.*, vol. 15, pp. 936–949, Nov. 2021, doi: 10.1016/J.JMRT.2021.08.044.

Supporting information

Fig. S1: The GIF file for the developments during the topology optimization

Dear Editor-in-Chief,
PLOS ONE

Authors thank you for giving me the opportunity to revise my paper titled “Multi-objective numerical optimization of 3D-printed polylactic acid bio-metamaterial based on topology, filling pattern, and infill density via fatigue lifetime and mass”. I appreciate the valuable feedback and comments provided by the reviewers, which have helped me to improve the quality and clarity of my manuscript. In this revision letter, I will address each of the reviewers' comments and explain how I have addressed them in the revised version of the paper. I hope that the changes I have made will sufficiently address the concerns raised and that the revised manuscript will be suitable for publication in PLOS ONE.

Regards,
M. Azadi, PhD.
Faculty of Mechanical Engineering, Semnan University, Semnan, Iran

Journal requirements:

When submitting your revision, we need you to address these additional requirements.

Answer: In order to address these additional requirements, we have carefully reviewed the manuscript and made the necessary changes to ensure that it meets all of the submission guidelines and requirements of PLOS ONE. We appreciate the editor's feedback and will work diligently to address all of the necessary revisions.

1. Please ensure that your manuscript meets PLOS ONE's style requirements, including those for file naming. The PLOS ONE style templates can be found at
https://journals.plos.org/plosone/s/file?id=wjVg/PLOSOne_formatting_sample_main_body.pdf
and
https://journals.plos.org/plosone/s/file?id=ba62/PLOSOne_formatting_sample_title_authors_affiliations.pdf

Answer: We have reviewed and updated our manuscript accordingly, including file naming and formatting, using the PLOS ONE style templates provided. Please let us know if there are any further issues with the formatting of our manuscript.

2. Please note that PLOS ONE has specific guidelines on code sharing for submissions in which author-generated code underpins the findings in the manuscript. In these cases, all author-generated code must be made available without restrictions upon publication of the work. Please review our guidelines at https://journals.plos.org/plosone/s/materials-and-software-sharing#loc-sharing-code and ensure that your code is shared in a way that follows best practice and facilitates reproducibility and reuse.

Answer: We confirm that the findings in our manuscript were not underpinned by any author-generated code, and hence, we have no code to share. We have reviewed the PLOS ONE guidelines on code sharing and ensured that we have followed best practices for the materials and software used in our study.

3. We note that the grant information you provided in the ‘Funding Information’ and ‘Financial Disclosure’ sections do not match.
When you resubmit, please ensure that you provide the correct grant numbers for the awards you received for your study in the ‘Funding Information’ section.

Answer: Thank you for bringing this to our attention. We have reviewed our records and have corrected the grant number in the 'Funding Information' section to accurately reflect the grant we received from our funding agency. We appreciate your attention to detail and will ensure that all information is accurate in our revised submission.

4. Thank you for stating the following in the Acknowledgments Section of your manuscript:
"The authors would like to acknowledge the financial support of the Iran Small Industries and Industrial Parks Organization (ISIPO) for this project under grant number of 23607."
We note that you have provided funding information that is currently declared in your Funding Statement. However, funding information should not appear in the Acknowledgments section or other areas of your manuscript. We will only publish funding information present in the Funding Statement section of the online submission form.
Please remove any funding-related text from the manuscript and let us know how you would like to update your Funding Statement. Currently, your Funding Statement reads as follows:
"The authors would like to acknowledge the financial support of the Iran Small Industries and Industrial Parks Organization (ISIPO) for this project under grant number of 23607."
Please include your amended statements within your cover letter; we will change the online submission form on your behalf.
Answer: Thank you for bringing this to our attention. We have removed the funding-related text from the Acknowledgments section and have updated our Funding Statement accordingly in the cover letter as requested. Regarding the Funding Statement section of the online submission form, I would like to amend it as follows:

> *"The research reported in this manuscript was financially supported by the Iran Small Industries and Industrial Parks Organization (ISIPO) under a grant number of 23607."*

5. Please include captions for your Supporting Information files at the end of your manuscript, and update any in-text citations to match accordingly. Please see our Supporting Information guidelines for more information: http://journals.plos.org/plosone/s/supporting-information.
Answer: Thank you for your comment. We included captions for our Supporting Information files at the end of the manuscript and update any in-text citations to match accordingly, following the guidelines provided by PLOS ONE.

> *The optimized geometry in Fig 9 is reached after 50 design cycles. An animation is also included in supporting information (Fig S1) that shows the topology optimization process developments during each design cycle, providing a visual representation of the design process. Such visual support presents a better understanding of the design evolution and the optimization steps taken to arrive at the final design.*
> *"Fig. S1: The GIF file for the developments during the topology optimization"*

6. We note that you have indicated that data from this study are available upon request. PLOS only allows data to be available upon request if there are legal or ethical restrictions on sharing data publicly. For more information on unacceptable data access restrictions, please see http://journals.plos.org/plosone/s/data-availability#loc-unacceptable-data-access-restrictions.
In your revised cover letter, please address the following prompts:
a) If there are ethical or legal restrictions on sharing a de-identified data set, please explain them in detail (e.g., data contain potentially sensitive information, data are owned by a third-party organization, etc.) and who has imposed them (e.g., an ethics committee). Please also provide contact information for a data access committee, ethics committee, or other institutional body to which data requests may be sent.

b) If there are no restrictions, please upload the minimal anonymized data set necessary to replicate your study findings as either Supporting Information files or to a stable, public repository and provide us with the relevant URLs, DOIs, or accession numbers. For a list of acceptable repositories, please see http://journals.plos.org/plosone/s/data-availability#loc-recommended-repositories.
We will update your Data Availability statement on your behalf to reflect the information you provide.
Answer: No primary data collection or analysis conducted. Information sourced from publicly available data, properly cited and referenced. No additional data set for sharing. Study findings can be replicated based on provided details.

Reviewer #1:

Dear Authors,

Your paper is interested and well structured, however some questions arose during the review process:

Answer: Authors firstly thank the respected reviewer to his/her beneficial comments. All comments, one by one, have carefully addressed in the revised article and also, answered in following paragraphs for next comments. Then, these words were also changed and highlighted in the revised article.

1. Introduction: More extensive presentation of Pareto optimization needs to be done with emphasis on the research in the presented research area.

Answer: Based on the reviewer's comment, we have updated the introduction section to include a more extensive presentation of Pareto optimization with a focus on its relevance to the presented research area.

> *Fountas et al. [41] examined the performance of different swarm-based evolutionary algorithms in optimizing single and multi-objective problems related to additive manufacturing, specifically fused deposition modeling (FDM). The results showed that algorithms do not perform equally when applied to different optimization problems, and the quality of Pareto non-dominated solutions is an important indicator for multi-objective problems. Yodo and Dey [42] introduced multi-objective optimization methods based on evolutionary algorithms to optimize FDM process parameters, leading to Pareto-optimal solutions for decision-makers.*

2. Page 3 (P3): works are also done presenting the compressive loading of the PLA printed material - consider DOI: 10.3390/ma13194456

Answer: Thank you for bringing this article to our attention. We have added it to our literature review and have incorporated the findings into our introduction.

> *Moreover, the influence of infill line distance on the compressive elastic behavior of 3D printed circular samples was investigated by Pepelnjak et al. [40] They observed a significant difference in the first cycle response and an alteration of stiffness due to elastic and plastic deformation under cyclic*
>
> [40] T. Pepelnjak, A. Karimi, A. Maček, and N. Mole, "Altering the Elastic Properties of 3D Printed Poly-Lactic Acid (PLA) Parts by Compressive Cyclic Loading," *Mater*, vol. 13, no. 19, p. 4456, 2020, doi: 10.3390/MA13194456.

3. Recently a review of the influence of printing parameters on mechanical properties was published: consider their findings: https://doi.org/10.3390/polym15030716

Answer: The authors carefully considered respected reviewer's comment and incorporated the relevant publication into our revised manuscript, enhancing our analysis of the printing parameters' impact on mechanical properties. We appreciate your suggestion, as it has contributed to the overall improvement and significance of our study.

> *3D printing process parameters have a significant impact on the mechanical properties of printed parts, including printing temperature, speed, filling density, pattern, layer thickness, material properties, surface roughness, and layer thickness [31-33].*

[32] T. Pepelnjak, J. Stojšić, L. Sevšek, D. Movrin, and M. Milutinović, "Influence of Process Parameters on the Characteristics of Additively Manufactured Parts Made from Advanced Biopolymers," *Polymers (Basel).*, vol. 15, no. 3, p. 716, 2023, doi: 10.3390/POLYM15030716.

4, Material and material properties - you mention two references. However, are all those material properties from your table 1 experimentally determined?

Answer: We appreciate the respected reviewer's comment on the source of material properties in Table 1. The values were obtained from the material manufacturer's datasheets, except for the endurance limit which was experimentally tested by Dada. We have addressed this clarification in the revised manuscript.

> *Density, elastic module, yield strength, ultimate tensile strength, and Poisson ratio were obtained from technical datasheets provided by the material manufacturer. These properties are typically reported by the manufacturer based on standard testing methods [47]. Though, endurance limit, which refers to the maximum stress amplitude that a material can withstand for a given number of cycles without failure, cannot be obtained from technical datasheets and requires experimental testing. The endurance limit used in this study [48] was experimental obtained.*

5. In chapter 2-2 the test specimen also numerically verified is also dimensional presented. It has diameter of 9 mm with a shell of 0,4 mm on its surface. How you can then claim that the element size of 0.7 mm is optimal for your evaluations. This is highly doubtful and needs further explanation. Additionally, with this ratio of specimen diameter versus element size you have only 13 elements on the cross-section. Finally, the selection of the element size needs to be discussed more into the detail.

Answer: We acknowledge the reviewer's valid concern about element size and low element count on the cross-section. To address this, we adjusted the element size using software parameters and ensured an adequate representation of the specimen's geometry. Specifically, we set the minimum size control and curvature control parameters to address thin areas and maintain accuracy. The resulting mesh contained more than 13 elements, providing reliable results.

> *There were some areas with thickness less than 0.7 mm. In such cases, to prevent the distorted elements in these thin areas, the minimum size control parameter (set to 0.1 of the global size) and the curvature control parameter (set with a maximum deviation factor of 0.1) adjusted the element size. Increasing the maximum deviation factor allows for a greater deviation between the actual geometry and the approximated geometry, and can be useful in ensuring mesh quality in thin regions. The minimum size control and curvature control parameters used in this study were recommended by the ABAQUS software algorithm.*

6. Figure 2 - font in the upper row is too large.

Answer: In response to the respected reviewer’s comment, we have revised Figure 2 to adjust the font size in the upper row to ensure consistency with the font size in the lower row. The modified figure could be seen as follows,

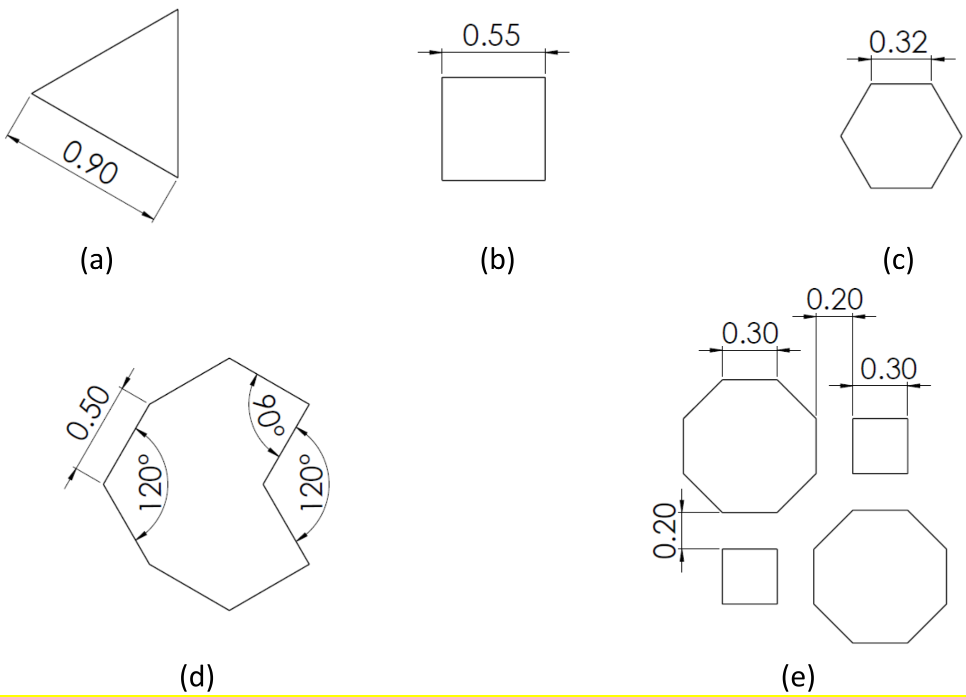

Fig 2: Different filling patterns: (a) triangular, (b) rectangular, (c) honeycomb, (d) irregular octagon, and (e) regular octagon

7. Figure 5 presents the used machine for specimen printing. The scale is missing on the figure. The data about the printer's accuracy is missing.

Answer: The device size is added to Figure 5 to indicate the printer's dimensions to address the respected reviewer’s comment. The modified figure could be seen as follows,

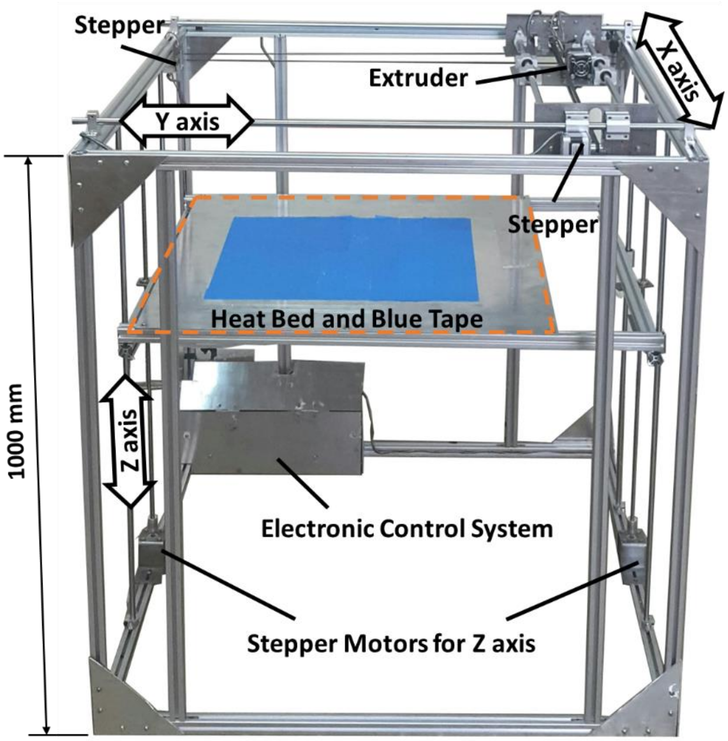

Fig 5: The FDM 3D printer used for additive manufacturing of the specimens

8. It is noted that the samples have been printed 5x larger as the ones used in the CAD model. Why. This needs to be explained into the detail. Furthermore, the comment about the influence of this scaling is missing.

Answer: The decision to print the samples at a larger scale was driven by limitations in accurately printing complex geometries at a smaller scale. While this allowed to achieve the desired accuracy, the reviewer's point about the influence of scaling is valid. The authors have included a discussion in the revised version to address the need for future investigation on the influence of scaling on geometrical accuracy and mechanical properties of lattice structures.

> *To ensure optimal results and utilize the capabilities of an industrial-duty 3D-printer machine, the samples were printed at a scale of 5 times larger than the original CAD models. One other reason is due to the machine limitations. This approach allowed to produce lattice structures with circular sections and a diameter of 45 millimeters, which would have been challenging to achieve with smaller scales. While this approach allowed for the successful printing of the specimens, it is important to investigate the potential effects of scaling on the accuracy of the geometrical features and the resulting mechanical properties in future investigations. Previous studies demonstrated clear size effects on the mechanical response of additively manufactured lattice structures [58].*

9. Table 7: first column is too narrow and cuts the text - correct this. Also in this table, you present the Von Mises stress distributions. Can you compare them also with the experimental data? From this comparison it is also not evident what is compressive and what tensile loaded. This need to be corrected and need also further explanation.

Answer: We appreciate the reviewer's feedback. The column width in Table 7 will be adjusted to prevent text truncation. Additionally, the contour stress in the direction of sample axis (σyy) is included in Table 8 to demonstrate the distribution of normal stress. While it is not feasible to experimentally determine stress distribution throughout the entire fatigue life, ongoing research is being conducted to measure the fatigue behavior of PLA material. This clarification has been added to the revised article in response to the reviewer's comment.

> *As shown in stress distribution contours, the bending load generates compressive stress on the bottom side of the clamped end and tensile stress on its top side. However, in fatigue testing, the load is cyclic and changes the loading direction periodically, which leads to alternating regions of compressive and tensile stresses. Therefore, the specimens undergo both compressive and tensile stresses during fatigue testing, which can affect their durability and mechanical performance. Although the stress distribution in PLA specimens under cyclic loading cannot be measured experimentally, the fatigue behavior of the material is still worth investigating in future researches. This information can be compared to numerical results.*

Table 7: The von-Mises stress distribution contours for different patterns

<table>
<tr><th>Pattern</th><th>40%</th><th>60%</th><th>80%</th></tr>
<tr><td>Honeycomb</td><td>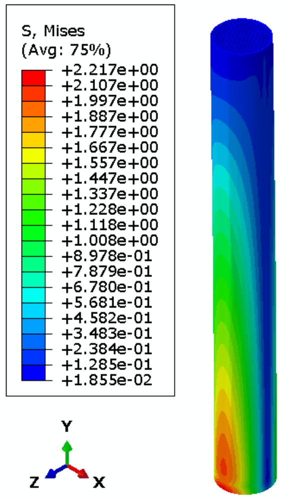
</td><td>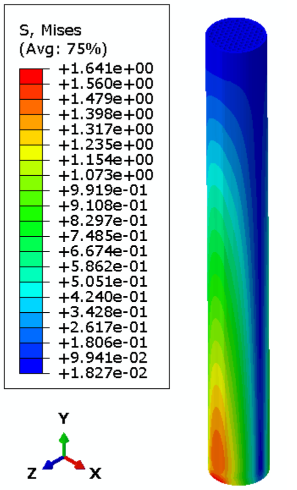
</td><td>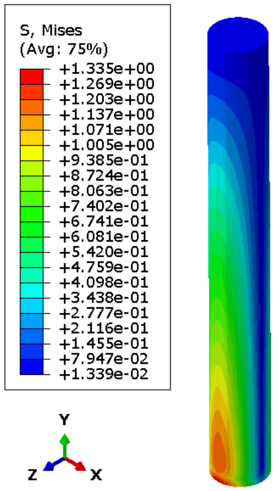
</td></tr>
</table>

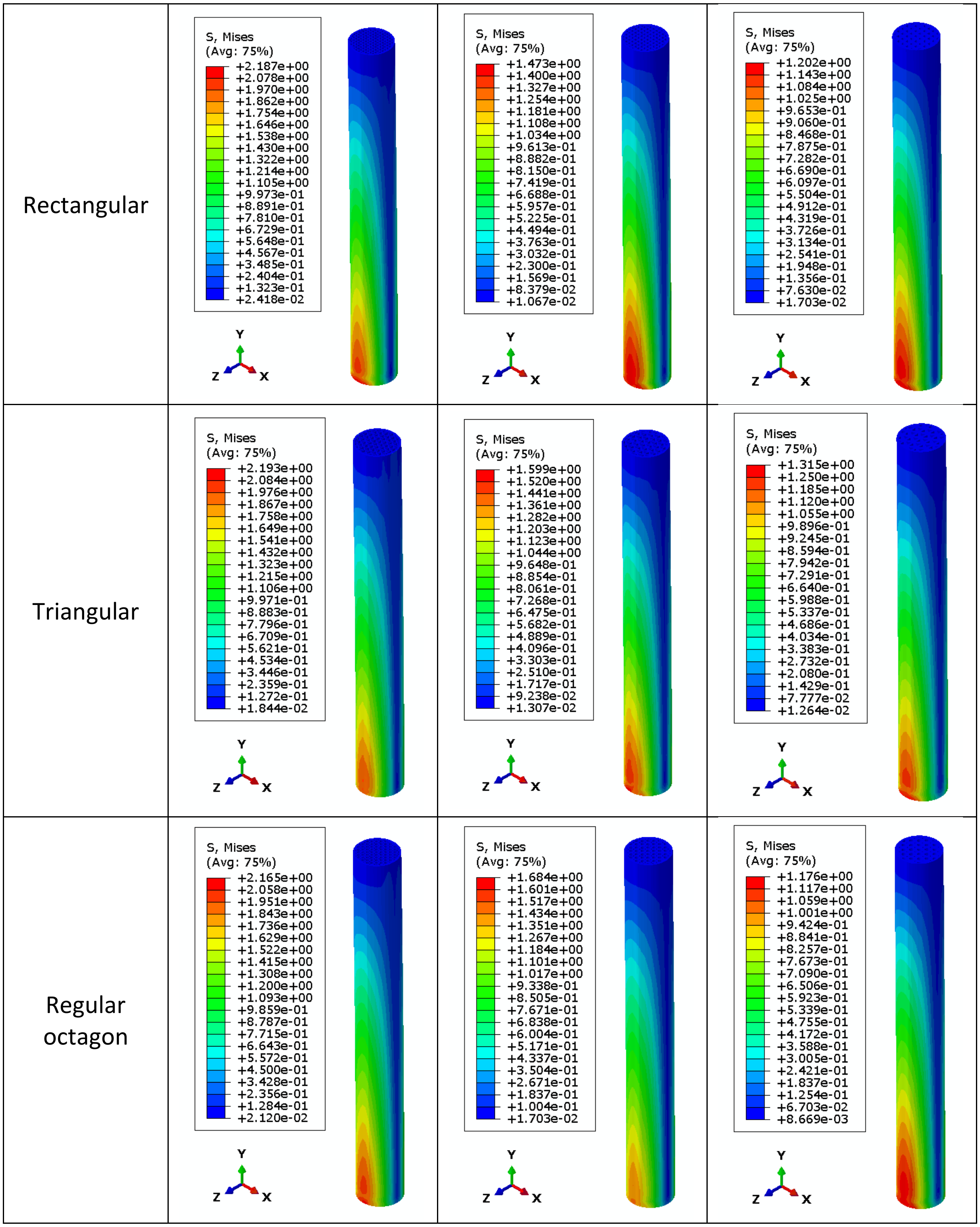
Rectangular
S, Mises
(Avg: 75%)
+2.187e+00
+2.078e+00
+1.970e+00
+1.862e+00
+1.754e+00
+1.646e+00
+1.538e+00
+1.430e+00
+1.322e+00
+1.214e+00
+1.105e+00
+9.973e-01
+8.891e-01
+7.810e-01
+6.729e-01
+5.648e-01
+4.567e-01
+3.485e-01
+2.404e-01
+1.323e-01
+2.418e-02
Y
Z
X
S, Mises
(Avg: 75%)
+1.473e+00
+1.400e+00
+1.327e+00
+1.254e+00
+1.181e+00
+1.108e+00
+1.034e+00
+9.613e-01
+8.882e-01
+8.150e-01
+7.419e-01
+6.688e-01
+5.957e-01
+5.225e-01
+4.494e-01
+3.763e-01
+3.032e-01
+2.300e-01
+1.569e-01
+8.379e-02
+1.067e-02
Y
Z
X
S, Mises
(Avg: 75%)
+1.202e+00
+1.143e+00
+1.084e+00
+1.025e+00
+9.653e-01
+9.060e-01
+8.468e-01
+7.875e-01
+7.282e-01
+6.690e-01
+6.097e-01
+5.504e-01
+4.912e-01
+4.319e-01
+3.726e-01
+3.134e-01
+2.541e-01
+1.948e-01
+1.356e-01
+7.630e-02
+1.703e-02
Y
Z
X
Triangular
S, Mises
(Avg: 75%)
+2.193e+00
+2.084e+00
+1.976e+00
+1.867e+00
+1.758e+00
+1.649e+00
+1.541e+00
+1.432e+00
+1.323e+00
+1.215e+00
+1.106e+00
+9.971e-01
+8.883e-01
+7.796e-01
+6.709e-01
+5.621e-01
+4.534e-01
+3.446e-01
+2.359e-01
+1.272e-01
+1.844e-02
Y
Z
X
S, Mises
(Avg: 75%)
+1.599e+00
+1.520e+00
+1.441e+00
+1.361e+00
+1.282e+00
+1.203e+00
+1.123e+00
+1.044e+00
+9.648e-01
+8.854e-01
+8.061e-01
+7.268e-01
+6.475e-01
+5.682e-01
+4.889e-01
+4.096e-01
+3.303e-01
+2.510e-01
+1.717e-01
+9.238e-02
+1.307e-02
Y
Z
X
S, Mises
(Avg: 75%)
+1.315e+00
+1.250e+00
+1.185e+00
+1.120e+00
+1.055e+00
+9.896e-01
+9.245e-01
+8.594e-01
+7.942e-01
+7.291e-01
+6.640e-01
+5.988e-01
+5.337e-01
+4.686e-01
+4.034e-01
+3.383e-01
+2.732e-01
+2.080e-01
+1.429e-01
+7.777e-02
+1.264e-02
Y
Z
X
Regular octagon
S, Mises
(Avg: 75%)
+2.165e+00
+2.058e+00
+1.951e+00
+1.843e+00
+1.736e+00
+1.629e+00
+1.522e+00
+1.415e+00
+1.308e+00
+1.200e+00
+1.093e+00
+9.859e-01
+8.787e-01
+7.715e-01
+6.643e-01
+5.572e-01
+4.500e-01
+3.428e-01
+2.356e-01
+1.284e-01
+2.120e-02
Y
Z
X
S, Mises
(Avg: 75%)
+1.684e+00
+1.601e+00
+1.517e+00
+1.434e+00
+1.351e+00
+1.267e+00
+1.184e+00
+1.101e+00
+1.017e+00
+9.338e-01
+8.505e-01
+7.671e-01
+6.838e-01
+6.004e-01
+5.171e-01
+4.337e-01
+3.504e-01
+2.671e-01
+1.837e-01
+1.004e-01
+1.703e-02
Y
Z
X
S, Mises
(Avg: 75%)
+1.176e+00
+1.117e+00
+1.059e+00
+1.001e+00
+9.424e-01
+8.841e-01
+8.257e-01
+7.673e-01
+7.090e-01
+6.506e-01
+5.923e-01
+5.339e-01
+4.755e-01
+4.172e-01
+3.588e-01
+3.005e-01
+2.421e-01
+1.837e-01
+1.254e-01
+6.703e-02
+8.669e-03
Y
Z
X

Irregular octagon	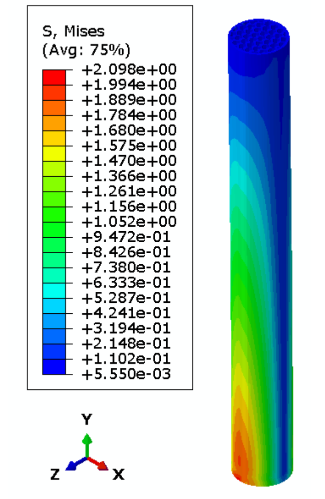	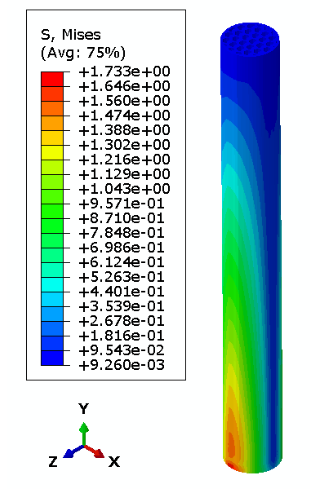	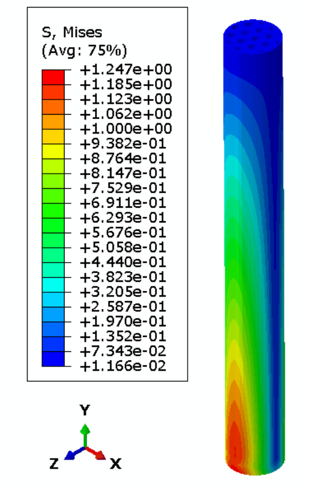
Full filled (100%)		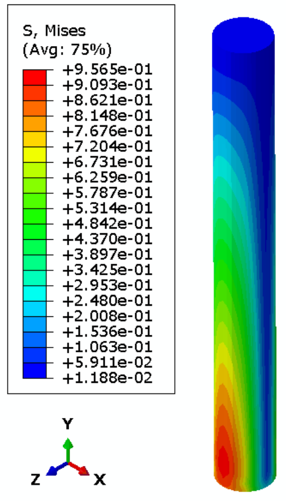	

10. Numerical simulation: it is obligatory to present the used FE mesh for all shapes used in the comparative evaluations. Furthermore, you claim in the text that some parts are compressive and some tensile loaded. Are the tensile and compressive material properties the same. The simulations of the compressive printed parts were done by Karimi et al in https://doi.org/10.3390/app12168018 where also influence of the printed shape of the particular layer was analyzed. What shape of the particular layer have you selected? Perpendicular of the real one?

The Pareto optimization is done correct and there I have no comments.

Answer: We appreciate the valuable feedback. The compressive behavior of PLA was not addressed in this study, but we will incorporate relevant studies discussing this aspect in the revised manuscript.

> *The compressive and tensile behaviors of PLA are usually considered to be the same, but some studies have focused specifically on the compressive behavior of PLA. These studies aim to better understand how PLA responds to compressive forces and how it can be used in applications where compression is a dominant mode of loading, such as in bone tissue engineering and orthopedics [49-50].*

[50] A. Karimi, N. Mole, and T. Pepelnjak, “Numerical Investigation of the Cycling Loading Behavior of 3D-Printed Poly-Lactic Acid (PLA) Cylindrical Lightweight Samples during Compression Testing,” *Appl. Sci.*, vol. 12, no. 16, p. 8018, 2022, doi: 10.3390/APP12168018.

Regarding the shape of the particular layer selected for analysis the rectangular pattern with 60% filling density was chosen based on the results obtained from the two-objective optimization, based in Section 3-2 and Table 5, as mentioned before in the first version of the manuscript, as follows,

> *Considering the interaction of objective functions, in the rectangular pattern with 60% of the filling density, the fatigue life has decreased by only 22%, despite the 39% reduction in mass. In addition, this sample has the shortest distance to the optimal point in the results of two-objective optimization by selecting the third set of objective functions (in Table 5) with an emphasis on the second objective. Based on this, fatigue samples should be printed with a 60% rectangular pattern.*

11. Figure 11 - again only the Von Mises stress is presented. Why?

Answer: To provide a more comprehensive understanding of the stress distribution in the component, we have added Figure 13 in the revised manuscript to include not only the von Mises stress but also the stress components in the directions of the component axis.

> *Figure 13 displays the stress distribution along the component axis in optimized geometries. The contour lines in the figure accurately represent the stress distribution, including stress concentration regions, mirroring the pattern observed in the von-Mises stress. Additionally, the figure distinctly reveals areas with compressive stresses.*

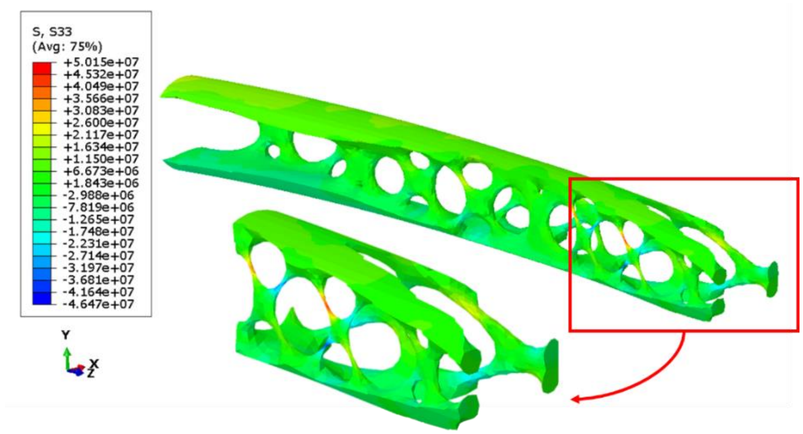

(a)

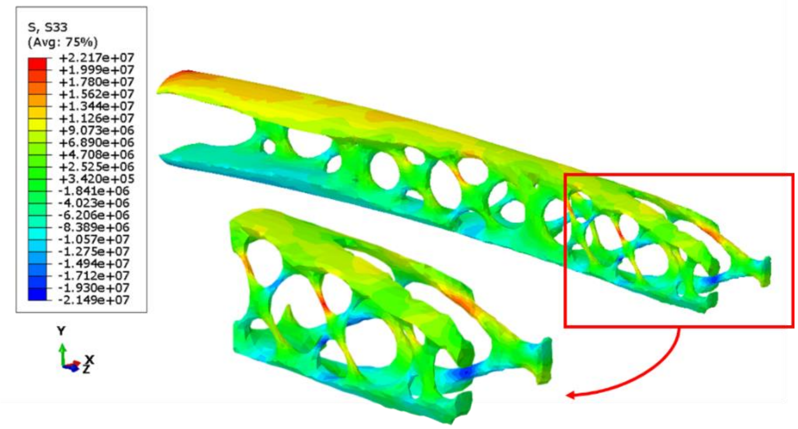

(b)

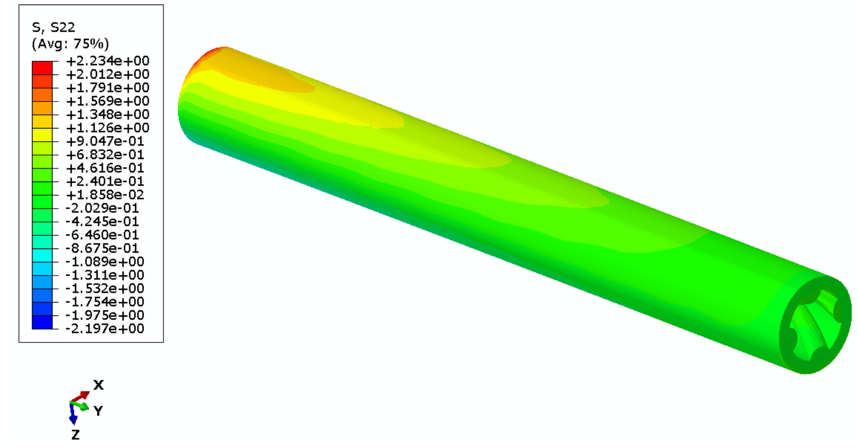

(c)

Fig 13: Stress distribution in the direction of cylinder axis in the optimized component after (a) topology optimization and (b) shape optimization (c) design realization

12. Figure 12 - the scales need to be larger and more readable. From this figure it is also evident that the printing quality is not good. How this quality influence the properties of the real part and even more important - how you can implement those errors in the numerical simulations.

Answer: The scale of Figure 12 (Figure 14 in revised version) is increased in revised version of the article. Additionally, we recognize that the quality of 3D-printed parts can impact their properties. While this study focused on infill patterns, it is crucial to consider other sources of anisotropy, such as changes in printing paths and print parameters, which can affect the final product's quality and properties. These aspects should be considered during the design process.

Based on the microscopic images, different kinds of anisotropy can significantly impact the quality and properties of 3D-printed parts, and the infill patterns examined in this study is one such example. The infill patterns, often referred to as meta-structures, play a crucial role in influencing the properties of the components [56]. The infill patterns, often referred to as meta-structures, play a crucial role in influencing the properties of the components. Additionally, there are other forms of anisotropy that can arise from changes in printing paths or inadequate print parameters. While these factors were not specifically

investigated in this study, it was reported from the porosity analysis that the adhesion between the laminated layers has no significant role to play in flexural properties [6].

13. Figure 13 - the measured distances and angles are vague and not accurate. Comment such results.

Answer: In addressing the respected reviewer's concern about measurement accuracy, we used ImageJ software to measure the lines and angles multiple times, ensuring reliable results within an acceptable tolerance and standard deviation.

As illustrated in Figs 15 and 17, the length of the vertices and width of lines were measured in the microscopic images to visualize dimensional accuracy. Specifically, Fig 15 shows a schematic of the measured line width and line length.

Fig 16 shows the measured values in an irregular octagon as a sample. ImageJ software was used to measure the length and width of the lines, as well as the angle between them. The process repeated three times to ensure accuracy. The results were within a reasonable tolerance based on the difference between the demanded and measured values as well as the standard deviations according to Table 12. The comparison between the average values measured from the microscopic images and corresponding CAD models is done in this table. The purpose of this analysis was to assess how accurately the 3D printer was able to reproduce the intended geometry of the parts, and to identify any potential sources of error or variability in the printing process. By comparing the actual dimensions of the printed parts with the intended dimensions, it is possible to gain insight into the overall quality and reliability of the printing process, and to identify opportunities for improvement.

14. Conclusions: you mention the weight after 50 cycles. Does this changes through the cyclic loading? Furthermore - how is this weight comparable to the ones calculated from the geometry of the CAD model with corresponding material density. What is the real material density of your used material?

Answer: The optimization process experienced multiple design cycles, as shown in S1 Fig, with the final optimized geometry achieved after 50 cycles. Regarding the material properties, the values reported by the manufacturer (density, tensile modulus, yield strength) were obtained through standard tests for solid PLA material, as stated in the revised version.

The optimized geometry in Fig 9 was reached after 50 design cycles. An animation is also included in supporting information (Fig S1) that shows the topology optimization process developments during each design cycle, providing a visual representation of the design process. Such visual support presents a better understanding of the design evolution and the optimization steps taken to arrive at the final design.

The properties of 3D printed parts can differ from those of the raw filament material. For example, while the density of PLA filament was typically reported as 1.24 g/cm^3 in manufacturer datasheets based on the ASTM D792 standard test method, 3D printed parts can have a different density due to their meta-structure. Additionally, the tensile module and yield strength of PLA filament are reported as 3.5 GPa and 60 MPa, respectively, based on ASTM D1238 standard test method. However, 3D printed parts may

exhibit different values for these properties due to the complex interplay of factors such as infill percentage, layer height, printing speed, and other printing parameters.

Reviewer #2:

Dear Authors,

in your interesting manuscript, the following points should be added/changed to further improve it.

Answer: The authors are glad to hear that reviewer found the work interesting and appreciate suggestions on how to improve the article. The comments are carefully considered and the necessary revisions are made to address the reviewer's concerns. These changes were highlighted in the text.

- Introduction: "The robust objective function was weighted mean and variance of the deterministic objective" - what is meant here?

Answer: Thank you for your insightful comments and suggestions regarding our manuscript. We have carefully considered your feedback and made the following revisions to improve the clarity and quality of the article:

> *Agrawal et al. [14] proposed a robust topology optimization method for negative Poisson ratio metamaterials, ensuring stable designs with low variation.*

- Section 2.2: Why were these infill patterns chosen and not, e.g., gyroid, which was found most suitable in experiments dealing with PLA as shape memory polymer?

Answer: We appreciate the respected reviewer's comment regarding our choice of infill patterns in the study. Our decision was based on previous research, which indicated that the infill patterns we selected (cubic, triangular, hexagonal, star, and grid) are commonly used and have been shown to have different mechanical properties. However, we agree that the gyroid pattern could be a promising candidate for future investigations in our research area. We have added a discussion of this point in the revised manuscript.

> *The use of additive manufacturing enables the fabrication of different patterns with varying mechanical properties. The filling parameters have a direct effect on the response of the metamaterials to external loadings. For instance, a recent study [51] on metal functionally graded gyroids employed three design methods (thickness graded, size graded, and uniform) to provide a map of the mechanical properties of stainless-steel scaffolds. In our study, we selected five infill patterns, including honeycomb, rectangular, triangular, irregular octagon [51], and regular octagon [51] to investigate their effect on fatigue properties and mass, and the results suggest that further investigations on other patterns may be necessary, to compare the fatigue behavior. The infill patterns used in this study are shown in Fig 2.*

- Section 2.2: I assume the "thickness of 0.4 mm for each layer" is actually a width of 0.4 mm; else the layer thickness would be identical to the typical nozzle width.

Answer: The respected reviewer is correct that the term "thickness" is inaccurate and we have modified the sentence to read "width of 0.4 mm for each layer" in the revised version of the manuscript.

> *To achieve desired filling densities these geometries were arranged next to each other at different distances. In addition, two solid layers are considered as a perimeter with a width of 0.4 mm for each layer [52].*

- Table 4: What is the difference between "all data" and "mean value"?

Answer: Thank you for your comment regarding Table 4. The "all data" column in Table 4 refers to the S-N diagram that fits all the data points from the fatigue tests conducted at each stress level. On the other hand, the "mean value" column in Table 4 refers to the average of the results at each stress level, which is then used to generate a new S-N diagram. I hope this clarifies the difference between the two columns in Table 4.

> *In this table, fatigue tests were conducted at each stress level three times. Then, an S-N diagram was provided that fitted all the data. Moreover, the average of the results at each stress level was calculated and then again, another S-N diagram was provided.*

- Table 5: All "100" must be "100%". Please think about renaming "Delta W" to "Delta W/W_solid" (ditto for Delta N_f) as your Deltas are relative deviations given in %, while a Delta value is normally assumed to be an absolute difference.

Answer: Thank you for your comment. We have updated Table 5 to rename the mentioned parameters to more accurately reflect their percentage-based nature. Additionally, we have corrected the formatting of '100' to '100%' in the table. We hope that these changes have improved the clarity of the table.

Table 5: The combination of objective functions

Set	f_1	f_2
Set 1	*max:* $N_f = 0.5 \times \left(\frac{\sigma_a}{\sigma_f'}\right)^{\frac{1}{b}}$	*min:* $M = \rho V$
Set 2	*min:* $\Delta N_f\% = \frac{N_{f(solid)} - N_f}{N_{f(solid)}} \times 100\%$	*min:* $\Delta M\% = -\frac{M_{solid} - M}{M_{solid}} \times 100\%$
Set 3	*min:* $S = \Delta N_f\% + \Delta M\%$	*min:* $D = \sqrt{\left(\Delta N_f\%\right)^2 + (\Delta M\% + 100\%)^2}$

- Eqs. 8-11 do not look logically correlated; please check again where you have 1, 2, M, M-1 (or M+1).

Answer: We appreciate the respected reviewer's attention to detail and feedback. We agree with you that the equations 8-11 were not clearly correlated with the introduced parameters. To clarify this issue, we have modified the manuscript and introduced the parameters as follows:

> Let M denote the number of Pareto non-inferior solutions, and let x^m $(\mathrm{m} = 1,2,\ldots,\mathrm{M})$ denote the set of these solutions.

- Fig. 4: What do the green lines mean?

Answer: In response to the respected reviewer's comment regarding the meaning of the green lines in Fig. 4, we would like to clarify that these lines represent the desired part of the print samples that were evaluated through inspection in the FESEM. This information has been added to the revised manuscript, and we thank the reviewer for bringing this to our attention.

> *The green lines in Fig 4 represent the desired part of the print samples that was cut and evaluated through inspection in the FESEM.*

- "It should be noted that due to the complex shapes, samples were printed 5 times larger than CAD models based on the capability of machine." - why? Which dimensions do the samples have and how many filled top/bottom layers and perimeters? And why was no commercially available printer used?

Answer: To address the reviewer's comment, the decision to print the samples at a larger scale was made to leverage the capabilities of an industrial 3D printer, enabling accurate and precise printing of complex lattice structures. The chosen infill patterns were designed in the CAD model and printed with 100% infill for clear visualization of the print path during subsequent experimental testing.

> *To ensure optimal results and utilize the capabilities of an industrial-duty 3D-printer machine, the samples were printed at a scale of 5 times larger than the original CAD models. This approach allowed to produce lattice structures with circular sections and a diameter of 45 millimeters, which would have been challenging to achieve with smaller scales. Since the infill patterns were designed in the CAD model, the samples were printed with 100% infill and no perimeters or upper/lower layers, allowing for clear visualization of the print path.*
> *The samples were printed with 100% infill and no top and bottom solid layers printed, allowing for clear visualization of the print path.*

- Fig. 6b: The y-axis shows the mass, not the weight of the samples. What do you mean in the corresponding text with "unpredictable relationship" - actually this is quite clear and logical.

Answer: The reviewer's correction regarding the use of the term "mass" instead of "weight" has been acknowledged and will be implemented in the paper. Additionally, the following discussion about mass of different filling pattern were added to the revised article, as follows,

> *Based on the results shown in Fig 6(b), the mass of the honeycomb and rectangular lattice structures was nearly identical at various infill densities, with the rectangular pattern being slightly heavier at 40% infill. In addition, the triangular pattern exhibited less mass compared to the other three patterns. The irregular octagon, regular octagon, and rectangular patterns were the heaviest at 40%, 60%, and 80% infills, respectively.*

- Fig. 7: Where is the difference between blue and green points?

Answer: The green and blue points in Fig 7 represent different orientations of the lattice structures during printing. The green points correspond to vertical specimens, where the lattice is printed in a vertical orientation. This issue could be seen in the figure legend. Moreover, this is highlighted in the revised version of the article to address the respected reviewer's comment.

> *In Fig 7, the Pareto front indicates that there was not much change in the fatigue lifetime of vertical specimens (green points) even with a severe reduction in mass. On the other hand, the horizontal orientation (blue points) resulted in layer lines being placed horizontally in the direction of the bending force, making it the optimal orientation for mechanical strength. Despite the need for support during printing, this orientation provides the best mechanical strength.*

- Fig. 9: Does this optimized structure take the strong anisotropies in FDM printing into account?

Answer: In response to the respected reviewer's question regarding whether the optimized structure in Fig. 9 takes into account the strong anisotropies in FDM printing, it should be noted that while the infill patterns (meta-structures) were specifically designed to improve the mechanical properties of the printed parts, other forms of anisotropy arising from changes in printing paths or inadequate print parameters were not specifically investigated in this study. However, the importance of considering these factors during the design stage was emphasized in the revised version of the article.

> *Based on the microscopic images, different kinds of anisotropy can significantly impact the quality and properties of 3D-printed parts, and the infill patterns examined in this study is one such example [56]. The infill patterns, often referred to as meta-structures, play a crucial role in influencing the properties of the components. Additionally, there are other forms of anisotropy that can arise from changes in printing paths or inadequate print parameters. While these factors were not specifically investigated in this study, they should not be disregarded during the design stage, as they can have a notable impact on the performance of the final products.*

- Fig. 11: How can the optimized shape be FDM printed?

Answer: The printed specimen, demonstrating the optimized shape, has been included and visually depicted in Figure 11.

> *The printed specimen in Fig 11 exhibits the desired geometry and structural features as intended in this design. This physical realization served as a tangible representation of the proposed model and validated the effectiveness of the present design process. It provided concrete evidence that the optimized design can be successfully translated into a physical object through 3D printing.*

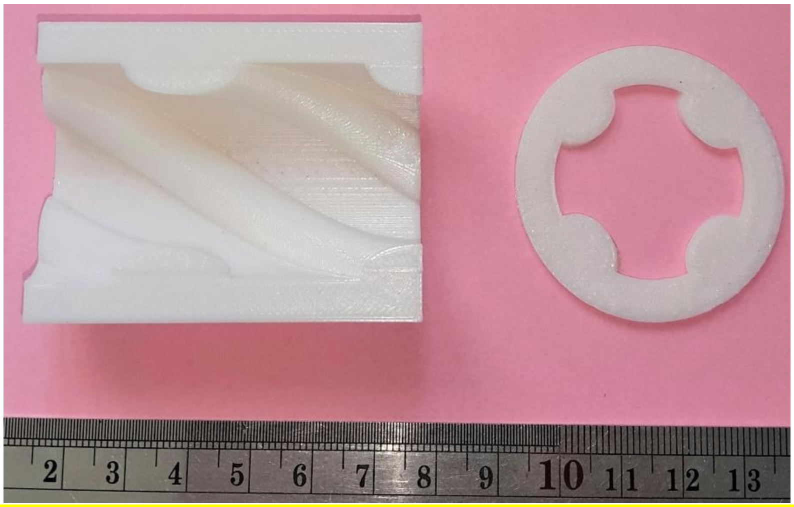

Fig 11: The designed sample printed based on the result of topology optimization

- Table 10: The second column is "Mass (g)".

Answer: The reviewer's correction about using "mass" instead of "weight" in our paper has been acknowledged, and the necessary updates will be made to reflect this correction.

- Fig. 12: How are the air voids visible here taken into account in the simulation?

Answer: Air voids in 3D-printed parts, including those shown in Fig. 12, can impact quality and properties. This study examined infill patterns, but other voids from printing paths or parameters also affect performance. Design considerations must address these factors for optimal product performance.

Based on the microscopic images, different kinds of voids can significantly impact the quality and properties of 3D-printed parts, and the infill patterns examined in this study is one such example [56]. The infill patterns, often referred to as meta-structures, play a crucial role in influencing the properties of the components. Additionally, there are other forms of defects that can arise from changes in printing paths or inadequate print parameters. While these factors were not specifically investigated in this study, they should not be disregarded during the design stage, as they can have a notable impact on the performance of the final products.

- Fig. 14: Which sorts of defects do you see in (b) and (c)? At least there are no air voids.

Answer: Higher magnification image presented in Fig 14(b) (Fig 16(b) in the revised version of the article), provided a clearer view of the defect. Based on the images presented in Fig 14 (Fig 16 in the revised version of the article), the defects observed are likely due to inconsistent fusion caused by imbalanced extrusion feed rate, extrusion temperature, and printing speed. These factors can lead to weakened mechanical properties and compromised performance of the printed part. It is crucial to consider these factors and optimize printing parameters to minimize defects and achieve the desired properties in 3D-printed parts.

As observed in Fig 16(b), some small spherical-shaped pores may be generated during the fabrication of the filament, which can negatively impact the part's mechanical properties. Additionally, the extrusion feed rate plays a critical role in determining the quality of printed parts, as shown in Fig 16(c). If the extrusion feed rate is not balanced with the extrusion temperature and printing speed, inconsistent fusion can occur, resulting in defects. Another parameter that can affect the quality of printed parts is the distance between the nozzle and the build platform [70]. This distance can impact the adhesion between the layers and can cause defects similar to those shown in Fig 16(c). It is essential to consider these factors during the design and optimization of 3D-printed parts to achieve the desired mechanical properties and avoid potential defects.

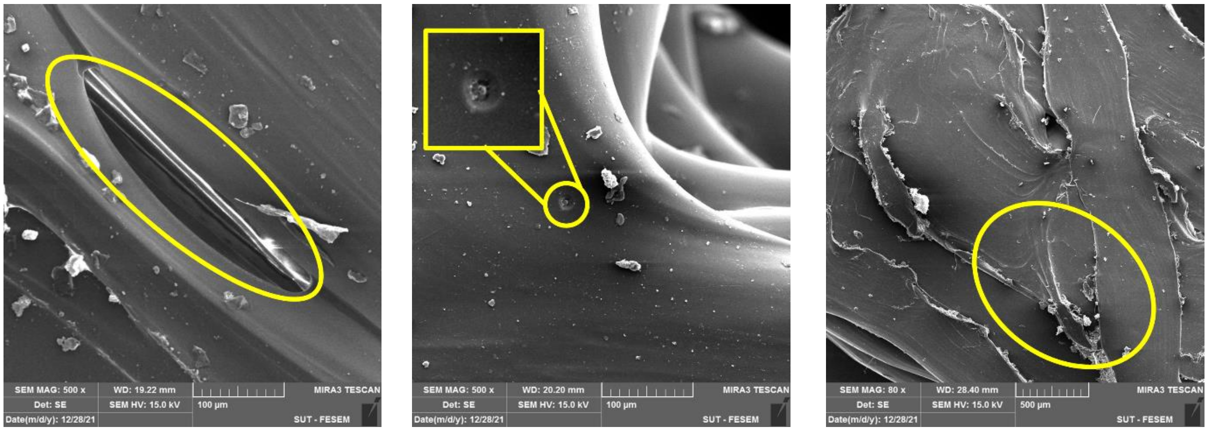

(a) (b) (c)

Fig 16: The defects observed in the samples with (a) 60% infill regular octagon, (b) 80% infill rectangular, and (c) 60% infill irregular octagon

- Fig. 15: The length unit is wrong. Length and angle values have much too high accuracy; you can definitely not measure with 6 significant digits accuracy on such an image.

Answer: In the revised version, we have corrected the length unit in Fig 15 (Fig 17 in the revised article). For measurement in Fig 17, ImageJ software was used to measure length, width, and angle. The process was repeated three times for accuracy, and standard deviations were calculated. The measured values in Table 12 demonstrate a reasonable tolerance and effectively represent the shape and dimensions of the irregular octagon in Fig 17.

Fig 17 shows the measured values in an irregular octagon as a sample. ImageJ software was used to measure the length and width of the lines, as well as the angle between them. The process repeated three times to ensure accuracy. The results were within a reasonable tolerance based on the difference between the demanded and measured values as well as the standard deviations according to Table 12.

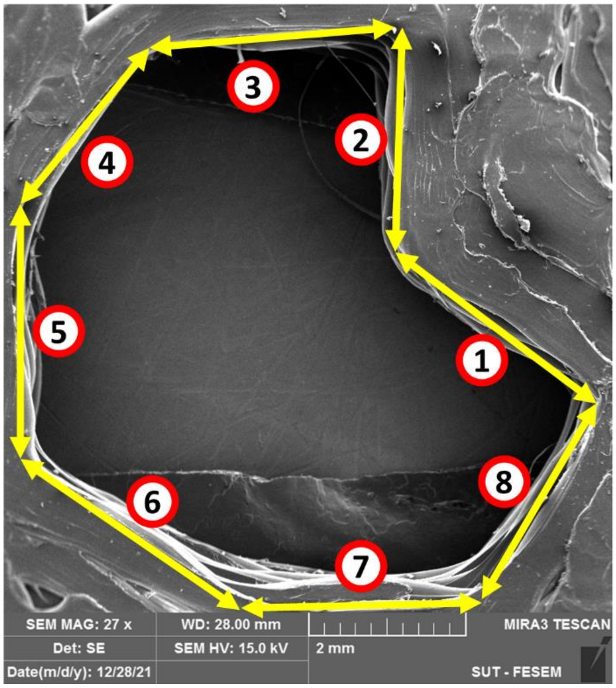

ID	Length (mm)	Angle (degree)
1	3.329	146.4
2	3.087	85.7
3	3.526	3.4
4	3.067	38.8
5	3.463	92.6
6	3.556	145.9
7	3.300	0.2
8	3.402	60.0

Fig 17: The dimensional accuracy of the irregular octagon, measured from FESEM micrographs of the 3D-printed PLA with 60% of infill

- Table 11: Where do these values stem from? Where are the units? Where do the standard deviations belong to? How should a printed line, typically of 0.4 mm width, have a demanded width of 2.75 mm etc., and what is meant with the line length? Besides, it would be good to correlate the measured infills (surely not measured with this accuracy) with the measured sample masses.

Answer: In response to the reviewer's comment, the measured line width in Table 11 (Table 12 in the revised version) represents the width of the printed structure's walls, measured using ImageJ software. Multiple measurements were taken at different locations to ensure accuracy and consistency. Following explanations were added in the revised version of the article to address the comment,

> *As illustrated in Fig 15 and 17, the length of the vertices and width of lines were measured in the microscopic images to visualize dimensional accuracy. Specifically, Fig 15 shows a schematic of the measured line width and line length. The measured line width in this picture does not refer to the actual line width produced by the nozzle. Rather, it is the width of the walls in the structure, which may consist of multiple print paths. Fig 16 shows the measured values in an irregular octagon as a sample. ImageJ software was used to measure the length and width of the lines, as well as the angle between them. The process repeated three times to ensure accuracy. The results were within a reasonable tolerance based on the difference between the demanded and measured values as well as the standard deviations according to Table 12. The comparison between the average values measured from the microscopic images and corresponding CAD models is done in this table. The purpose of this analysis was to assess how accurately the 3D printer was able to reproduce the intended geometry of the parts, and to identify any potential sources of*

error or variability in the printing process. By comparing the actual dimensions of the printed parts with the intended dimensions, it is possible to gain insight into the overall quality and reliability of the printing process, and to identify opportunities for improvement.

- "Based on the scatterplot in Figure 16, a strong positive and linear relationship between the two variables" - which ones?

Answer: In response to the respected Reviewer's comment on Figure 16 (Fig 18 in revised version), a clear positive and linear relationship is between demanded and measured line widths and lengths.

Based on the scatterplot presented in Fig 18, it is evident that there exists a strong positive and linear relationship between the demanded and measured line widths (Fig 18(a)) as well as the demanded and measured line lengths (Fig 18(b)). This indicates that as the demanded line width or line length increases, there is a corresponding increase in the measured values, highlighting the consistent and proportional behavior between the two variables in the printing process.

- Fig. 16: all texts are too small to be readable.

Answer: Figure 16 (Fig 18 in revised version) modified to improve text readability.

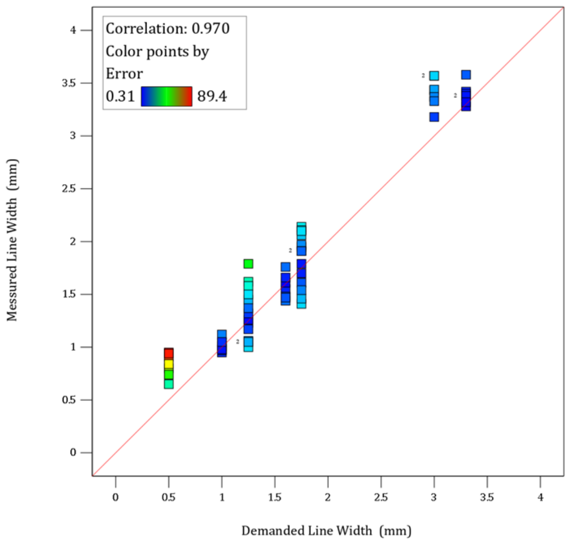

(a)

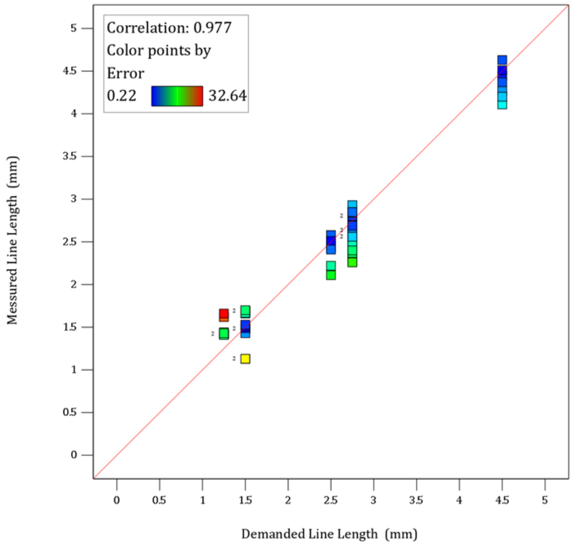

(b)

Fig 18: The scatter plot of demanded values versus the measured data for (a) the line length and (b) the line width

- Conclusion: The information that a sample with infill density 60% is 39% lighter than the 100% sample sounds logical, but does not fit to the measured infill percentages according to Table 11. It is thus really necessary to defined how you measured this value.

Answer: In the revised version of the article, it is clarified that the infill percentages were measured using ImageJ software based on microscopic images, providing accurate insights into the amount of infill material in each sample. The weight reduction comparison is based on calculated weight values considering material density and measured volumes in the CAD model.

> *The comparison of weight reduction in this study is conducted by considering the calculated weight values, taking into account the theoretical density of the material and the measured volumes of the samples in the CAD model. By utilizing these parameters, the weight of the objects can be estimated.*
>
> *Fig 16 shows the measured values in an irregular octagon as a sample. ImageJ software was used to measure the length and width of the lines, as well as the angle between them. The process repeated three times to ensure accuracy. The results were within a reasonable tolerance based on the difference between the demanded and measured values as well as the standard deviations according to Table 12. The comparison between the average values measured from the microscopic images and corresponding CAD models is done in this table. The purpose of this analysis was to assess how accurately the 3D printer was able to reproduce the intended geometry of the parts, and to identify any potential sources of error or variability in the printing process. By comparing the actual dimensions of the printed parts with the intended dimensions, it is possible to gain insight into the overall quality and reliability of the printing process, and to identify opportunities for improvement.*

The conclusion part is also modified as follows,

The study introduced three sets of objective functions for design purposes, considering mass changes, fatigue lifetime changes, and the distance from the optimal point. The optimal solution was found to be the horizontally printed rectangular filling pattern with a 60% infill density. This pattern achieved a 39% reduction in weight while experiencing only a 22% decrease in fatigue lifetime. The mass reduction was determined through software analysis of designed CAD models, while the infill density was assessed using imageJ software.

- The conclusion is too long.

Answer: Thank you for your feedback. We worked on maintaining a concise and clear conclusion that effectively summarizes the study's key findings and implications.

This research used multi-objective optimization to determine optimal parameters for PLA cylinders, considering fatigue lifetime, mass, and infill patterns. Structural topology optimization was conducted using a two-objective algorithm. Additionally, FESEM analysis ensured dimensional accuracy, and the influence of inherent defects in the FDM process was considered. The results are listed below:

- *The stress analysis results showed that although the triangular pattern had the lowest mass in all cases, irregular octagon, rectangular and regular octagon had the lowest stresses in 40%, 60%, and 80% of the infill, respectively.*
- *The study introduced three sets of objective functions for design purposes, considering mass changes, fatigue lifetime changes, and the distance from the optimal point. The optimal solution was found to be the horizontally printed rectangular filling pattern with a 60% infill density. This pattern achieved a 39% reduction in weight while experiencing only a 22% decrease in fatigue lifetime. The mass reduction was determined through software analysis of designed CAD models, while the infill density was assessed using the ImageJ software.*
- *Topology optimization in Dassault Systems software proposed a metamaterial structure to minimize mass and strain energy under stress constraints. Achieved results: mass of 1.1767g, strain energy of 0.056 J (after 50 cycles), maximum von-Mises stress of 97.08 MPa. Microscopic inspections studied manufacturing defects and their impact on topology optimization.*
- *Shape optimization was performed to reduce stress concentration by displacing boundary nodes. Maximum von-Mises stress decreased to 44.30 MPa after 15 design cycles, leading to increased fatigue lifetime by 527 and 79 cycles compared to topology optimization in vertical and horizontal samples, respectively.*
- *Finally, a design realization step was performed, and a new geometry was introduced that has 96.70% lower stress and 201.69% higher mass compared with topology-optimized geometry. This is while this geometry is lighter than 60% infill samples with different patterns.*
- *Based on FESEM images, the rectangular pattern has the best regularity, while the regular octagon pattern was the most irregular.*

- *The line spacing is significant, especially in the honeycomb pattern, and should be controlled by printing temperature.*
- *The scatterplot shows a strong positive and linear relationship between the demanded and measured line length and line width.*
- *Inaccuracy in the position of the nozzle increases with the decrease in filling percentage and, therefore, the thickness of the walls. However, the rectangular sample with 40% infill and Regular Octagon with 60% infill was the worst parameters.*

Reviewer #3:

The author has done good work. The work is related to "multi-objective numerical optimization of 3D-printed polylactic acid bio-metamaterial based on topology, filling pattern, and infill density via fatigue lifetime and weight" and has provided good insights on fatigue life time for the Additively manufactured printed prototypes. Here are some suggestions which the author can incorporate in the manuscript for improvement in the output of this work.

Answer: The authors express their gratitude to the respected reviewer for their valuable comments. Each comment has been carefully addressed in the revised article, and corresponding responses have been provided in the subsequent paragraphs. The changes were also highlighted in the revised article.

1. Author should provide scope of the work and literature gap as separate heading for better understanding

Answer: Thank you for the suggestion. The journal's template does not include a separate section for the scope of the work and literature gap. However, we have addressed and discussed these aspects at the end of the Introduction.

The article discusses the multi-objective numerical optimization of 3D-printed polylactic acid (PLA) bio-metamaterial. The optimization takes into account the factors of topology, filling pattern, and infill density in order to improve the fatigue lifetime and mass of the material. Therefore, the novelties of the study are as follows,

- *The use of multi-objective optimization to find the optimum value of topology, filling pattern, and infill density for 3D-printed polylactic acid bio-metamaterial.*
- *The consideration of both fatigue lifetime and mass in the optimization process.*
- *The use of field emission scanning electron microscopy (FESEM) images to investigate the dimensional accuracy of 3D-printed components.*

2. What is bio-metamaterial? Bio material is separate term whereas Meta material is something related to design-based objective of materials.

Answer: In the revised version of the article, bio-metamaterials are defined as follow,

Bio-metamaterials, designed with geometrical structures at smaller scales, mimic the properties of living tissues and are applied in orthopedic biomaterials for bone tissue regeneration [11].

3. Figure 6a shows the 100% infill density part has shown stress capacity <10MPa. But the original value for PLA material lies somewhere between 30-70MPa. Why the predicted value is many times lower than the actual value?

Answer: The reviewer correctly points out that the original yield strength of PLA material is higher than the stress capacity observed in Figure 6a. However, in high cycle fatigue testing, it is necessary to avoid stress levels exceeding the yield strength to prevent plastic deformation or permanent damage. Our study intentionally operated within a stress range below the yield strength to accurately evaluate the fatigue performance and durability of the 3D-printed parts without compromising their structural integrity.

In the rotating-bending test, a common method used to evaluate the high cycle fatigue behavior of materials, the specimen is subjected to cyclic loading through rotational motion. During the rotating-bending test, the applied stress levels are typically kept below the yield strength of the material. This ensures that the deformation experienced by the specimen remains within the elastic range (<70 MPa [47]), where the material can fully recover its original shape after each cycle. The focus is on assessing the material performance to withstand cyclic loading without undergoing plastic deformation or failure.

4. Whereas Figure 6b is not important as it is well established fact that when density increases the weight will increase.

Answer: In response to the respected reviewer's comment, the revised article includes the following information,

Based on the results shown in Fig 6(b), the mass of the honeycomb and rectangular lattice structures was nearly identical at various infill densities, with the rectangular pattern being slightly heavier at 40% infill. In addition, the triangular pattern exhibited less mass compared to the other three patterns. The irregular octagon, regular octagon, and rectangular patterns were the heaviest at 40%, 60%, and 80% infills, respectively

5. No practical work has been performed other than mathematical relations and numerical simulation. You should 3D print any material and test it with your optimized condition and verify the predicted model using real environment condition?

Answer:

In response to the reviewer's comment the following information is added to the revised version of the article,

In this research, the emphasis was primarily on numerical simulation and optimization of 3D-printed materials. The results were utilized to determine the parameters for experimental tests, the data of which has already been published [59-60].

6. One real 3D printed object based on optimized or suggested model should be printed for PLA material and the model should be verified to find the accuracy of the model.

Answer: We thank the reviewer for their suggestion. We have followed their advice and printed a real 3D object based on the optimized model for PLA material. We have also verified the accuracy of the model by comparing the dimensions of the printed object with the simulation results. We have added this information and a new figure (Figure 11) to the revised manuscript to demonstrate the validity of our approach.

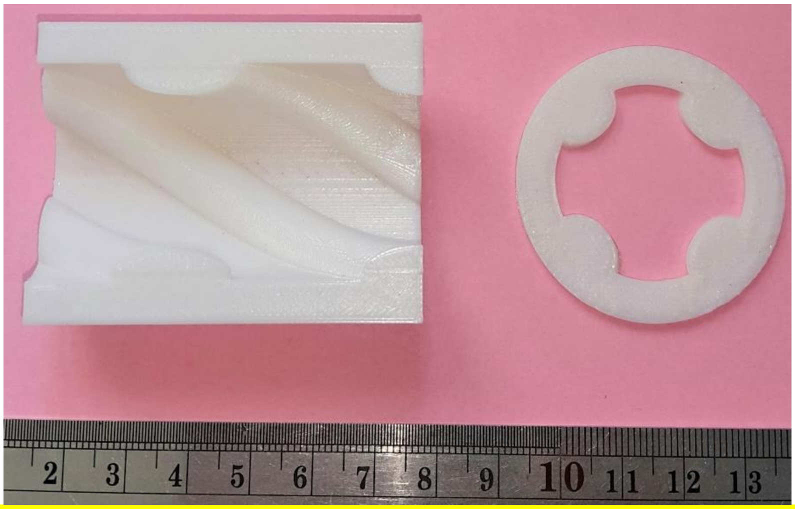

Fig 11: The designed sample printed based on the result of topology optimization

7. Previous literature work or result should be compared with the proposed model result for validation purpose along with suitable graph of validation.

Answer: In response to the reviewer's comment, following discussions were added to the revised version of article,

> *The literature supports that the outer shell affects bending strength. Samples with ABS: TPU: ABS sandwiched structure showed higher flexural strength due to ABS material in outer layers, aiding compression and expansion during loading [5].*

8. Following literature may be added to enhance the literature review

1. Kumar S, Singh I, Kumar D, Yahya MY, Rahimian Koloor SS. Mechanical and Morphological Characterizations of Laminated Object Manufactured 3D Printed Biodegradable Poly (lactic) acid with Various Physical Configurations. Journal of Marine Science and Engineering. 2022 Dec;10(12):1954.
2. Kumar S, Singh I, R. Koloor SS, Kumar D, Yahya MY. On Laminated Object Manufactured FDM-Printed ABS/TPU Multimaterial Specimens: An Insight into Mechanical and Morphological Characteristics. Polymers. 2022 Sep 28;14(19):4066.
3. Kumar S, Singh R, Singh TP, Batish A. Comparison of mechanical and morphological properties of 3-D printed functional prototypes: Multi and hybrid blended thermoplastic matrix. Journal of Thermoplastic Composite Materials. 2022 May;35(5):692-707.
4. Kumar S, Singh R, Batish A, Singh TP. Additive manufacturing of smart materials exhibiting 4-D properties: a state of art review. Journal of Thermoplastic Composite Materials. 2022 Sep;35(9):1358-81.

Answer: To address the respected reviewer's comment, the recommended studies were considered and have incorporated related works that align with the focus and scope of our research in the revised version of the manuscript.

[5] S. Kumar, I. Singh, S. S. R. Koloor, D. Kumar, and M. Y. Yahya, "On Laminated Object Manufactured FDM-Printed ABS/TPU Multimaterial Specimens: An Insight into Mechanical and Morphological Characteristics," *Polymers (Basel).*, vol. 14, no. 19, p. 4066, 2022, doi: 10.3390/POLYM14194066.

[6] S. Kumar, I. Singh, D. Kumar, M. Y. Yahya, and S. S. Rahimian Koloor, “Mechanical and Morphological Characterizations of Laminated Object Manufactured 3D Printed Biodegradable Poly(lactic)acid with Various Physical Configurations,” *J. Mar. Sci. Eng.*, vol. 10, no. 12, p. 1954, 2022, doi: 10.3390/JMSE10121954.

[10] S. Kumar, R. Singh, A. Batish, and T. P. Singh, “Additive manufacturing of smart materials exhibiting 4-D properties: A state of art review,” vol. 35, no. 9, pp. 1358–1381, 2019, doi: 10.1177/0892705719895052.

Reviewer #4:

The manuscript represents good potentials for readers. All section represents technically correct data. Analysis is technically sound. Data is supported by relevant literature. Authors have taken nice efforts to fulfil requirements of Journal.

Answer: Thank you for your positive feedback and recognition of the manuscript's potential and technical accuracy. We are glad to hear that the analysis is deemed technically sound and supported by relevant literature. We greatly appreciate your acknowledgment of our efforts in meeting the requirements of the journal. Your positive evaluation encourages us to continue our work and strive for excellence.

Reviewer #5:

The study is well conducted and results have been theoretically discussed. This manuscript could be accepted in its current form. The execution of the experimentation and testing is at par with the current research requisites.

Answer: Thank you for your positive evaluation of the study. We are pleased to hear that the manuscript's content and results have been well conducted and theoretically discussed.

Reviewer #6:

The focus of this work is studying optimization of 3D-printed polylactic. The present work does not include any novel materials or methods. This manuscript is insufficient of novelty or significant contribution for publication as all materials and methods have been published in the last 3 years, see the references. In conclusion, the innovation of the manuscript is very scarce.

Answer: We thank the reviewer for comments and concerns. As we stated at the end of the introduction, our main novelties are as follows,

> *The article discusses the multi-objective numerical optimization of 3D-printed polylactic acid (PLA) bio-metamaterial. The optimization takes into account the factors of topology, filling pattern, and infill density in order to improve the fatigue lifetime and mass of the material. Therefore, the novelties of the study are as follows,*
>
> - *The use of multi-objective optimization to find the optimum value of topology, filling pattern, and infill density for 3D-printed polylactic acid bio-metamaterial.*
> - *The consideration of both fatigue lifetime and mass in the optimization process.*
> - *The use of field emission scanning electron microscopy (FESEM) images to investigate the dimensional accuracy of 3D-printed components.*

Additionally, the conclusion of the study has been modified to emphasize the significance of the innovations,

> *This research used multi-objective optimization to determine optimal parameters for PLA cylinders, considering fatigue lifetime, mass, and infill patterns. Structural topology optimization was conducted using a two-objective algorithm. Additionally, FESEM analysis ensured dimensional accuracy, and the influence of inherent defects in the FDM process was considered.*

Apart from the lack of contribution, it has two major issues that are lack of any design of experiments for 3D printing process and loading in a cyclic manner for this particular application. That is why we see quick failure under loading. I would strongly suggest the authors to carry out a proper design of experiments to see the effects of printing parameters like temperature of nozzle, speed of print head, fibre orientation, layers arrangement, infill pattern, etc. on the performance of the structure under mechanical loading. As it is under cyclic loading in the real application, the mechanical loading modelling must be cyclic as well.

A new version of the manuscript with all major fundamental changes could be re-submitted for review.

Answer: The authors agree that experimental testing and design of experiments are important aspects to consider in the 3D-printing process and the mechanical loading of the material. However, as we stated in our manuscript, in this research, the focus was on numerical simulation and optimization of 3D-printed materials, with the obtained results guiding the parameters for subsequent experimental tests, the details of which have already been published [59-60]. We hope that this will satisfy the reviewer's concerns and expectations.

In order to address the respected reviewer's comment, the following paragraph for the future works is added to the revised article, as follows,

> *Designing for high-performance in additive manufacturing, despite its limitations, requires exploring various unit cells and meta-structures to identify patterns that interact effectively with material behavior. In this research, the emphasis was primarily on*

numerical simulation and optimization of 3D-printed materials. The results were utilized to determine the parameters for experimental tests, the data of which has already been published [59-60]. For further investigations, the following subjects are suggested,

- *Path planning and defining the route that the print head takes*
- *Providing topology optimization algorithms to achieve desired properties for specific applications*
- *Using exactly the material properties of the meta-structure and anisotropy (considering the 3D printing factors) for the lifetime prediction*
- *Examine the fatigue lifetime experimentally for the topology-optimized samples.*
