# Supplementary material for: Multi-objective numerical optimization of 3D-printed polylactic acid bio-metamaterial based on topology, filling pattern, and infill density via fatigue lifetime and mass
Source: PLoS One. 2023 Sep 27;18(9):e0291021. doi: 10.1371/journal.pone.0291021 (PMC10529563; doi:10.1371/journal.pone.0291021)
Supplement: Attachment — Submitted filename: PLOS comments for author.docx [file pone.0291021.s003.docx]

Comments to the author

The author has done good work. The work is related to “**multi-objective numerical optimization of 3D-printed polylactic acid bio-metamaterial based on topology, filling pattern, and infill density via fatigue lifetime and weight**” and has provided good insights on fatigue life time for the Additively manufactured printed prototypes. Here are some suggestions which the author can incorporate in the manuscript for improvement in the output of this work.

1. Author should provide scope of the work and literature gap as separate heading for better understanding
2. What is bio-metamaterial? Bio material is separate term whereas Meta material is something related to design-based objective of materials.
3. Figure 6a shows the 100% infill density part has shown stress capacity <10MPa. But the original value for PLA material lies somewhere between 30-70MPa. Why the predicted value is many times lower than the actual value?
4. Whereas Figure 6b is not important as it is well established fact that when density increases the weight will increase.
5. No practical work has been performed other than mathematical relations and numerical simulation. You should 3D print any material and test it with your optimized condition and verify the predicted model using real environment condition?
6. One real 3D printed object based on optimized or suggested model should be printed for PLA material and the model should be verified to find the accuracy of the model.
7. Previous literature work or result should be compared with the proposed model result for validation purpose along with suitable graph of validation.
8. Following literature may be added to enhance the literature review
1. Kumar S, Singh I, Kumar D, Yahya MY, Rahimian Koloor SS. Mechanical and Morphological Characterizations of Laminated Object Manufactured 3D Printed Biodegradable Poly (lactic) acid with Various Physical Configurations. Journal of Marine Science and Engineering. 2022 Dec;10(12):1954.
2. Kumar S, Singh I, R. Koloor SS, Kumar D, Yahya MY. On Laminated Object Manufactured FDM-Printed ABS/TPU Multimaterial Specimens: An Insight into Mechanical and Morphological Characteristics. Polymers. 2022 Sep 28;14(19):4066.
3. Kumar S, Singh R, Singh TP, Batish A. Comparison of mechanical and morphological properties of 3-D printed functional prototypes: Multi and hybrid blended thermoplastic matrix. Journal of Thermoplastic Composite Materials. 2022 May;35(5):692-707.
4. Kumar S, Singh R, Batish A, Singh TP. Additive manufacturing of smart materials exhibiting 4-D properties: a state of art review. Journal of Thermoplastic Composite Materials. 2022 Sep;35(9):1358-81.
