# Supplementary material for: Multi-objective numerical optimization of 3D-printed polylactic acid bio-metamaterial based on topology, filling pattern, and infill density via fatigue lifetime and mass
Source: PLoS One. 2023 Sep 27;18(9):e0291021. doi: 10.1371/journal.pone.0291021 (PMC10529563; doi:10.1371/journal.pone.0291021)
Supplement: Attachment — Submitted filename: Answers.docx [file pone.0291021.s004.docx]

**Dear Editor-in-Chief,**

PLOS ONE

Authors thank you for giving me the opportunity to revise my paper titled “Multi-objective numerical optimization of 3D-printed polylactic acid bio-metamaterial based on topology, filling pattern, and infill density via fatigue lifetime and mass”. I appreciate the valuable feedback and comments provided by the reviewers, which have helped me to improve the quality and clarity of my manuscript. In this revision letter, I will address each of the reviewers' comments and explain how I have addressed them in the revised version of the paper. I hope that the changes I have made will sufficiently address the concerns raised and that the revised manuscript will be suitable for publication in PLOS ONE.

Regards,

**M. Azadi, PhD.**

Faculty of Mechanical Engineering, Semnan University, Semnan, Iran

**Journal requirements:**

When submitting your revision, we need you to address these additional requirements.

**Answer:** In order to address these additional requirements, we have carefully reviewed the manuscript and made the necessary changes to ensure that it meets all of the submission guidelines and requirements of PLOS ONE. We appreciate the editor's feedback and will work diligently to address all of the necessary revisions.

1. Please ensure that your manuscript meets PLOS ONE's style requirements, including those for file naming. The PLOS ONE style templates can be found at

https://journals.plos.org/plosone/s/file?id=wjVg/PLOSOne_formatting_sample_main_body.pdf and

https://journals.plos.org/plosone/s/file?id=ba62/PLOSOne_formatting_sample_title_authors_affiliations.pdf

**Answer:** We have reviewed and updated our manuscript accordingly, including file naming and formatting, using the PLOS ONE style templates provided. Please let us know if there are any further issues with the formatting of our manuscript.

2. Please note that PLOS ONE has specific guidelines on code sharing for submissions in which author-generated code underpins the findings in the manuscript. In these cases, all author-generated code must be made available without restrictions upon publication of the work. Please review our guidelines at https://journals.plos.org/plosone/s/materials-and-software-sharing#loc-sharing-code and ensure that your code is shared in a way that follows best practice and facilitates reproducibility and reuse.

**Answer:** We confirm that the findings in our manuscript were not underpinned by any author-generated code, and hence, we have no code to share. We have reviewed the PLOS ONE guidelines on code sharing and ensured that we have followed best practices for the materials and software used in our study.

3. We note that the grant information you provided in the ‘Funding Information’ and ‘Financial Disclosure’ sections do not match.

When you resubmit, please ensure that you provide the correct grant numbers for the awards you received for your study in the ‘Funding Information’ section.

**Answer:** Thank you for bringing this to our attention. We have reviewed our records and have corrected the grant number in the 'Funding Information' section to accurately reflect the grant we received from our funding agency. We appreciate your attention to detail and will ensure that all information is accurate in our revised submission.

4. Thank you for stating the following in the Acknowledgments Section of your manuscript:

“The authors would like to acknowledge the financial support of the Iran Small Industries and Industrial Parks Organization (ISIPO) for this project under grant number of 23607.”

We note that you have provided funding information that is currently declared in your Funding Statement. However, funding information should not appear in the Acknowledgments section or other areas of your manuscript. We will only publish funding information present in the Funding Statement section of the online submission form.

Please remove any funding-related text from the manuscript and let us know how you would like to update your Funding Statement. Currently, your Funding Statement reads as follows:

“The authors would like to acknowledge the financial support of the Iran Small Industries and Industrial Parks Organization (ISIPO) for this project under grant number of 23607.”

Please include your amended statements within your cover letter; we will change the online submission form on your behalf.

**Answer:** Thank you for bringing this to our attention. We have removed the funding-related text from the Acknowledgments section and have updated our Funding Statement accordingly in the cover letter as requested. Regarding the Funding Statement section of the online submission form, I would like to amend it as follows:

*“The research reported in this manuscript was financially supported by the Iran Small Industries and Industrial Parks Organization (ISIPO) under a grant number of 23607.”*

5. Please include captions for your Supporting Information files at the end of your manuscript, and update any in-text citations to match accordingly. Please see our Supporting Information guidelines for more information: http://journals.plos.org/plosone/s/supporting-information.

**Answer:** Thank you for your comment. We included captions for our Supporting Information files at the end of the manuscript and update any in-text citations to match accordingly, following the guidelines provided by PLOS ONE.

*The optimized geometry in Fig 9 is reached after 50 design cycles. An animation is also included in supporting information (Fig S1) that shows the topology optimization process developments during each design cycle, providing a visual representation of the design process. Such visual support presents a better understanding of the design evolution and the optimization steps taken to arrive at the final design.*

*"Fig. S1: The GIF file for the developments during the topology optimization"*

6. We note that you have indicated that data from this study are available upon request. PLOS only allows data to be available upon request if there are legal or ethical restrictions on sharing data publicly. For more information on unacceptable data access restrictions, please see http://journals.plos.org/plosone/s/data-availability#loc-unacceptable-data-access-restrictions.

In your revised cover letter, please address the following prompts:

a) If there are ethical or legal restrictions on sharing a de-identified data set, please explain them in detail (e.g., data contain potentially sensitive information, data are owned by a third-party organization, etc.) and who has imposed them (e.g., an ethics committee). Please also provide contact information for a data access committee, ethics committee, or other institutional body to which data requests may be sent.

b) If there are no restrictions, please upload the minimal anonymized data set necessary to replicate your study findings as either Supporting Information files or to a stable, public repository and provide us with the relevant URLs, DOIs, or accession numbers. For a list of acceptable repositories, please see http://journals.plos.org/plosone/s/data-availability#loc-recommended-repositories.

We will update your Data Availability statement on your behalf to reflect the information you provide.

**Answer:** No primary data collection or analysis conducted. Information sourced from publicly available data, properly cited and referenced. No additional data set for sharing. Study findings can be replicated based on provided details.

**Reviewer #1:**

Dear Authors,

Your paper is interested and well structured, however some questions arose during the review process:

**Answer:** Authors firstly thank the respected reviewer to his/her beneficial comments. All comments, one by one, have carefully addressed in the revised article and also, answered in following paragraphs for next comments. Then, these words were also changed and highlighted in the revised article.

1. Introduction: More extensive presentation of Pareto optimization needs to be done with emphasis on the research in the presented research area.

**Answer:** Based on the reviewer's comment, we have updated the introduction section to include a more extensive presentation of Pareto optimization with a focus on its relevance to the presented research area.

*Fountas et al. [41] examined the performance of different swarm-based evolutionary algorithms in optimizing single and multi-objective problems related to additive manufacturing, specifically fused deposition modeling (FDM). The results showed that algorithms do not perform equally when applied to different optimization problems, and the quality of Pareto non-dominated solutions is an important indicator for multi-objective problems. Yodo and Dey [42] introduced multi-objective optimization methods based on evolutionary algorithms to optimize FDM process parameters, leading to Pareto-optimal solutions for decision-makers.*

2. Page 3 (P3): works are also done presenting the compressive loading of the PLA printed material - consider DOI: 10.3390/ma13194456

**Answer:** Thank you for bringing this article to our attention. We have added it to our literature review and have incorporated the findings into our introduction.

*Moreover, the influence of infill line distance on the compressive elastic behavior of 3D printed circular samples was investigated by Pepelnjak et al. [40] They observed a significant difference in the first cycle response and an alteration of stiffness due to elastic and plastic deformation under cyclic*

[40]	T. Pepelnjak, A. Karimi, A. Maček, and N. Mole, “Altering the Elastic Properties of 3D Printed Poly-Lactic Acid (PLA) Parts by Compressive Cyclic Loading,” *Mater*, vol. 13, no. 19, p. 4456, 2020, doi: 10.3390/MA13194456.

3. Recently a review of the influence of printing parameters on mechanical properties was published: consider their findings: https://doi.org/10.3390/polym15030716

**Answer:** The authors carefully considered respected reviewer’s comment and incorporated the relevant publication into our revised manuscript, enhancing our analysis of the printing parameters' impact on mechanical properties. We appreciate your suggestion, as it has contributed to the overall improvement and significance of our study.

*3D printing process parameters have a significant impact on the mechanical properties of printed parts, including printing temperature, speed, filling density, pattern, layer thickness, material properties, surface roughness, and layer thickness [31-33].*

[32]	T. Pepelnjak, J. Stojšić, L. Sevšek, D. Movrin, and M. Milutinović, “Influence of Process Parameters on the Characteristics of Additively Manufactured Parts Made from Advanced Biopolymers,” *Polymers (Basel).*, vol. 15, no. 3, p. 716, 2023, doi: 10.3390/POLYM15030716.

4, Material and material properties - you mention two references. However, are all those material properties from your table 1 experimentally determined?

**Answer:** We appreciate the respected reviewer's comment on the source of material properties in Table 1. The values were obtained from the material manufacturer's datasheets, except for the endurance limit which was experimentally tested by Dada. We have addressed this clarification in the revised manuscript.

*Density, elastic module, yield strength, ultimate tensile strength, and Poisson ratio were obtained from technical datasheets provided by the material manufacturer. These properties are typically reported by the manufacturer based on standard testing methods [47]. Though, endurance limit, which refers to the maximum stress amplitude that a material can withstand for a given number of cycles without failure, cannot be obtained from technical datasheets and requires experimental testing. The endurance limit used in this study [48] was experimental obtained.*

5. In chapter 2-2 the test specimen also numerically verified is also dimensional presented. It has diameter of 9 mm with a shell of 0,4 mm on its surface. How you can then claim that the element size of 0.7 mm is optimal for your evaluations. This is highly doubtful and needs further explanation. Additionally, with this ratio of specimen diameter versus element size you have only 13 elements on the cross-section. Finally, the selection of the element size needs to be discussed more into the detail.

**Answer:** We acknowledge the reviewer's valid concern about element size and low element count on the cross-section. To address this, we adjusted the element size using software parameters and ensured an adequate representation of the specimen's geometry. Specifically, we set the minimum size control and curvature control parameters to address thin areas and maintain accuracy. The resulting mesh contained more than 13 elements, providing reliable results.

*There were some areas with thickness less than 0.7 mm. In such cases, to prevent the distorted elements in these thin areas, the minimum size control parameter (set to 0.1 of the global size) and the curvature control parameter (set with a maximum deviation factor of 0.1) adjusted the element size. Increasing the maximum deviation factor allows for a greater deviation between the actual geometry and the approximated geometry, and can be useful in ensuring mesh quality in thin regions. The minimum size control and curvature control parameters used in this study were recommended by the ABAQUS software algorithm.*

6. Figure 2 - font in the upper row is too large.

**Answer:** In response to the respected reviewer’s comment, we have revised Figure 2 to adjust the font size in the upper row to ensure consistency with the font size in the lower row. The modified figure could be seen as follows,

|  |  | |  |
| --- | --- | --- | --- |
| (a) | (b) | | (c) |
|  | |  | |
| (d) | | (e) | |

**Fig 2: Different filling patterns: (a) triangular, (b) rectangular, (c) honeycomb, (d) irregular octagon, and (e) regular octagon**

7. Figure 5 presents the used machine for specimen printing. The scale is missing on the figure. The data about the printer's accuracy is missing.

**Answer:** The device size is added to Figure 5 to indicate the printer's dimensions to address the respected reviewer’s comment. The modified figure could be seen as follows,

**Fig 5: The FDM 3D printer used for additive manufacturing of the specimens**

8. It is noted that the samples have been printed 5x larger as the ones used in the CAD model. Why. This needs to be explained into the detail. Furthermore, the comment about the influence of this scaling is missing.

**Answer:** The decision to print the samples at a larger scale was driven by limitations in accurately printing complex geometries at a smaller scale. While this allowed to achieve the desired accuracy, the reviewer's point about the influence of scaling is valid. The authors have included a discussion in the revised version to address the need for future investigation on the influence of scaling on geometrical accuracy and mechanical properties of lattice structures.

*To ensure optimal results and utilize the capabilities of an industrial-duty 3D-printer machine, the samples were printed at a scale of 5 times larger than the original CAD models. One other reason is due to the machine limitations. This approach allowed to produce lattice structures with circular sections and a diameter of 45 millimeters, which would have been challenging to achieve with smaller scales. While this approach allowed for the successful printing of the specimens, it is important to investigate the potential effects of scaling on the accuracy of the geometrical features and the resulting mechanical properties in future investigations. Previous studies demonstrated clear size effects on the mechanical response of additively manufactured lattice structures [58].*

9. Table 7: first column is too narrow and cuts the text - correct this. Also in this table, you present the Von Mises stress distributions. Can you compare them also with the experimental data? From this comparison it is also not evident what is compressive and what tensile loaded. This need to be corrected and need also further explanation.

**Answer:** We appreciate the reviewer's feedback. The column width in Table 7 will be adjusted to prevent text truncation. Additionally, the contour stress in the direction of sample axis (σyy) is included in Table 8 to demonstrate the distribution of normal stress. While it is not feasible to experimentally determine stress distribution throughout the entire fatigue life, ongoing research is being conducted to measure the fatigue behavior of PLA material. This clarification has been added to the revised article in response to the reviewer's comment.

*As shown in stress distribution contours, the bending load generates compressive stress on the bottom side of the clamped end and tensile stress on its top side. However, in fatigue testing, the load is cyclic and changes the loading direction periodically, which leads to alternating regions of compressive and tensile stresses. Therefore, the specimens undergo both compressive and tensile stresses during fatigue testing, which can affect their durability and mechanical performance. Although the stress distribution in PLA specimens under cyclic loading cannot be measured experimentally, the fatigue behavior of the material is still worth investigating in future researches. This information can be compared to numerical results.*

**Table 7: The von-Mises stress distribution contours for different patterns**

| Pattern | 40% | 60% | 80% |
| --- | --- | --- | --- |
| Honeycomb |  |  |  |
| Rectangular |  |  |  |
| Triangular |  |  |  |
| Regular octagon |  |  |  |
| Irregular octagon |  |  |  |
| Full filled (100%) |  | | |

10. Numerical simulation: it is obligatory to present the used FE mesh for all shapes used in the comparative evaluations. Furthermore, you claim in the text that some parts are compressive and some tensile loaded. Are the tensile and compressive material properties the same. The simulations of the compressive printed parts were done by Karimi et al in https://doi.org/10.3390/app12168018 where also influence of the printed shape of the particular layer was analyzed. What shape of the particular layer have you selected? Perpendicular of the real one?

The Pareto optimization is done correct and there I have no comments.

**Answer:** We appreciate the valuable feedback. The compressive behavior of PLA was not addressed in this study, but we will incorporate relevant studies discussing this aspect in the revised manuscript.

*The compressive and tensile behaviors of PLA are usually considered to be the same, but some studies have focused specifically on the compressive behavior of PLA. These studies aim to better understand how PLA responds to compressive forces and how it can be used in applications where compression is a dominant mode of loading, such as in bone tissue engineering and orthopedics [49-50].*

[50]	A. Karimi, N. Mole, and T. Pepelnjak, “Numerical Investigation of the Cycling Loading Behavior of 3D-Printed Poly-Lactic Acid (PLA) Cylindrical Lightweight Samples during Compression Testing,” *Appl. Sci.*, vol. 12, no. 16, p. 8018, 2022, doi: 10.3390/APP12168018.

Regarding the shape of the particular layer selected for analysis the rectangular pattern with 60% filling density was chosen based on the results obtained from the two-objective optimization, based in Section 3-2 and Table 5, as mentioned before in the first version of the manuscript, as follows,

*Considering the interaction of objective functions, in the rectangular pattern with 60% of the filling density, the fatigue life has decreased by only 22%, despite the 39% reduction in mass. In addition, this sample has the shortest distance to the optimal point in the results of two-objective optimization by selecting the third set of objective functions (in Table 5) with an emphasis on the second objective. Based on this, fatigue samples should be printed with a 60% rectangular pattern.*

11. Figure 11 - again only the Von Mises stress is presented. Why?

**Answer:** To provide a more comprehensive understanding of the stress distribution in the component, we have added Figure 13 in the revised manuscript to include not only the von Mises stress but also the stress components in the directions of the component axis.

*Figure 13 displays the stress distribution along the component axis in optimized geometries. The contour lines in the figure accurately represent the stress distribution, including stress concentration regions, mirroring the pattern observed in the von-Mises stress. Additionally, the figure distinctly reveals areas with compressive stresses.*

(a)

(b)

(c)

**Fig 13: Stress distribution in the direction of cylinder axis in the optimized component after (a) topology optimization and (b) shape optimization (c)** **design realization**

12. Figure 12 - the scales need to be larger and more readable. From this figure it is also evident that the printing quality is not good. How this quality influence the properties of the real part and even more important - how you can implement those errors in the numerical simulations.

**Answer:** The scale of Figure 12 (Figure 14 in revised version) is increased in revised version of the article. Additionally, we recognize that the quality of 3D-printed parts can impact their properties. While this study focused on infill patterns, it is crucial to consider other sources of anisotropy, such as changes in printing paths and print parameters, which can affect the final product's quality and properties. These aspects should be considered during the design process.

*Based on the microscopic images, different kinds of anisotropy can significantly impact the quality and properties of 3D-printed parts, and the infill patterns examined in this study is one such example. The infill patterns, often referred to as meta-structures, play a crucial role in influencing the properties of the components [56].* *The infill patterns, often referred to as meta-structures, play a crucial role in influencing the properties of the components. Additionally, there are other forms of anisotropy that can arise from changes in printing paths or inadequate print parameters. While these factors were not specifically investigated in this study, it was reported from the porosity analysis that the adhesion between the laminated layers has no significant role to play in flexural properties [6].*

13. Figure 13 - the measured distances and angles are vague and not accurate. Comment such results.

**Answer:** In addressing the respected reviewer's concern about measurement accuracy, we used ImageJ software to measure the lines and angles multiple times, ensuring reliable results within an acceptable tolerance and standard deviation.

*As illustrated in Figs 15 and 17, the length of the vertices and width of lines were measured in the microscopic images to visualize dimensional accuracy. Specifically, Fig 15 shows a schematic of the measured line width and line length.*

*Fig 16 shows the measured values in an irregular octagon as a sample. ImageJ software was used to measure the length and width of the lines, as well as the angle between them. The process repeated three times to ensure accuracy. The results were within a reasonable tolerance based on the difference between the demanded and measured values as well as the standard deviations according to Table 12. The comparison between the average values measured from the microscopic images and corresponding CAD models is done in this table. The purpose of this analysis was to assess how accurately the 3D printer was able to reproduce the intended geometry of the parts, and to identify any potential sources of error or variability in the printing process. By comparing the actual dimensions of the printed parts with the intended dimensions, it is possible to gain insight into the overall quality and reliability of the printing process, and to identify opportunities for improvement.*

14. Conclusions: you mention the weight after 50 cycles. Does this changes through the cyclic loading? Furthermore - how is this weight comparable to the ones calculated from the geometry of the CAD model with corresponding material density. What is the real material density of your used material?

**Answer:** The optimization process experienced multiple design cycles, as shown in S1 Fig, with the final optimized geometry achieved after 50 cycles. Regarding the material properties, the values reported by the manufacturer (density, tensile modulus, yield strength) were obtained through standard tests for solid PLA material, as stated in the revised version.

*The optimized geometry in Fig 9 was reached after 50 design cycles. An animation is also included in supporting information (Fig S1) that shows the topology optimization process developments during each design cycle, providing a visual representation of the design process. Such visual support presents a better understanding of the design evolution and the optimization steps taken to arrive at the final design.*

*The properties of 3D printed parts can differ from those of the raw filament material. For example, while the density of PLA filament was typically reported as 1.24 g/cm^3^ in manufacturer datasheets based on the ASTM D792 standard test method, 3D printed parts can have a different density due to their meta-structure. Additionally, the tensile module and yield strength of PLA filament are reported as 3.5 GPa and 60 MPa, respectively**, based on ASTM D1238 standard test method. However, 3D printed parts may exhibit different values for these properties due to the complex interplay of factors such as infill percentage, layer height, printing speed, and other printing parameters.*

**Reviewer #2:**

Dear Authors,

in your interesting manuscript, the following points should be added/changed to further improve it.

**Answer:** The authors are glad to hear that reviewer found the work interesting and appreciate suggestions on how to improve the article. The comments are carefully considered and the necessary revisions are made to address the reviewer’s concerns. These changes were highlighted in the text.

- Introduction: "The robust objective function was weighted mean and variance of the deterministic objective" - what is meant here?

**Answer:** Thank you for your insightful comments and suggestions regarding our manuscript. We have carefully considered your feedback and made the following revisions to improve the clarity and quality of the article:

*Agrawal et al. [14] proposed a robust topology optimization method for negative Poisson ratio metamaterials, ensuring stable designs with low variation.*

- Section 2.2: Why were these infill patterns chosen and not, e.g., gyroid, which was found most suitable in experiments dealing with PLA as shape memory polymer?

**Answer:** We appreciate the respected reviewer's comment regarding our choice of infill patterns in the study. Our decision was based on previous research, which indicated that the infill patterns we selected (cubic, triangular, hexagonal, star, and grid) are commonly used and have been shown to have different mechanical properties. However, we agree that the gyroid pattern could be a promising candidate for future investigations in our research area. We have added a discussion of this point in the revised manuscript.

*The use of additive manufacturing enables the fabrication of different patterns with varying mechanical properties. The filling parameters have a direct effect on the response of the metamaterials to external loadings. For instance, a recent study [51] on metal functionally graded gyroids employed three design methods (thickness graded, size graded, and uniform) to provide a map of the mechanical properties of stainless-steel scaffolds. In our study, we selected five infill patterns, including honeycomb, rectangular, triangular, irregular octagon [51], and regular octagon [51] to investigate their effect on fatigue properties and mass, and the results suggest that further investigations on other patterns may be necessary, to compare the fatigue behavior. The infill patterns used in this study are shown in Fig 2.*

- Section 2.2: I assume the "thickness of 0.4 mm for each layer" is actually a width of 0.4 mm; else the layer thickness would be identical to the typical nozzle width.

**Answer:** The respected reviewer is correct that the term "thickness" is inaccurate and we have modified the sentence to read "width of 0.4 mm for each layer" in the revised version of the manuscript.

*To achieve desired filling densities these geometries were arranged next to each other at different distances. In addition, two solid layers are considered as a perimeter with a width of 0.4 mm for each layer [52].*

- Table 4: What is the difference between "all data" and "mean value"?

**Answer:** Thank you for your comment regarding Table 4. The "all data" column in Table 4 refers to the S-N diagram that fits all the data points from the fatigue tests conducted at each stress level. On the other hand, the "mean value" column in Table 4 refers to the average of the results at each stress level, which is then used to generate a new S-N diagram. I hope this clarifies the difference between the two columns in Table 4.

*In this table, fatigue tests were conducted at each stress level three times. Then, an S-N diagram was provided that fitted all the data. Moreover, the average of the results at each stress level was calculated and then again, another S-N diagram was provided.*

- Table 5: All "100" must be "100%". Please think about renaming "Delta W" to "Delta W/W_solid" (ditto for Delta N_f) as your Deltas are relative deviations given in %, while a Delta value is normally assumed to be an absolute difference.

**Answer:** Thank you for your comment. We have updated Table 5 to rename the mentioned parameters to more accurately reflect their percentage-based nature. Additionally, we have corrected the formatting of '100' to '100%' in the table. We hope that these changes have improved the clarity of the table.

**Table 5: The combination of objective functions**

| *Set* | $f_{1}$ | $f_{2}$ |
| --- | --- | --- |
| *Set 1* | *max:* $N_{f}={0.5\times\left( \frac{\sigma_{a}}{\sigma_{f}^{'}} \right)}^{\frac{1}{b}}$ | *min:* $M=\rho V$ |
| *Set 2* | *min:* $\Delta N_{f}\%=\frac{N_{f\left( solid \right)}-N_{f}}{N_{f\left( solid \right)}}\times100\%$ | *min:* $\Delta M\%=-\frac{M_{solid}-M}{M_{solid}}\times100\%$ |
| *Set 3* | *min:* $S={\Delta N}_{f}\%+\Delta M\%$ | *min:* $D=\sqrt{\left( \Delta N_{f}\% \right)^{2}+(\Delta{M\%+100\%)}^{2}}$ |

- Eqs. 8-11 do not look logically correlated; please check again where you have 1, 2, M, M-1 (or M+1).

**Answer:** We appreciate the respected reviewer’s attention to detail and feedback. We agree with you that the equations 8-11 were not clearly correlated with the introduced parameters. To clarify this issue, we have modified the manuscript and introduced the parameters as follows:

Let $M$ denote the number of Pareto non-inferior solutions, and let $x^{m} (m = 1,2,...,M)$ denote the set of these solutions.

- Fig. 4: What do the green lines mean?

**Answer:** In response to the respected reviewer's comment regarding the meaning of the green lines in Fig. 4, we would like to clarify that these lines represent the desired part of the print samples that were evaluated through inspection in the FESEM. This information has been added to the revised manuscript, and we thank the reviewer for bringing this to our attention.

*The green lines in Fig 4 represent the desired part of the print samples that was cut and evaluated through inspection in the FESEM.*

- "It should be noted that due to the complex shapes, samples were printed 5 times larger than CAD models based on the

capability of machine." - why? Which dimensions do the samples have and how many filled top/bottom layers and perimeters? And why was no commercially available printer used?

**Answer:** To address the reviewer's comment, the decision to print the samples at a larger scale was made to leverage the capabilities of an industrial 3D printer, enabling accurate and precise printing of complex lattice structures. The chosen infill patterns were designed in the CAD model and printed with 100% infill for clear visualization of the print path during subsequent experimental testing.

*To ensure optimal results and utilize the capabilities of an industrial-duty 3D-printer machine, the samples were printed at a scale of 5 times larger than the original CAD models. This approach allowed to produce lattice structures with circular sections and a diameter of 45 millimeters, which would have been challenging to achieve with smaller scales. Since the infill patterns were designed in the CAD model, the samples were printed with 100% infill and no perimeters or upper/lower layers, allowing for clear visualization of the print path.*

*The samples were printed with 100% infill and no top and bottom solid layers printed, allowing for clear visualization of the print path.*

- Fig. 6b: The y-axis shows the mass, not the weight of the samples. What do you mean in the corresponding text with "unpredictable relationship" - actually this is quite clear and logical.

**Answer:** The reviewer's correction regarding the use of the term "mass" instead of "weight" has been acknowledged and will be implemented in the paper. Additionally, the following discussion about mass of different filling pattern were added to the revised article, as follows,

*Based on the results shown in Fig 6(b), the mass of the honeycomb and rectangular lattice structures was nearly identical at various infill densities, with the rectangular pattern being slightly heavier at 40% infill. In addition, the triangular pattern exhibited less mass compared to the other three patterns. The irregular octagon, regular octagon, and rectangular patterns were the heaviest at 40%, 60%, and 80% infills, respectively.*

- Fig. 7: Where is the difference between blue and green points?

**Answer:** The green and blue points in Fig 7 represent different orientations of the lattice structures during printing. The green points correspond to vertical specimens, where the lattice is printed in a vertical orientation. This issue could be seen in the figure legend. Moreover, this is highlighted in the revised version of the article to address the respected reviewer’s comment.

*In Fig 7, the Pareto front indicates that there was not much change in the fatigue lifetime of vertical specimens (green points) even with a severe reduction in mass. On the other hand, the horizontal orientation (blue points) resulted in layer lines being placed horizontally in the direction of the bending force, making it the optimal orientation for mechanical strength. Despite the need for support during printing, this orientation provides the best mechanical strength.*

- Fig. 9: Does this optimized structure take the strong anisotropies in FDM printing into account?

**Answer:** In response to the respected reviewer's question regarding whether the optimized structure in Fig. 9 takes into account the strong anisotropies in FDM printing, it should be noted that while the infill patterns (meta-structures) were specifically designed to improve the mechanical properties of the printed parts, other forms of anisotropy arising from changes in printing paths or inadequate print parameters were not specifically investigated in this study. However, the importance of considering these factors during the design stage was emphasized in the revised version of the article.

*Based on the microscopic images, different kinds of anisotropy can significantly impact the quality and properties of 3D-printed parts, and the infill patterns examined in this study is one such example [56].* *The infill patterns, often referred to as meta-structures, play a crucial role in influencing the properties of the components. Additionally, there are other forms of anisotropy that can arise from changes in printing paths or inadequate print parameters. While these factors were not specifically investigated in this study, they should not be disregarded during the design stage, as they can have a notable impact on the performance of the final products.*

- Fig. 11: How can the optimized shape be FDM printed?

**Answer:** The printed specimen, demonstrating the optimized shape, has been included and visually depicted in Figure 11.

*The printed specimen in Fig 11 exhibits the desired geometry and structural features as intended in this design. This physical realization served as a tangible representation of the proposed model and validated the effectiveness of the present design process. It provided concrete evidence that the optimized design can be successfully translated into a physical object through 3D printing.*

**Fig 11: The designed sample printed based on the result of topology optimization**

- Table 10: The second column is "Mass (g)".

**Answer:** The reviewer's correction about using "mass" instead of "weight" in our paper has been acknowledged, and the necessary updates will be made to reflect this correction.

- Fig. 12: How are the air voids visible here taken into account in the simulation?

**Answer:** Air voids in 3D-printed parts, including those shown in Fig. 12, can impact quality and properties. This study examined infill patterns, but other voids from printing paths or parameters also affect performance. Design considerations must address these factors for optimal product performance.

*Based on the microscopic images, different kinds of voids can significantly impact the quality and properties of 3D-printed parts, and the infill patterns examined in this study is one such example [56].* *The infill patterns, often referred to as meta-structures, play a crucial role in influencing the properties of the components. Additionally, there are other forms of defects that can arise from changes in printing paths or inadequate print parameters. While these factors were not specifically investigated in this study, they should not be disregarded during the design stage, as they can have a notable impact on the performance of the final products.*

- Fig. 14: Which sorts of defects do you see in (b) and (c)? At least there are no air voids.

**Answer:** Higher magnification image presented in Fig 14(b) (Fig 16(b) in the revised version of the article), provided a clearer view of the defect. Based on the images presented in Fig 14 (Fig 16 in the revised version of the article), the defects observed are likely due to inconsistent fusion caused by imbalanced extrusion feed rate, extrusion temperature, and printing speed. These factors can lead to weakened mechanical properties and compromised performance of the printed part. It is crucial to consider these factors and optimize printing parameters to minimize defects and achieve the desired properties in 3D-printed parts.

*As observed in Fig 16(b), some small spherical-shaped pores may be generated during the fabrication of the filament, which can negatively impact the part's mechanical properties. Additionally, the extrusion feed rate plays a critical role in determining the quality of printed parts, as shown in Fig 16(c). If the extrusion feed rate is not balanced with the extrusion temperature and printing speed, inconsistent fusion can occur, resulting in defects. Another parameter that can affect the quality of printed parts is the distance between the nozzle and the build platform [70]. This distance can impact the adhesion between the layers and can cause defects similar to those shown in Fig 16(c). It is essential to consider these factors during the design and optimization of 3D-printed parts to achieve the desired mechanical properties and avoid potential defects.*

|  |  |  |
| --- | --- | --- |
| (a) | (b) | (c) |

**Fig 16: The defects observed in the samples with (a) 60% infill regular octagon, (b) 80% infill rectangular, and (c) 60% infill irregular octagon**

- Fig. 15: The length unit is wrong. Length and angle values have much too high accuracy; you can definitely not measure with 6 significant digits accuracy on such an image.

**Answer:** In the revised version, we have corrected the length unit in Fig 15 (Fig 17 in the revised article). For measurement in Fig 17, ImageJ software was used to measure length, width, and angle. The process was repeated three times for accuracy, and standard deviations were calculated. The measured values in Table 12 demonstrate a reasonable tolerance and effectively represent the shape and dimensions of the irregular octagon in Fig 17.

*Fig 17 shows the measured values in an irregular octagon as a sample. ImageJ software was used to measure the length and width of the lines, as well as the angle between them. The process repeated three times to ensure accuracy. The results were within a reasonable tolerance based on the difference between the demanded and measured values as well as the standard deviations according to Table 12.*

|  | ID | Length (mm) | Angle (degree) |
| --- | --- | --- | --- |
|  | 1 | 3.329 | 146.4 |
|  | 2 | 3.087 | 85.7 |
|  | 3 | 3.526 | 3.4 |
|  | 4 | 3.067 | 38.8 |
|  | 5 | 3.463 | 92.6 |
|  | 6 | 3.556 | 145.9 |
|  | 7 | 3.300 | 0.2 |
|  | 8 | 3.402 | 60.0 |

**Fig 17: The dimensional accuracy of the irregular octagon, measured from FESEM micrographs of the 3D-printed PLA with 60% of infill**

- Table 11: Where do these values stem from? Where are the units? Where do the standard deviations belong to? How should a printed line, typically of 0.4 mm width, have a demanded width of 2.75 mm etc., and what is meant with the line length? Besides, it would be good to correlate the measured infills (surely not measured with this accuracy) with the measured sample masses.

**Answer:** In response to the reviewer's comment, the measured line width in Table 11 (Table 12 in the revised version) represents the width of the printed structure's walls, measured using ImageJ software. Multiple measurements were taken at different locations to ensure accuracy and consistency. Following explanations were added in the revised version of the article to address the comment,

*As illustrated in Fig 15 and 17, the length of the vertices and width of lines were measured in the microscopic images to visualize dimensional accuracy. Specifically,* *Fig 15 shows a schematic of the measured line width and line length. The measured line width in this picture does not refer to the actual line width produced by the nozzle. Rather, it is the width of the walls in the structure, which may consist of multiple print paths. Fig 16 shows the measured values in an irregular octagon as a sample. ImageJ software was used to measure the length and width of the lines, as well as the angle between them. The process repeated three times to ensure accuracy. The results were within a reasonable tolerance based on the difference between the demanded and measured values as well as the standard deviations according to Table 12. The comparison between the average values measured from the microscopic images and corresponding CAD models is done in this table. The purpose of this analysis was to assess how accurately the 3D printer was able to reproduce the intended geometry of the parts, and to identify any potential sources of error or variability in the printing process. By comparing the actual dimensions of the printed parts with the intended dimensions, it is possible to gain insight into the overall quality and reliability of the printing process, and to identify opportunities for improvement.*

- "Based on the scatterplot in Figure 16, a strong positive and linear relationship between the two variables" - which ones?

**Answer:** In response to the respected Reviewer's comment on Figure 16 (Fig 18 in revised version), a clear positive and linear relationship is between demanded and measured line widths and lengths.

*Based on the scatterplot presented in Fig 18, it is evident that there exists a strong positive and linear relationship between the demanded and measured line widths (Fig 18(a)) as well as the demanded and measured line lengths (Fig 18(b)). This indicates that as the demanded line width or line length increases, there is a corresponding increase in the measured values, highlighting the consistent and proportional behavior between the two variables in the printing process.*

- Fig. 16: all texts are too small to be readable.

**Answer:** Figure 16 (Fig 18 in revised version) modified to improve text readability.

(a)

(b)

**Fig 18: The scatter plot of demanded values versus the measured data for (a) the line length and (b) the line width**

- Conclusion: The information that a sample with infill density 60% is 39% lighter than the 100% sample sounds logical, but does not fit to the measured infill percentages according to Table 11. It is thus really necessary to defined how you measured this value.

**Answer:** In the revised version of the article, it is clarified that the infill percentages were measured using ImageJ software based on microscopic images, providing accurate insights into the amount of infill material in each sample. The weight reduction comparison is based on calculated weight values considering material density and measured volumes in the CAD model.

*The comparison of weight reduction in this study is conducted by considering the calculated weight values, taking into account the theoretical density of the material and the measured volumes of the samples in the CAD model. By utilizing these parameters, the weight of the objects can be estimated.*

*Fig 16 shows the measured values in an irregular octagon as a sample. ImageJ software was used to measure the length and width of the lines, as well as the angle between them. The process repeated three times to ensure accuracy. The results were within a reasonable tolerance based on the difference between the demanded and measured values as well as the standard deviations according to Table 12. The comparison between the average values measured from the microscopic images and corresponding CAD models is done in this table. The purpose of this analysis was to assess how accurately the 3D printer was able to reproduce the intended geometry of the parts, and to identify any potential sources of error or variability in the printing process. By comparing the actual dimensions of the printed parts with the intended dimensions, it is possible to gain insight into the overall quality and reliability of the printing process, and to identify opportunities for improvement.*

The conclusion part is also modified as follows,

*The study introduced three sets of objective functions for design purposes, considering mass changes, fatigue lifetime changes, and the distance from the optimal point. The optimal solution was found to be the horizontally printed rectangular filling pattern with a 60% infill density. This pattern achieved a 39% reduction in weight while experiencing only a 22% decrease in fatigue lifetime. The mass reduction was determined through software analysis of designed CAD models, while the infill density was assessed using imageJ software.*

- The conclusion is too long.

**Answer:** Thank you for your feedback. We worked on maintaining a concise and clear conclusion that effectively summarizes the study's key findings and implications.

*This research used multi-objective optimization to determine optimal parameters for PLA cylinders, considering fatigue lifetime, mass, and infill patterns. Structural topology optimization was conducted using a two-objective algorithm. Additionally, FESEM analysis ensured dimensional accuracy, and the influence of inherent defects in the FDM process was considered. The results are listed below:*

- *The stress analysis results showed that although the triangular pattern had the lowest mass in all cases, irregular octagon, rectangular and regular octagon had the lowest stresses in 40%, 60%, and 80% of the infill, respectively.*
- *The study introduced three sets of objective functions for design purposes, considering mass changes, fatigue lifetime changes, and the distance from the optimal point. The optimal solution was found to be the horizontally printed rectangular filling pattern with a 60% infill density. This pattern achieved a 39% reduction in weight while experiencing only a 22% decrease in fatigue lifetime. The mass reduction was determined through software analysis of designed CAD models, while the infill density was assessed using the ImageJ software.*
- *Topology optimization in Dassault Systems software proposed a metamaterial structure to minimize mass and strain energy under stress constraints. Achieved results: mass of 1.1767g, strain energy of 0.056 J (after 50 cycles), maximum von-Mises stress of 97.08 MPa. Microscopic inspections studied manufacturing defects and their impact on topology optimization.*
- *Shape optimization was performed to reduce stress concentration by displacing boundary nodes. Maximum von-Mises stress decreased to 44.30 MPa after 15 design cycles, leading to increased fatigue lifetime by 527 and 79 cycles compared to topology optimization in vertical and horizontal samples, respectively.*
- *Finally, a design realization step was performed, and a new geometry was introduced that has 96.70% lower stress and 201.69% higher mass compared with topology-optimized geometry. This is while this geometry is lighter than 60% infill samples with different patterns.*
- *Based on FESEM images, the rectangular pattern has the best regularity, while the regular octagon pattern was the most irregular.*
- *The line spacing is significant, especially in the honeycomb pattern, and should be controlled by printing temperature.*
- *The scatterplot shows a strong positive and linear relationship between the demanded and measured line length and line width.*
- *Inaccuracy in the position of the nozzle increases with the decrease in filling percentage and, therefore, the thickness of the walls. However, the rectangular sample with 40% infill and Regular Octagon with 60% infill was the worst parameters.*

**Reviewer #3:**

The author has done good work. The work is related to “multi-objective numerical optimization of 3D-printed polylactic acid bio-metamaterial based on topology, filling pattern, and infill density via fatigue lifetime and weight” and has provided good insights on fatigue life time for the Additively manufactured printed prototypes. Here are some suggestions which the author can incorporate in the manuscript for improvement in the output of this work.

**Answer:** The authors express their gratitude to the respected reviewer for their valuable comments. Each comment has been carefully addressed in the revised article, and corresponding responses have been provided in the subsequent paragraphs. The changes were also highlighted in the revised article.

1. Author should provide scope of the work and literature gap as separate heading for better understanding

**Answer:** Thank you for the suggestion. The journal's template does not include a separate section for the scope of the work and literature gap. However, we have addressed and discussed these aspects at the end of the Introduction.

*The article discusses the multi-objective numerical optimization of 3D-printed polylactic acid (PLA) bio-metamaterial. The optimization takes into account the factors of topology, filling pattern, and infill density in order to improve the fatigue lifetime and mass of the material. Therefore, the novelties of the study are as follows,*

- *The use of multi-objective optimization to find the optimum value of topology, filling pattern, and infill density for 3D-printed polylactic acid bio-metamaterial.*
- *The consideration of both fatigue lifetime and mass in the optimization process.*
- *The use of field emission scanning electron microscopy (FESEM) images to investigate the dimensional accuracy of 3D-printed components.*

2. What is bio-metamaterial? Bio material is separate term whereas Meta material is something related to design-based objective of materials.

**Answer:** In the revised version of the article, bio-metamaterials are defined as follow,

*Bio-metamaterials, designed with geometrical structures at smaller scales, mimic the properties of living tissues and are applied in orthopedic biomaterials for bone tissue regeneration [11].*

3. Figure 6a shows the 100% infill density part has shown stress capacity <10MPa. But the original value for PLA material lies somewhere between 30-70MPa. Why the predicted value is many times lower than the actual value?

**Answer:** The reviewer correctly points out that the original yield strength of PLA material is higher than the stress capacity observed in Figure 6a. However, in high cycle fatigue testing, it is necessary to avoid stress levels exceeding the yield strength to prevent plastic deformation or permanent damage. Our study intentionally operated within a stress range below the yield strength to accurately evaluate the fatigue performance and durability of the 3D-printed parts without compromising their structural integrity.

*In the rotating-bending test, a common method used to evaluate the high cycle fatigue behavior of materials, the specimen is subjected to cyclic loading through rotational motion. During the rotating-bending test, the applied stress levels are typically kept below the yield strength of the material. This ensures that the deformation experienced by the specimen remains within the elastic range (<70 MPa [47]), where the material can fully recover its original shape after each cycle. The focus is on assessing the material performance to withstand cyclic loading without undergoing plastic deformation or failure.*

4. Whereas Figure 6b is not important as it is well established fact that when density increases the weight will increase.

**Answer:** In response to the respected reviewer's comment, the revised article includes the following information,

*Based on the results shown in Fig 6(b), the mass of the honeycomb and rectangular lattice structures was nearly identical at various infill densities, with the rectangular pattern being slightly heavier at 40% infill. In addition, the triangular pattern exhibited less mass compared to the other three patterns. The irregular octagon, regular octagon, and rectangular patterns were the heaviest at 40%, 60%, and 80% infills, respectively*

5. No practical work has been performed other than mathematical relations and numerical simulation. You should 3D print any material and test it with your optimized condition and verify the predicted model using real environment condition?

**Answer:**

In response to the reviewer's comment the following information is added to the revised version of the article,

*In this research, the emphasis was primarily on numerical simulation and optimization of 3D-printed materials. The results were utilized to determine the parameters for experimental tests, the data of which has already been published [59-60].*

6. One real 3D printed object based on optimized or suggested model should be printed for PLA material and the model should be verified to find the accuracy of the model.

**Answer:** We thank the reviewer for their suggestion. We have followed their advice and printed a real 3D object based on the optimized model for PLA material. We have also verified the accuracy of the model by comparing the dimensions of the printed object with the simulation results. We have added this information and a new figure (Figure 11) to the revised manuscript to demonstrate the validity of our approach.

**Fig 11: The designed sample printed based on the result of topology optimization**

7. Previous literature work or result should be compared with the proposed model result for validation purpose along with suitable graph of validation.

**Answer:** In response to the reviewer's comment, following discussions were added to the revised version of article,

*The literature supports that the outer shell affects bending strength. Samples with ABS: TPU: ABS sandwiched structure showed higher flexural strength due to ABS material in outer layers, aiding compression and expansion during loading [5].*

8. Following literature may be added to enhance the literature review

1. Kumar S, Singh I, Kumar D, Yahya MY, Rahimian Koloor SS. Mechanical and Morphological Characterizations of Laminated Object Manufactured 3D Printed Biodegradable Poly (lactic) acid with Various Physical Configurations. Journal of Marine Science and Engineering. 2022 Dec;10(12):1954.

2. Kumar S, Singh I, R. Koloor SS, Kumar D, Yahya MY. On Laminated Object Manufactured FDM-Printed ABS/TPU Multimaterial Specimens: An Insight into Mechanical and Morphological Characteristics. Polymers. 2022 Sep 28;14(19):4066.

3. Kumar S, Singh R, Singh TP, Batish A. Comparison of mechanical and morphological properties of 3-D printed functional prototypes: Multi and hybrid blended thermoplastic matrix. Journal of Thermoplastic Composite Materials. 2022 May;35(5):692-707.

4. Kumar S, Singh R, Batish A, Singh TP. Additive manufacturing of smart materials exhibiting 4-D properties: a state of art review. Journal of Thermoplastic Composite Materials. 2022 Sep;35(9):1358-81.

**Answer:** To address the respected reviewer’s comment, the recommended studies were considered and have incorporated related works that align with the focus and scope of our research in the revised version of the manuscript.

[5]	S. Kumar, I. Singh, S. S. R. Koloor, D. Kumar, and M. Y. Yahya, “On Laminated Object Manufactured FDM-Printed ABS/TPU Multimaterial Specimens: An Insight into Mechanical and Morphological Characteristics,” *Polymers (Basel).*, vol. 14, no. 19, p. 4066, 2022, doi: 10.3390/POLYM14194066.

[6]	S. Kumar, I. Singh, D. Kumar, M. Y. Yahya, and S. S. Rahimian Koloor, “Mechanical and Morphological Characterizations of Laminated Object Manufactured 3D Printed Biodegradable Poly(lactic)acid with Various Physical Configurations,” *J. Mar. Sci. Eng.*, vol. 10, no. 12, p. 1954, 2022, doi: 10.3390/JMSE10121954.

[10]	S. Kumar, R. Singh, A. Batish, and T. P. Singh, “Additive manufacturing of smart materials exhibiting 4-D properties: A state of art review,” vol. 35, no. 9, pp. 1358–1381, 2019, doi: 10.1177/0892705719895052.

**Reviewer #4:**

The manuscript represents good potentials for readers. All section represents technically correct data. Analysis is technically sound. Data is supported by relevant literature. Authors have taken nice efforts to fulfil requirements of Journal.

**Answer:** Thank you for your positive feedback and recognition of the manuscript's potential and technical accuracy. We are glad to hear that the analysis is deemed technically sound and supported by relevant literature. We greatly appreciate your acknowledgment of our efforts in meeting the requirements of the journal. Your positive evaluation encourages us to continue our work and strive for excellence.

**Reviewer #5:**

The study is well conducted and results have been theoretically discussed. This manuscript could be accepted in its current form. The execution of the experimentation and testing is at par with the current research requisites.

**Answer:** Thank you for your positive evaluation of the study. We are pleased to hear that the manuscript's content and results have been well conducted and theoretically discussed.

**Reviewer #6:**

The focus of this work is studying optimization of 3D-printed polylactic. The present work does not include any novel materials or methods. This manuscript is insufficient of novelty or significant contribution for publication as all materials and methods have been published in the last 3 years, see the references. In conclusion, the innovation of the manuscript is very scarce.

**Answer:** We thank the reviewer for comments and concerns. As we stated at the end of the introduction, our main novelties are as follows,

*The article discusses the multi-objective numerical optimization of 3D-printed polylactic acid (PLA) bio-metamaterial. The optimization takes into account the factors of topology, filling pattern, and infill density in order to improve the fatigue lifetime and mass of the material. Therefore, the novelties of the study are as follows,*

- *The use of multi-objective optimization to find the optimum value of topology, filling pattern, and infill density for 3D-printed polylactic acid bio-metamaterial.*
- *The consideration of both fatigue lifetime and mass in the optimization process.*
- *The use of field emission scanning electron microscopy (FESEM) images to investigate the dimensional accuracy of 3D-printed components.*

Additionally, the conclusion of the study has been modified to emphasize the significance of the innovations,

*This research used multi-objective optimization to determine optimal parameters for PLA cylinders, considering fatigue lifetime, mass, and infill patterns. Structural topology optimization was conducted using a two-objective algorithm. Additionally, FESEM analysis ensured dimensional accuracy, and the influence of inherent defects in the FDM process was considered.*

Apart from the lack of contribution, it has two major issues that are lack of any design of experiments for 3D printing process and loading in a cyclic manner for this particular application. That is why we see quick failure under loading. I would strongly suggest the authors to carry out a proper design of experiments to see the effects of printing parameters like temperature of nozzle, speed of print head, fibre orientation, layers arrangement, infill pattern, etc. on the performance of the structure under mechanical loading. As it is under cyclic loading in the real application, the mechanical loading modelling must be cyclic as well.

A new version of the manuscript with all major fundamental changes could be re-submitted for review.

**Answer:** The authors agree that experimental testing and design of experiments are important aspects to consider in the 3D-printing process and the mechanical loading of the material. However, as we stated in our manuscript, in this research, the focus was on numerical simulation and optimization of 3D-printed materials, with the obtained results guiding the parameters for subsequent experimental tests, the details of which have already been published [59-60]. We hope that this will satisfy the reviewer’s concerns and expectations.

In order to address the respected reviewer’s comment, the following paragraph for the future works is added to the revised article, as follows,

*Designing for high-performance in additive manufacturing, despite its limitations, requires exploring various unit cells and meta-structures to identify patterns that interact effectively with material behavior.* *In this research, the emphasis was primarily on numerical simulation and optimization of 3D-printed materials. The results were utilized to determine the parameters for experimental tests, the data of which has already been published [59-60]. For further investigations, the following subjects are suggested,*

- *Path planning and defining the route that the print head takes*
- *Providing topology optimization algorithms to achieve desired properties for specific applications*
- *Using exactly the material properties of the meta-structure and anisotropy (considering the 3D printing factors) for the lifetime prediction*
- *Examine the fatigue lifetime experimentally for the topology-optimized samples.*
