# Supplementary material for: Multi-objective numerical optimization of 3D-printed polylactic acid bio-metamaterial based on topology, filling pattern, and infill density via fatigue lifetime and mass
Source: PLoS One. 2023 Sep 27;18(9):e0291021. doi: 10.1371/journal.pone.0291021 (PMC10529563; doi:10.1371/journal.pone.0291021)
Supplement: Attachment — Submitted filename: Answers-02.docx [file pone.0291021.s006.docx]

**Dear Editor-in-Chief,**

PLOS ONE

The authors thank you for giving another opportunity to revise my paper titled “Multi-objective numerical optimization of 3D-printed polylactic acid bio-metamaterial based on topology, filling pattern, and infill density via fatigue lifetime and mass”. We appreciate the valuable feedback and comments provided by the first reviewer, which have helped us to improve the quality and clarity of the manuscript. In this second revision letter, we have addressed all comments of the first respected reviewer and explain how we have done it in the revised version of the paper. We hope that the changes will sufficiently address the concerns raised and that the revised manuscript will be suitable for publication in PLOS ONE.

Regards,

**M. Azadi, PhD.**

Faculty of Mechanical Engineering, Semnan University, Semnan, Iran

**Reviewer #1:**

Dear authors,

you have improved the manuscript. However, there are still some opened questions which need to be resolved prior to the publication. Also, the English language needs to be improved.

**Answer:** Thank you for your feedback. We appreciate your acknowledgment of the improvements made to the manuscript. We will address the remaining questions and concerns and work on further improving the English language to ensure a more polished and comprehensive final version for publication.

1. What is changed here?

**Answer:** The term "weight" has been appropriately replaced with "mass" throughout the text.

2. Full stop is missing

**Answer:** The missing full stop has been added.

*Moreover, the influence of infill line distance on the compressive elastic behavior of 3D printed circular samples was investigated by Pepelnjak et al. [40].*

3. The density you could measure by yourself!

**Answer:** We appreciate the respected reviewer's suggestion. To ensure accuracy and reliability, we conducted density measurements, and the results aligned with the values provided by the manufacturer.

4. It is not clear: is here mentioned the study No. 48 or the study presented in the manuscript?

**Answer:** We apologize for the mistake. We modified the text in the revised version of the article as follows,

*The endurance limit used in this study was obtained experimentally from the reference [48].*

5. More accurate determination of Poisson ratio is presented in https://www.mdpi.com/2073-4360/15/7/1651

There it is also shown that the Poisson ratio is a function of infill density. have you considered that in your simulations?

**Answer:** No. But in order to address the respected reviewer’s comment the following explanation is added by using the mentioned reference in MDPI:

*Manufacturing parameters have a significant impact on material response, particularly for polymers [47-48]. For example, it was reported that the Poisson ratio is influenced by infill density [49]. However, in this study, the material properties of filament were used and the investigation of other parameters was not included.*

6. The boundary conditions on Figure 3a are not sufficient visible. Redraw the figure.

**Answer:** the schematic figure of the problem is replaced to address the respected reviewer’s comment and to ensure better visibility and clarity of the boundary conditions.

|  |  |
| --- | --- |
| (a) | (b) |

**Fig 3: (a) The illustration applied load plus boundary conditions and (b) the C3D8R element**

7. why with capital letter – rewrite

**Answer:** The capitalization issue has been addressed.

*The focus is on assessing the material performance to withstand cyclic loading without undergoing plastic deformation or failure. In this regard, static, general simulations were performed in Abaqus/Standard.*

8. Your nozzle diameter is 0.4 mm. However, the global size of elements does not fits in this size. Despite that you have mentioned some areas with elements having less than 0.7 mm in dimension it needs to be stated their size as well. Present at least the minimal size of the element and the number of elements over the critical dimension (like thickness).

Have you performed the mesh-size sensitivity analysis for your numerical simulation?

**Answer:** Thank you for your valuable feedback. It should be mentioned that we did not model the layered structure and print process in our simulation. As a result, the nozzle diameter does not directly affect the numerical analysis.

However, we performed a mesh sensitivity analysis to evaluate the impact of varying element sizes on the model's performance. The graph below depicted element size vs. von-Mises stress for the fulfilled specimen as a sample providing additional clarity to the reviewer.

**Mesh sensitivity analysis for solid sample**

The results showed that the von-Mises stress became stable once the element size reached 0.7 mm. When the element size was more than this value, stress values exhibited fluctuations and variations until they converged at 0.7 mm. Further reducing the element size did not significantly affect stress levels, indicating convergence. Therefore, an element size of 0.7 mm strikes a balance between accuracy and computational efficiency for this analysis. In addition, we have added the minimum size of the elements to enhance the clarity of our findings.

*The study aimed to analyze the impact of infill patterns and densities on printed part performance, without modeling of the layered structure or the print process. Therefore, the nozzle diameter was not directly considered in the numerical analysis. However, certain areas in the model had thicknesses below 0.7 mm, which required special consideration to prevent the element distortion. Therefore, two control parameters were utilized, including minimum size control which set to 0.1 of the global size and a curvature control parameter with a maximum deviation factor of 0.1. These adjustments ensured that elements in thin regions maintained high-quality meshing while adhering to the desired accuracy levels. The chosen control parameters were recommended by the ABAQUS software algorithm. Accordingly, Table 3 presents the minimum element length and the number of elements for various infill patterns and filling densities. Mesh sensitivity analysis was also conducted to assess the impact of varying element sizes on the performance of models. It was observed that the von-Mises stress became stable once the element size reached 0.7. As the element size reduced, the stress values showed fluctuations and variations until they reached the critical value of 0.7. Beyond this point, a further reduction in element size did not significantly affect the stress levels, indicating that the mesh had reached a point of convergence. This finding suggested that an element size of 0.7 can provide a balance between the accuracy and computational efficiency for the specific analysis of the model.*

**Table 3: The number of elements in different models**

| *Fill pattern* | *Infill (%)* | *Minimum size (mm)* | *Number of elements* |
| --- | --- | --- | --- |
| *Honeycomb* | *40* | *0.079* | *163947* |
|  | *60* | *0.139* | *93129* |
|  | *80* | *0.136* | *124653* |
| *Regular Octagon* | *40* | *0.065* | *118690* |
|  | *60* | *0.104* | *13961397* |
|  | *80* | *0.162* | *131262* |
| *Irregular Octagon* | *40* | *0.116* | *101232* |
|  | *60* | *0.180* | *93936* |
|  | *80* | *0.315* | *26418* |
| *Rectangular* | *40* | *0.090* | *102897* |
|  | *60* | *0.196* | *51837* |
|  | *80* | *0.400* | *22644* |
| *Triangular* | *40* | *0.033* | *149850* |
|  | *60* | *0.116* | *73704* |
|  | *80* | *0.109* | *90021* |
| *Solid* | *100* | *0.576* | *17538* |

9. How you can assure that these parameters are the proper ones for your case study?

**Answer:** The stability and convergence observed in the analysis demonstrate the suitability of the selected mesh resolution and control parameters, providing confidence in the accuracy and reliability of our numerical simulation.

*This finding suggested that an element size of 0.7 can provide a balance between the accuracy and computational efficiency for the specific analysis of the model.*

10. Find additional scientific literature dealing with this problem. So important issue cannot be supported by the user’s manual of the commercial program only. Also, the penalty factor was taken from the user’s manual - but is that one the proper one for your case?

**Answer:** We appreciate the reviewer's comment, and in response, we have included additional scientific literature related to the problem in revised version of article.

*Based on the literature the numerical experiments indicate that a penalty factor value of p = 3 is suitable in the SIMP method for the topology optimization [55-56].*

11. Explain into the detail what kind of modifications you have performed.

**Answer:** The detailed modifications made during the study have been thoroughly explained in section 3, specifically in the "Numerical Results and Optimization" part of the manuscript.

*The optimized geometry has a unique characteristic in its design - a quadruple thread-like pattern surrounded by a shell. This pattern is evident throughout the structure and adds a distinct visual aspect to the overall design. The quadruple thread-like pattern with its shell provides an optimized structure with improved mechanical performance by reducing mass and enhancing strength in specific locations.*

12. which ones - mention them here

**Answer:** The information regarding the magnifications, which include 15x, 22x, 27x, 30x, 80x, and 500x, was added in the revised version of the manuscript.

*Imaging was performed with magnifications of different magnitudes, including 15X, 22X, 27X, 30X, 80X, and 500X.*

13. Were here modelled only the approximate shapes of the printed parts or the real ones? The simulation of the real shape could be a demanding task.

**Answer:** We modeled and simulated the real shapes of the printed parts using ABAQUS software as shown in Table 2. Figure 4 in the manuscript illustrates the real shapes of the printed components that were used as input for the simulations.

*To investigate the influence of these parameters on the performance of printed parts, specimens with different filling patterns and densities were modeled in ABAQUS software, as shown in Table 2.*

14. How this maximum stress was obtained? It also needs to be defined on the diagram what kind of the stress is this.

**Answer:** The maximum von-Mises stress values were obtained from FEM simulations. Additionally, to address the comment about clarity, we have revised the diagram to explicitly define the stress type as "von-Mises stress" to ensure better understanding and accuracy for the readers.

*(a)*

*(b)*

***Fig 6: The influence of filling parameters on (a) the maximum bending stress and (b) the mass of structures***

15. The mass of here presented part does not present much. Put somewhere on the figure also the picture of the part.

**Answer:** To provide better visualization and context, we have incorporated a picture of the pattern on the above figure in the previous comment.

16. Is this for the 5x enlarged samples or those from the CAD part?

Why actually you have selected this part enlargement? Why you did not evaluate all results with sufficient large models?

**Answer:** Given the complex nature of regular and irregular octagons, it was necessary to upscale the specimens before 3D printing. The enlargement process allowed us to effectively capture the intricacies and finer details of these complex infill patterns using the 3D printer machine.

*To ensure optimal results and utilize the capabilities of an industrial-duty 3D-printer machine, the samples were printed at a scale of 5 times larger than the original CAD models. One other reason is due to the machine limitations. This approach allowed to produce lattice structures with circular sections and a diameter of 45 millimeters, which would have been challenging to achieve with smaller scales. While this approach allowed for the successful printing of the specimens, it is important to investigate the potential effects of scaling on the accuracy of the geometrical features and the resulting mechanical properties in future investigations.*

17. texts of all scales are too small

The entire table should use the same scale for the Mises stress. As it is currently presented the pictures of all cases are the same.

**Answer:** We have addressed the reviewer's feedback by making necessary adjustments to the table. The pictures have been enlarged to ensure better readability, providing improved clarity for readers. Furthermore, we have now standardized the scale for the Mises stress column across the entire table, avoiding any confusion caused by identical pictures for all cases.

***Table 7: The von-Mises stress distribution contours for different patterns***

| *Pattern* | *40%* | *60%* | *80%* |
| --- | --- | --- | --- |
| *Honeycomb* |  |  |  |
| *Rectangular* |  |  |  |
| *Triangular* |  |  |  |
| *Regular octagon* |  |  |  |
| *Irregular octagon* |  |  |  |
| *Solid (100%)* |  | | |

***Table 8: The stress distribution contours in Y direction (σ_yy_) for different patterns***

| *Pattern* | *40%* | *60%* | *80%* |
| --- | --- | --- | --- |
| *Honeycomb* |  |  |  |
| *Rectangular* |  |  |  |
| *Triangular* |  |  |  |
| *Regular octagon* |  |  |  |
| *Irregular octagon* |  |  |  |
| *Solid (100%)* |  | | |

18. vertical printed specimens?

**Answer:** The respected reviewer is correct and the vertical printed specimens were analyzed and there was not much change in the fatigue lifetime of vertical printed specimens despite a severe reduction in mass based on the Pareto front.

*In Fig 7,* *the Pareto front indicates that there was not much change in the fatigue lifetime of vertical printed specimens (green points) even with a severe reduction in mass.*

19. Do I understand correct that better were the specimens which were printed with the part's main axis in horizontal direction? If this is so, how you have succeeded the quality print of the empty places in the structure (in particular at 40% infill)?

**Answer:** The improved performance of specimens printed horizontally can be attributed to the alignment of the loading direction with the print direction. In vertical printing, the load is perpendicular to the print direction, potentially leading to reduced mechanical strength due to the layer-by-layer nature of 3D printing. In contrast, horizontal printing allows for better interlayer bonding and a favorable load transfer path, enhancing the mechanical properties of the printed parts.

About examining the structure, the manufacturing process involves printing the parts from the bottom layer to the top layer, and the infill patterns are arranged perpendicular to the print direction. As a result, the structure remains consistent for both print directions.

*It should be mentioned that horizontal printing improves mechanical strength due to aligned loading and better interlayer bonding, while vertical printing, with perpendicular load and layer-by-layer nature, may lead to reduced strength. This is while, both print directions maintain consistent structure from bottom to top layer* *[58].*

20. Sorry but I cannot see this from the Fig 8. Add the value to the Fig 8.

**Answer:** To address the respected reviewer’s comment, we have added the required value to Figure 8.

***Fig 8: The variations of design responses through design cycles***

21. What is this?

**Answer:** It is the ISO value that is explained in the text.

*A threshold of the ISO (MAT_PROP_NORMALIZED in the legend of Fig 9) value is generally used for regulating, which the finite elements are displayed. This value is utilized to obtain the positions on the finite element edges, where the new nodes are made and must be between zero and unity. Larger values lead to a model with a smaller volume.*

22. Why you compare your results with sandwich structure? If you do not have a sandwich structure delete this part; or it is necessary to express why you could compare your results with a sandwich structure.

**Answer:** In order to address the respected reviewer’s comment, the unrelated reference is deleted from the text in the revised version of the article.

23. How you can verify this stress in the specimen? This needs to be somehow verified to confirm the reliability of the optimization procedure.

**Answer:** Due to the intricate geometry resulting from the optimization process, it is not feasible to validate the stress values analytically, as analytical solutions are typically limited to simple geometries and loading conditions. We acknowledge the necessity of experimental investigations and recommended experimental tests on the physical specimen.

*The results represent an output of a complex geometry from the optimization process. However, experimental investigations are necessary to confirm the reliability and accuracy of the optimization procedure.*

24. Explain how the cutting was done - normally this represents a demanding issue.

**Answer:** Print samples were cut with a fretsaw along marked lines for analysis, ensuring precise removal without affecting surrounding areas.

*The printed samples were cut with a fretsaw along marked lines for the analysis, ensuring precise removal without affecting surrounding areas. Then, the cut samples evaluated by inspection in the FESEM, and then examined for dimensional and geometrical accuracies.*

25. Which ones? Why you use the FESEM for magnifications of 22x?

**Answer:** We used FESEM for magnifications of 22x, 15x, 27x, 30x, 80x, and 500x to capture different levels of detail. High magnification is necessary to study fine features and surface characteristics.

*In order to assure adequate electrical conductivity for FESEM observation, samples were coated with a golden layer. The imaging process was performed with different magnifications, including 15X, 22X, 27X, 30X, 80X, and 500X. These magnifications were employed to capture various levels of details, allowing for a more comprehensive evaluation of the studied samples.*

26. This shape is not a precise honeycomb at all. Define the shape accuracy measure and add it to all cases presented on Fig 14. Furthermore, it is to see that the honeycomb presented on the figure is not printed with cells in contact. How you can assure the bending strength of such printed part?

Additionally, have you considered those errors also in your ABAQUS simulations?

**Answer:** Shape accuracy measure was provided in Table 12 for all structures. Also, the lack of cells in contact in the honeycomb structure (Fig 14) is influenced by increased extrusion temperature, affecting adhesion between layers and filament sides in the filling pattern as explained in the text. In response to the reviewer's question regarding the consideration of voids and defects in the ABAQUS simulations, we did not consider voids and defects. The study mainly focused on numerically evaluating how the infill pattern and density affect the performance of 3D printed components.

**Table 12: The dimensional variations of samples printed as per the experimental design**

| Parameter / Fill pattern | Rectangular | | | Triangular | Honeycomb | Regular octagon | Irregular octagon |
| --- | --- | --- | --- | --- | --- | --- | --- |
| Demanded Infill (%) | 40 | 60 | 80 | 60 | 60 | 60 | 60 |
| Measured Infill (%) | 74.61 | 77.44 | 90.27 | 69.7 | 75.55 | 70.24 | 71.66 |
| Standard Deviation | 86.53 | 29.07 | 12.84 | 16.17 | 25.92 | 17.07 | 19.43 |
| Demanded Line Width (%) | 2.75 | 2.75 | 2.75 | 4.50 | 1.25 | 1.50 | 2.50 |
| Measured Line Width (%) | 2.78 | 2.57 | 2.41 | 4.36 | 1.49 | 1.54 | 2.39 |
| Standard Deviation | 0.12 | 0.13 | 0.09 | 0.17 | 0.10 | 0.10 | 0.15 |
| Demanded Line Length (mm) | 0.50 | 1.25 | 3.00 | 1.00 | 1.60 | 1.25 | 3.3 |
| Measured Line Length (mm) | 0.83 | 1.18 | 3.41 | 1.02 | 1.57 | 1.46 | 3.39 |
| Standard Deviation | 0.10 | 0.14 | 0.13 | 0.05 | 0.11 | 0.22 | 0.09 |

27. Considering all other irregularities how you can claim that this filament errors can affect the mechanical properties of the printed parts?

**Answer:** We acknowledge the reviewer's concern regarding the claim about filament errors and their impact on the mechanical properties of the printed parts. In response, we refer to literature [72], which highlights that moisture can significantly affect filament behavior.

*According to the literature [72], the moisture significantly affected the filament behavior.*

28. can you explain why?

**Answer:** The dimensional variations observed in these infill configurations were comparatively more significant than those observed in other patterns

*The rectangular sample with 40% of infill and the regular octagon with 60% of infill exhibited the highest deviations from the desired shapes.*

29. Fonts are too small.

**Answer:** We have addressed the respected reviewer's comment by increasing the font size in figure 18.

*(a)*

*(b)*

***Fig 18: The scatter plot of demanded values versus the measured data for (a) the line length and (b) the line width***

30. This is not true. With FESEM you have only analysed the printed shapes but definitely not assured the dimensional accuracy.

**Answer:** The study addressed dimensional accuracy by analyzing printed shapes using ImageJ software, presenting results in Table 12. FESEM provided visualizations, while ImageJ allowed for quantitative measurements, ensuring comprehensive evaluation and validating dimensional precision of printed components.

31. Why with capital letters?

**Answer:** The capitalization issue has been resolved. Thank you for bringing this to our attention
